# Genome‐Wide Characterization of Sex‐Linked Regions in the Sangzhi Horned Toad (*Boulenophrys sangzhiensis*) Reveals Complex Sex Determination Mechanisms

**DOI:** 10.1111/1755-0998.70136

**Published:** 2026-04-08

**Authors:** Chun H. Huang, Si Y. Xie, Jun Li, Wan Y. Chen, Fu Y. Qiu, Mian Zhao, Wei Liu, Chun L. Liao, Hua Wu

**Affiliations:** ^1^ Hubei Key Laboratory of Genetic Regulation and Integrative Biology, School of Life Sciences Central China Normal University Wuhan China; ^2^ Badagong Mountain National Nature Reserve Sangzhi China

**Keywords:** amphibian, chromosome‐level genome, putative sex‐determining gene, sex determination, sex‐linked region

## Abstract

Sex chromosomes in amphibians exhibit substantial variability and often remain largely homomorphic, providing a powerful system for studying sex determination. The Sangzhi horned toad (*Boulenophrys sangzhiensis*) is an ideal model, but limited genomic resources have hindered insights into sex determination mechanisms. Here, we assembled a 2.8 Gb chromosome‐level genome of *B*. *sangzhiensis* generated using PacBio HiFi and Hi‐C data, achieving a Contig N50 of 30 Mb and identifying 21,775 protein‐coding genes. Genome‐wide analyses of coverage, *F*_ST_, SNP density, and linkage disequilibrium identified two putative sex‐linked regions on chromosome 2 and 6, showing sex‐specific coverage and strong genetic differentiation. Within these sex‐linked regions, we identified a Y‐specific sequence and several candidate sex‐determining genes, including *Hsd11b2*, *Nlrp14*, and zinc finger genes. Our findings suggest that sex determination in *B*. *sangzhiensis* involves a complex, possibly polymorphic mechanism, with multiple Y haplotypes segregating within populations. Additionally, the sex‐linked regions exhibit accumulation of repetitive sequences, multi‐copy genes, and chromosomal rearrangements, which may contribute to the evolution of sex chromosomes. This chromosome‐level genome provides a valuable resource for understanding the dynamic evolution of sex‐determining mechanisms in amphibians.

## 1 Introduction

Sex determination is a fundamental process in developmental biology and molecular biology, with profound implications for conservation, ecology, and evolution (Ashman et al. 2014; Gower et al. 2019; Pečnerová et al. 2017; Louis et al. 2021; Bidon et al. 2014; Herrero et al. 2021). This biological process influences the levels of genetic variation, the degree of inbreeding, the ability of adaptation to novel environments, and the formation of new species (Ashman et al. 2014). Research has demonstrated that sex determination systems exhibit remarkable diversity and can vary independently across different taxa (Bachtrog et al. 2014; Ashman et al. 2014; Pan et al. 2016). In mammals and birds, the sex determination systems are highly conserved, relying on genetic sex determination triggered by highly conserved genes (Pennell et al. 2018; Graves 2008; Zhou et al. 2014). The stability and conservation of sex‐determination systems in mammals and birds may oversimplify our understanding, obscuring the diversity of mechanisms in other vertebrates. In contrast to the stable genotypic sex determination, the sex determination mechanisms of ectothermic animals, including reptiles, amphibians, and fish, often exhibit more variability and instability (Kitano et al. 2024; Gamble et al. 2015; Pennell et al. 2018; Evans et al. 2012; Jeffries et al. 2018; Ma and Veltsos 2021; Mank et al. 2006). Thus, the study of sex chromosomes in ectothermic animals provides a unique opportunity to uncover the underlying mechanisms of sex determination and refine existing models of sex chromosome evolution.

The diverse mechanisms of sex determination in amphibians make them a particularly fascinating group for studying the evolutionary processes underlying sex determination. Most amphibians possess genetic sex determination (GSD) systems, typically of the XX/XY or ZZ/ZW type (Eggert 2004; Hillis and Green 1990). These systems can sometimes be influenced by environmental factors, such as temperature, as well as by geographical variations among populations, suggesting that sex determination in amphibians is complex and environmental cues may act in concert with genetic mechanisms to regulate sexual differentiation (Miura 2008; Shen and Wang 2018). Rapid sex‐chromosome turnover has been clearly documented across amphibians (Rodrigues et al. 2018; Jeffries et al. 2018; Phillips et al. 2019; Ma and Veltsos 2021). These phenomena are often accompanied by diverse genetic mechanisms underlying sex determination, including the emergence of new sex‐determination genes (SDGs), chromosomal rearrangements, recombination suppression, polygenic modifiers, and interactions between genes and population phylogeography, which together further complicate the understanding of sex chromosome evolution in amphibians (Furman, Metzger, et al. 2020). For example, in *Rana temporaria* , sex‐chromosome differentiation and sex‐linkage vary geographically along a latitudinal transect in Sweden (Rodrigues et al. 2014, 2015, 2016). In the intermediate *R. temporaria* populations (e.g., Esrange and Häggedal), phenotypic sexes were mixed: some males carried a male‐specific marker, whereas others were genetically indistinguishable from females (Rodrigues et al. 2014). Sex reversal (XX males, XY females) has also been reported in *R. temporaria* (Rodrigues et al. 2014, 2015, 2016, 2017).

Despite the diversity of sex‐determining systems, the vast majority of sex chromosomes in amphibians are homomorphic and morphologically indistinguishable between the sexes (Eggert 2004; Stöck et al. 2011; Ma and Veltsos 2021; Dufresnes and Crochet 2022). The widespread occurrence of homomorphic sex chromosomes means that sex‐determination systems are generally identified through genetic rather than morphological evidence. The origin, maintenance, and evolution of these homologous chromosomes remain unresolved and are central to ongoing discussions in the field of sex chromosome evolution. Several hypotheses have been proposed to explain the persistence of homomorphic sex chromosomes in amphibians. One hypothesis suggests that homomorphic sex chromosomes may be maintained through frequent turnover of the master sex‐determining (MSD) genes (Hillis and Green 1990; Volff et al. 2007; Tanaka et al. 2007; Ross et al. 2009; Cnaani et al. 2008). Another ‘fountain of youth’ hypothesis posits sporadic X‐Y recombination occurs in sex‐reversed individuals (Perrin 2009; Matsuba et al. 2010; Stöck et al. 2011). These mechanisms are thought to prevent long‐term differentiation of sex chromosomes and may be driven by incomplete genetic control over sex determination (Grossen et al. 2011; Rodrigues et al. 2018).

The discovery of independently evolved sex‐linked regions (SLRs) in different species offers valuable insights into sex chromosome evolution. However, the specific mechanisms of sex determination in amphibians remain unclear. Early investigations into amphibian sex determination were constrained by limited‐resolution techniques, such as karyotyping, cytogenetics, and allozyme or microsatellite analyses (Hillis and Green 1990). Although methods like genotyping‐by‐sequencing (GBS) (e.g., RAD‐seq) improved locus‐level resolution (Sopniewski et al. 2019), identifying SLRs and SDGs remains challenging, especially in species with homomorphic sex chromosomes (Guerrero et al. 2012). Recent advances in genomic sequencing offer better prospects for overcoming these challenges. Therefore, using whole‐genome sequencing to compare genomic information between females and males may facilitate the accurate identification of sex chromosomes and SLRs, which is crucial for advancing our understanding of the origins and maintenance mechanisms of sex determination.

*Boulenophrys sangzhiensis* (Anura: Megophryidae) represents a valuable system for studying sex determination because it belongs to an early‐diverging anuran lineage (often referred to as Archaeobatrachia) (Feng et al. 2017; Pyron and Wiens 2011; Mahony et al. 2017). Given that many anurans possess homomorphic or weakly differentiated sex chromosomes, studying an early‐diverging lineage can help test hypotheses about the early stages of sex‐chromosome differentiation and assess whether patterns observed in derived frogs are broadly conserved. In this study, we assembled a high‐quality chromosome‐level genome of *B*. *sangzhiensis*, establishing a comprehensive genomic resource for future research. Using this genome as a reference, we: (1) identify two putative sex‐linked chromosomes/regions, (2) identify a Y‐specific sequence, (3) propose potential sex‐determining candidate genes, (4) report distinct Y haplotypes in *B*. *sangzhiensis* populations, and (5) provide evidence consistent with a role for transposable elements (TEs) and duplications in the formation of sex chromosomes in *B*. *sangzhiensis*. Our findings provided a critical foundation for further investigations into the mechanisms underlying the origin, maintenance, and evolution of homomorphic sex chromosomes.

## 2 Materials and Methods

### 2.1 Sample Collection and Identification

All samples (Table S1) used in this study were collected from two sites, Doupeng Mountain (DP) and Tianping Mountain (TP), located in the Badagong Mountain National Nature Reserve, Sangzhi County, Zhangjiajie, Hunan Province, China (Figure S1). For genomic sequencing, muscle tissue from one adult male was used for Illumina and PacBio HiFi sequencing, liver tissue from the same individual was used for Hi‐C library construction, and seven tissues (brain, liver, heart, kidney, muscle, gonad, and spleen) of the same individual were used for transcriptome sequencing and gene structural annotation. For whole‐genome resequencing, 20 males and 20 females were collected. For transcriptomic sequencing, 12 adult gonads (6 females and 6 males) were used. Details for sampling, identification, DNA/RNA extraction, and sequencing are in Appendix (Supporting Information Methods).

### 2.2 Sequencing, Assembly and Quality Assessment

A high‐quality chromosome‐level genome of *B. sangzhiensis* was assembled using Illumina short reads (200.6 Gb, ~79× coverage), PacBio HiFi long reads (85.4 Gb, ~30× coverage), and Hi‐C chromatin conformation data (245.6 Gb, ~88× coverage).

The genome size was estimated at approximately 2.55 Gb based on K‐mer analysis of Illumina reads. A preliminary assembly was produced using HiFiasm v0.19.6, followed by polishing and de‐redundancy with Purge Haplotigs v1.0.4, yielding a 2.87 Gb primary assembly. To confirm that the assembly represented the nuclear genome of the target species, contigs were aligned to the NCBI Nucleotide database using BLASTN v2.11.0. Only contigs showing extensive, high‐identity matches to prokaryotic or organellar sequences were removed, considering both alignment identity and coverage. The statistics of the short reads and Hifi reads mapping rate and coverage were summarized using BWA (v0.7.12‐r1039) (Li and Durbin 2009) and minimap2 v2.20 (Li 2018), respectively. Genome completeness was assessed by BUSCO V4.0.1 (Simão et al. 2015). Contigs were anchored into chromosomes using Juicer v1.6 (Durand et al. 2016) and 3D‐DNA v180922 (Dudchenko et al. 2017), yielding a 2.8 Gb *B. sangzhiensis* reference genome. Detailed sequencing and assembly protocols see Appendix.

### 2.3 Genome Annotation

Repetitive elements were annotated using RepeatMasker (http://www.repeatmasker.org), RepeatProteinMask (Tarailo‐Graovac and Chen 2009) (http://www.repeatmasker.org), LTR‐FINDER (Xu and Wang 2007), Trf (Benson 1999) and RepeatModeler (http://www.repeatmasker.org/RepeatModeler). Protein‐coding gene were annotated by integrating de novo prediction, homology‐based, and transcriptome‐based assembly, using tools like AUGUSTUS v3.4.0 (Stanke et al. 2006), Genscan (http://genes.mit.edu/GENSCAN.html), and MAKER2 (Carson and Mark 2011). Gene functions were assigned using several databases (e.g., KEGG, SwissProt, TrEMBL, COG, and NR) and assessed with BUSCO V4.0.1 (Simão et al. 2015). For non‐coding RNA annotation, tRNA was identified using the tRNAscan‐SE software (Lowe and Eddy 1997). MiRNA and snRNA were predicted by the INFERNAL software included in Rfam (Griffiths‐Jones et al. 2005). For detailed methods, see Appendix.

### 2.4 Haploid Assembly

Haploid genomes of a male *B. sangzhiensis* were assembled using Hifiasm v0.19.6 with combined HiFi and Hi‐C data, yielding two partially phased pseudo‐haplotypes: Hap1 (2.71 Gb) and Hap2 (2.74 Gb). Contig assignments were further refined using HapHiC to improve phasing accuracy and consistency. Assembly completeness was evaluated with BUSCO v4.0.1 (Simão et al. 2015) using the tetrapoda_odb10 database. For detailed methods, see Appendix.

### 2.5 Cytogenetic Karyotype Analysis

Metaphase chromosomes were prepared from bone marrow cells of adult female *B. sangzhiensis* following the method described in Xie et al. (2025). The detailed procedures are provided in Appendix. Metaphase chromosome spreads were examined and photographed under a BX63 upright differential interference contrast (DIC) microscope (Olympus, Japan).

### 2.6 Genomic Data and Chromosome Synteny Analysis

We used the newly assembled *B. sangzhiensis* genome (male), together with publicly available and in‐house genomes of related amphibians, including *Leptobrachium leishanensis* , *Bufo gargarizans* , *L. boringii* , *R. temporaria* , *Xenopus tropicalis* , *Bombina bombina* , and four additional *Leptobrachium* species. Details of the genome sources and accession numbers see Appendix.

To assess inter‐ and intra‐species chromosomal collinearity, orthologous gene pairs were identified using the JCVI toolkit (Tang et al. 2008), and syntenic blocks were filtered and visualized with jcvi.graphics.karyotype. Intraspecific synteny in *B. sangzhiensis* was assessed via all‐vs‐all BLASTP followed by MCScanX analysis, with Circos plots generated in TBtools (Chen, Chen, et al. 2018). Structural rearrangements were detected using SyRI (Goel et al. 2019) based on pairwise genome alignments produced by Minimap2 (v2.20) and were visualized using Plotsr. For detailed methods, see Appendix.

### 2.7 Resequencing, Variants Calling and Filtering

Paired‐end libraries were constructed from 20 females and 20 males and sequenced on the MGI DNBSEQ‐T7 platform (BGI Tech, Shenzhen, China), achieving an average sequencing depth (MeanDepth) of 16.1× per individual (Table S3). Raw reads were quality‐checked and filtered using FastQC v0.11.3 and fastp (Chen, Zhou, et al. 2018). Clean reads were aligned to the reference genome using BWA (v0.7.12‐r1039) (Li and Durbin 2009) and processed with SAMtools v1.9 (Li et al. 2009) and Picard v2.1 for sorting and duplicate removal. Variants were called using GATK4 and filtered based on standard criteria. After filtering, 36.26 million SNPs and 6.55 million InDels were retained for downstream analyses. SNPs in high linkage disequilibrium (LD) were removed, resulting in an LD‐pruned SNP dataset. Detailed procedures and parameter settings see Appendix.

### 2.8 Identify SLR

Sex chromosome and SLRs were identified by integrating genomic coverage (Palmer et al. 2019), SNP density, sex‐linked SNPs (Palmer et al. 2019), and genetic differentiation (*F*_ST_) analysis (Gammerdinger et al. 2020; Toups et al. 2019; Rodrigues et al. 2017; Liu et al. 2024). Genome‐wide coverage was estimated using PanDepth v2.19, and the log_2_(M:F) coverage ratio was used to detect SLRs. SNPs were filtered and SNP density calculated using VCFtools (Danecek et al. 2011). Sex‐linked SNPs were defined based on allele frequency differences. *F*_ST_ values between sexes were calculated. Windows within the top 1% of *F*_ST_ values were considered candidate SLRs. Details are provided in Appendix.

### 2.9 Estimation of Linkage Disequilibrium

In order to investigate patterns of recombination, LD was analysed in *B*. *sangzhiensis*. Based on the flitered vcf file (‐‐maf 0.05 ‐‐geno 0.25), we calculated the LD decay across the SLR on chromosome 6 (Chr 6) in males and females as well as the unsex‐linked region of sex chromosome and autochrome using PopLDdecay (Zhang et al. 2019). In addition, plink (v1.9) (Chang et al. 2015) was used to calculate pairwise *r* ^2^ for all SNPs located in the SLR on chromosome 2 (Chr 2). Pairwise *r* ^2^ values were then summarized and plotted.

### 2.10 Genotype Heatmap

Based on candidate 375,874 sex‐linked SNPs identified from 5 females and 5 males in TP population, we generated a genotype heatmap for the SLR on Chr 6. Given the large number of SNPs detected on Chr 6, the dataset was filtered by removing all missing genotypes (./.) and retaining only loci showing complete genotype consistency within each sex (i.e., all males or all females shared the same genotype of 0/0, 0/1, or 1/1). This filtering yielded 43,103 loci, of which 42,690 exhibited heterozygosity (0/1) in males and homozygosity (0/0 or 1/1) in females, consistent with an XY‐type inheritance pattern. A subset of 20,000 loci was randomly selected for visualization, revealing a pronounced male‐heterozygous and female‐homozygous block across the SLR on Chr 6.

We also examined Chr 2; however, the number of sex‐linked SNPs in this region was much lower (79 loci, 26.3 SNPs/Mb) than that in Chr 6 (84,637 loci, 2015.2 SNPs/Mb). Therefore, a genotype heatmap for Chr 2 was not generated. Nevertheless, integrated evidence from LD analysis, male‐biased genomic coverage, and Y‐specific sequence identification suggests that Chr 2 may contain small, degenerating Y‐linked segments, suggesting that this chromosome may also be involved in the ongoing process of sex chromosome differentiation in *B. sangzhiensis*.

### 2.11 Male‐Specific Marker Exploitation

We used BWA software (Li and Durbin 2009) with default parameters to align the female and male sequencing reads to the male genome assembly. The resulting aligned files were processed with SAMtools software (Li et al. 2009) to convert them to BAM files and to calculate sequencing depth using PanDepth software (v2.19). We then extracted male‐specific regions on Chr 2 by identifying sequences covered exclusively by male reads and absent in female reads (Figure 2) (Chen et al. 2014). These male‐specific regions were visualized using the IGV software (Robinson et al. 2011).

To verify the male‐specific marker obtained above, we designed a pair of male‐specific primers (F208: CGTGACTGCCAGCATTAACG, R379: TTGACTTCAGAGCAGCCGTT) of sex‐specific fragments using the Geneious software (Kearse et al. 2012). We then amplified the sex‐specific region using genomic DNA samples from 14 males and 10 females. The PCR conditions were 5 min at 95°C; 35 cycles of 95°C for 30 s, 63°C for 30 s and 72°C for 45 s and, last, incubation at 72°C for 5 min. All PCR products were analysed on 2.5% agarose gels by agarose gel electrophoresis. To further confirm the specificity, PCR products from 5 randomly selected male individuals were purified using a gel extraction kit (Vazyme, China) and sequenced by Sanger sequencing. The obtained sequences were aligned to the genome using the mapping function in Geneious software to confirm their correspondence to the target male‐specific sequences.

### 2.12 Analysing Genetic Structure of *B. sangzhiensis*

LD‐pruned SNPs were used for population structure inference. ADMIXTURE v1.23 (Alexander et al. 2009) was run with default settings to estimate individual ancestry proportions, testing hypothetical clusters (K) from 2 to 10. The optimal K was selected based on the lowest cross‐validation (CV) error. Principal component analysis (PCA) based on genome‐wide SNPs and SNPs within SLRs was conducted using Plink v1.90 (Purcell et al. 2007).

### 2.13 Co‐Occurrence of Sex‐Linked Differentiation on Chr 2 and Chr 6

To evaluate whether sex‐linked signals on Chr 2 and Chr 6 co‐occur within the same individuals, we conducted complementary population‐level and individual‐level analyses. Because male sampling in the DP population was much more limited, the primary analyses focused on the TP population, with DP used as supportive evidence only.

PCA based on SNPs in the SLRs of Chr 2 and Chr 6 revealed two distinct male clusters in TP population, M1 (12 males) and M2 (5 males). For each cluster, we recalculated *F*_ST_, log_2_(M:F) coverage ratios, and SNP density or identified sex‐related SNPs to assess sexual differentiation (window = 50 kb). The co‐occurrence of Chr 2 and Chr 6 differentiation signals within the same cluster suggests partial linkage or population‐level association between the two regions. However, given the unphased nature of the assembly and lack of trio data, these results should be interpreted as co‐occurrence rather than direct physical linkage.

To assess whether the same individuals carried male‐associated signals on both Chr 2 and Chr 6, we exported resequencing genotypes from filtered biallelic SNP VCFs using VCFtools v0.1.16 (‐‐012). We then identified ‘XY‐like’ loci (females fixed for the same homozygous genotype together with male heterozygosity enrichment) and computed per‐individual sex‐linkage scores for the Chr 6 and Chr 2 SLRs (Table S14 and Figure S20). As a sensitivity analysis, we additionally screened ‘XY‐specific (strict)’ loci (all males heterozygous; all females fixed homozygous) and the female‐specific (ZW‐specific) loci (Table S14). Full details are provided in Appendix.

### 2.14 Comparative and Structural Analysis of the Candidate Gene

The copy number and chromosomal location of gene were identified based on genome annotation, OrthoFinder (v2.5.4), and Minimap2 (v2.20) analyses. Multiple sequence alignment and phylogenetic reconstruction were performed using MUSCLE v3.8.1551 (Edgar 2004) and IQ‐TREE v2.0.3 (Minh et al. 2020), respectively. The three‐dimensional protein structure was predicted with AlphaFold3 (https://alphafoldserver.com/) and visualized in PyMOL v3.1.3 (DeLano 2002). Appendix for further details.

### 2.15 Transcriptome Sequencing and Analysis

Gonadal tissues were collected from 6 adult females and 6 adult males. Total RNA was extracted using standard protocols and sequenced on the Illumina platform (See Appendix). Clean reads were mapped to the unphased *B. sangzhiensis* reference genome using HISAT2 (Kim et al. 2015), and read counts per gene were obtained with featureCounts (Liao et al. 2014). Differential gene expression was analysed with DESeq2 (Love et al. 2014), applying a false discovery rate (FDR)–corrected *q*‐value < 0.05 and |log_2_(fold‐change)| > 1 as significance thresholds to define sex‐biased genes.

Sex‐biased genes were integrated with genomic differentiation metrics, including SNP density, *F*_ST_, and coverage, to identify candidate genes involved in sex determination and differentiation. See Appendix for further details.

## 3 Results and Discussion

### 3.1 Sequencing, Assembly and Annotation

**FIGURE 1:**
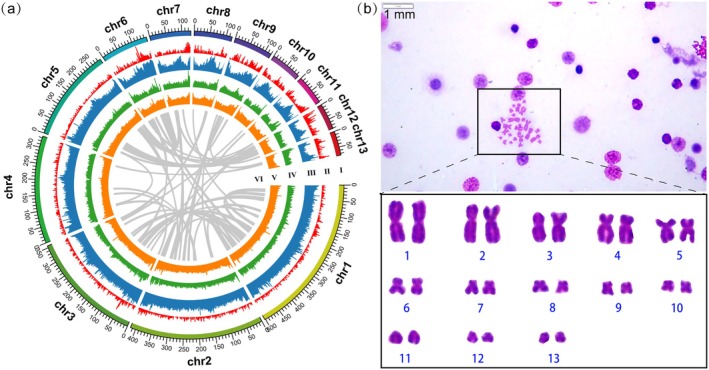
(a) Circular plot of the chromosome‐level genome assembly of *B*. *sangzhiensis*. From the outer circle to the inner circle, genome characteristics are indicated successively, including chromosome sizes (I), gene density (II), the genome‐wide percentages of repeat sequence (III), SNP density (IV), GC content (V), and synteny (VI). All the data were calculated in a 500 kb window. (b) Karyotype of female *B. sangzhiensis*. Metaphase spread and karyogram show 13 pairs of chromosomes (2*n* = 26), consistent with the chromosome‐scale genome assembly.

We have generated the first high‐continuity, chromosome‐length de novo assembly of the *B*. *sangzhiensis* genome by combining Illumina short linked reads, HiFi long‐reads, and Hi‐C data. The final Hi‐C hybrid assembly is 2.78G in size, with an N50 scaffold length of 335 Mb and an N50 Contig length of 30 Mb (Table S9). The GC content of the assembly is 44.27%. A total of 21,775 protein‐coding genes were identified, with gene counts on each chromosome ranging from 885 to 3043. Most of the predicted genes (95.87%) were functionally annotated against known protein databases (Table S11). In total, 2.76 Gb of sequences were clustered into 13 chromosomes (2*n* = 26) (Figures 1a and S3), with chromosome sizes ranging from 75.46 to 502.50 Mb. This result is consistent with previous reports that closely related species, including *L. boringii* , *L. leishanensis* , and others, possess 13 chromosomes (Li 2007; Li et al. 2019; Xie et al. 2025; https://evobir.shinyapps.io/AmphibianDB/). In addition, synteny analysis with closely related species showed no obvious large‐scale chromosomal fusion (Figure S4). Karyotype analysis of female *B. sangzhiensis* confirmed the presence of 13 chromosome pairs, consistent with our chromosome‐level assembly (Figure 1b). Genome completeness was assessed using BUSCO, which indicates that 96.6% of the vertebrate gene set is present and complete (Table S8). These results collectively confirm the chromosomal accuracy and completeness of the *B. sangzhiensis* genome assembly. Overall, we generated high‐quality genome sequences, providing a good foundation for identifying sex determining (Figure 1).

A total of 1.86 Gb of repetitive sequences were identified in *B*. *sangzhiensis*, comprising 67.85% of the genome assembly (Table S10), predominantly consisting of transposable elements (TEs, 65.33%). Long Terminal Repeats (LTRs) represent the most dominant TE elements (33.43%). TEs, especially LTR retrotransposons, are highly active genetic elements that replicate via reverse transcription and integrate into various genomic regions, thereby increasing genome plasticity and diversity (Troskie et al. 2021; Schrader and Schmitz 2019). Moreover, TEs have been implicated in the evolution of SLRs. In fish, SLRs are often enriched with transposon sequences, suggesting that TEs may influence the regulation of SDGs (Chalopin et al. 2015). In *B*. *sangzhiensis*, the high abundance of repetitive sequences, particularly LTRs, may contribute genetic material for forming or modifying SLRs, potentially influencing sex differentiation. LTR expansion could regulate gene expression by providing additional regulatory elements that interact with sex‐specific genes, thereby promoting the development of sexually dimorphic traits (Dechaud et al. 2019). This mechanism may offer greater flexibility for sex determination in *B*. *sangzhiensis*. The role of TEs in sex determination warrants further investigation.

To further characterize the genomic architecture, we generated two pseudo‐haplotype assemblies (Hap1 and Hap2) using HiFiasm in Hi‐C–aware mode (Figures S5 and S6). Whole‐genome synteny analysis revealed strong one‐to‐one correspondence between Hap1 and Hap2 across most chromosomes, confirming high structural consistency of the genome. Local structural variations, including inversions (INV), translocations (TRANS), and duplications (DUP), were also detected (Figure S7), providing additional insights into fine‐scale chromosomal rearrangements.

### 3.2 Identification of SLR

**FIGURE 2:**
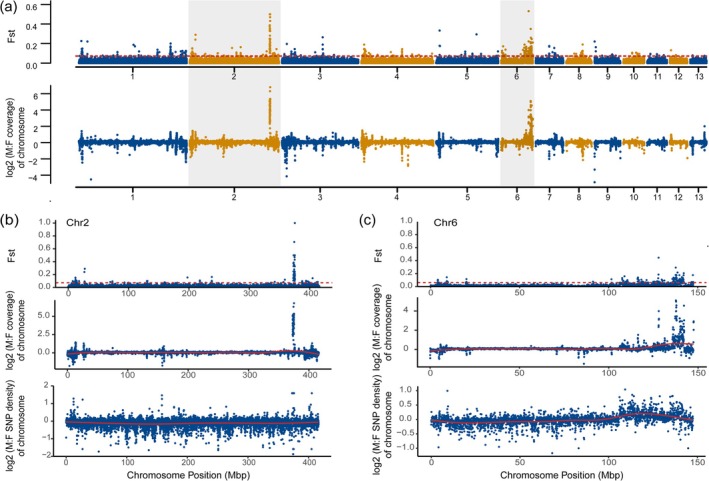
(a) Manhattan plot visualization of SNP‐based *F*_ST_ values (top) and log_2_(M:F) coverage ratios (bottom) across the 13 chromosomes of 20 females and 20 males in *B*. *sangzhiensis*. The top track shows the mean *F*_ST_ values, with a dotted line representing the top 1% cutoff between females and males. The bottom track shows the male‐to‐female coverage ratio. (b) Detailed view of Chr 2 showing SNP‐based *F*_ST_ (top), log_2_(M:F) coverage ratios (middle), and log_2_(M:F) SNP density (bottom). (c) Similar plots for Chr 6, including SNP‐based *F*_ST_ (top), log_2_(M:F) coverage ratios (middle), and log_2_(M:F) SNP density (bottom). All metrics (e.g., coverage, *F*_ST_, SNP density) were calculated within 50 Kb sliding windows across each chromosome.

To identify the SLRs of *B*. *sangzhiensis*, whole‐genome resequencing reads from 20 individuals per sex were aligned to the *B. sangzhiensis* reference genome. We conducted read coverage for both male and female and *F*_ST_ values based on genome‐wide SNPs between sexes. Distinct sex‐biased coverage and elevated genetic differentiation were observed on Chr 2 and Chr 6, suggesting that both are candidate sex chromosomes of *B*. *sangzhiensis* (Figure 2a). Notably, while large regions of genetic differentiation were observed on Chr 6, smaller but significant sex‐linked segments were also identified on Chr 2. A small region, approximately 3 Mb (372.8–375.6 Mb), on Chr 2 exhibits a pronounced peak of *F*_ST_ and log_2_(M:F) coverage ratios, making it a strong candidate for a SLR (Figure 2b). Additionally, a larger region of about 41.6 Mb (105.9–147.5 Mb) on Chr 6 also shows clear sexual differentiation, characterized by *F*_ST_, SNP density, log_2_(M:F) coverage ratios (Figure 2c).

To ensure the robustness of analyses, the quality of the genome assembly was assessed in terms of accuracy, completeness, and structural consistency (Tables S6–S8; Figures S3, S5, and S6). We further examined resequencing read‐level breakpoint signatures (supplementary/split alignments, soft‐clipping and zero‐depth patterns) at both SLR boundaries on Chr 2 across individuals and found no evidence of a local mis‐assembly (Table S15). Multiple lines of evidence strongly support the biological origin of the dual sex‐linked signals, making a methodological artifact unlikely. Analyses of GC content (Figure S8), InDel distribution (Figure S9), and read‐depth profiles (Figure S11) further confirmed that the observed log_2_(M:F) coverage ratios and *F*_ST_ signals were unlikely to be artefactual.

The observation of sex‐linked SNPs/markers occurring on two distinct chromosomes is rare among frogs. Although cases of species possessing two sex‐linked chromosomes have been reported, these typically reflect different populations utilizing distinct sex chromosomes, rather than multiple sex chromosomes coexisting within the same individuals. For example, *R. pipiens* exhibits sex‐linkage on Chr 2 and Chr 5 in different populations across eastern and western North America (Ma and Veltsos 2021). Similarly, *R. japonica* employs Chr 1 in western Japan and Chr 3 in eastern Japan, indicating intraspecific polymorphism (Ma and Veltsos 2021). Exceptionally, in *R. temporaria* , multiple sex‐linked chromosomes have been shown to coexist in northern Swedish populations (Toups et al. 2019), suggesting a polygenic sex‐determination system. Subsequent studies proposed that *R. temporaria* retains ancestral sex chromosomes alongside more recently evolved ones (Rodrigues et al. 2016), representing a transitional phase in sex chromosome evolution. An extreme example is the Amazonian frog *Leptodactylus pentadactylus* , which possesses six pairs of sex chromosomes in addition to five pairs of autosomes (Gazoni et al. 2018). Collectively, these highlight that the presence of multiple or polymorphic sex chromosomes in *B. sangzhiensis* is biologically plausible and may reflect an ongoing sex chromosome turnover or a hybrid origin (Schartl et al. 2023).

In amphibians, most species exhibit extreme heterochiasmy, where male recombination is largely restricted to chromosomal ends, whereas recombination in females occurs genome‐wide (Brelsford et al. 2016; Sardell and Kirkpatrick 2020). Such a pattern may partly explain why SLR on Chr 2 of *B. sangzhiensis* is located along the chromosomal arm rather than at the terminal end. In contrast, Chr 6 harbours a terminal SLR, resembling the pattern reported in *R. temporaria* populations from Ammarnäs and Kilpisjärvi (Toups et al. 2019). One plausible explanation is that TE‐mediated structural rearrangements (segmental duplications or inversions) locally inhibit recombination (Section 3.5) and facilitate the formation of new SLRs (Zheng et al. 2025; Matsuda et al. 2002; Hua‐Van et al. 2005; Furman, Metzger, et al. 2020; Gray 2000). Previous studies have shown that small, structurally distinct Y‐linked blocks can evolve independently of the genome‐wide recombination pattern, particularly in species with homomorphic sex chromosomes (Furman, Metzger, et al. 2020). Such independently evolving localized sex‐linked blocks may indicate a more complex trajectory of sex chromosome evolution in *B. sangzhiensis*.

Within these candidate SLRs on Chr 2 and Chr 6, we observed hemizygous Y‐linked regions, with an approximately ten‐fold excess of male over female reads (Figures 2b,c and S11). These regions may be a nonresolved Y‐specific copy number variation in the assembly. In addition, we observed corresponding sequences (e.g., 112.1–115.5 kb on Chr 6) between males and females displaying similar read coverage between males and females (Figure 2c), consistent with low levels of sex chromosome degeneration (Jasonowicz et al. 2022). In comparison to the SLR in congener *L. boringii* , SLR in *B*. *sangzhiensis* is smaller and exhibits lower levels of divergence (Xie et al. 2025). This phenomenon of low degradation is also evident in some fishes. For example, in *Oryzias latipes* , the sequence divergence between sex chromosomes is relatively low, with no significant gene loss or sequence degradation (Matsuda et al. 2002). A possible explanation for the relatively low degeneration in *B*. *sangzhiensis* could be the occurrence of occasional sex reversal events, which facilitate recombination between sex chromosomes and thereby prevent their degeneration (Rodrigues et al. 2018). These findings suggest that the sex chromosomes of *B*. *sangzhiensis* are still in a relatively early stage of divergence. Copy number analysis (Section 3.5) further supports the early‐divergence status of these sex chromosomes as the initial stages of sex chromosome evolution are driven by massive amplification of distinct gene classes (Bachtrog et al. 2019).

### 3.3 Sex‐Linked Genetic Differentiation, Y‐Chromosome Haplotypes, and Coexistence of Dual SLRs

**FIGURE 3:**
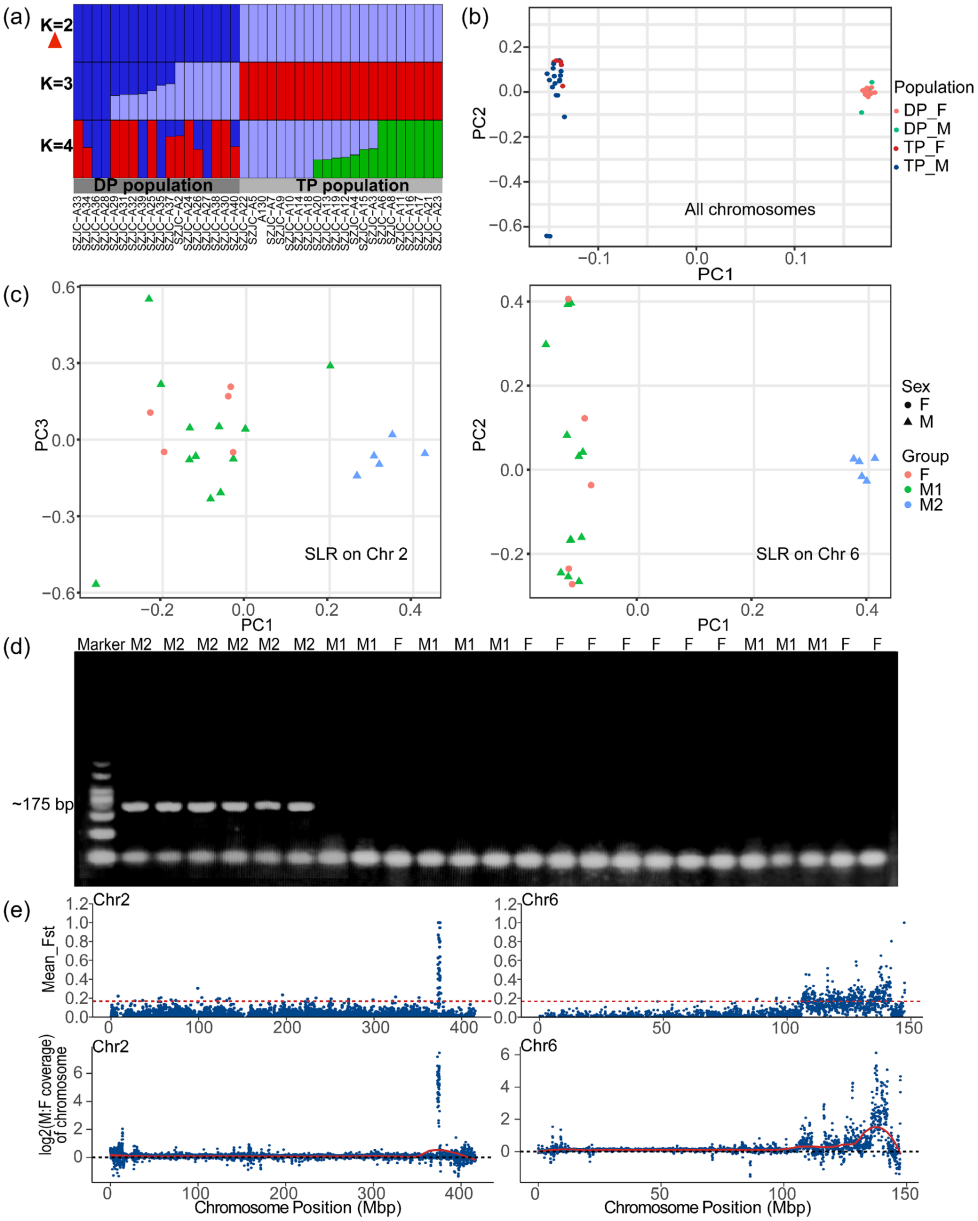
(a) STRUCTURE analysis based on genome‐wide SNPs (LD‐pruned) from 20 males and 20 females. *K* = 2 is the optimal value, separating samples by TP and DP population. (b) PCA of genome‐wide SNPs shows the similar two clustering. (c) PCA plots for SNPs in SLRs on Chr 2 (left) and Chr 6 (right) in the TP population. Individuals are coloured by group: Females (F, red), M1 (green), and M2 (blue). (d) PCR validation of the male‐specific marker. Marker: 500 bp. The expected amplicon (~175 bp) was detected only in M2, whereas females (F) and the M1 showed only the shared band. The shared faint band at ~50 bp present in all lanes likely represents primer dimers or a non‐specific short product. (e) Visualization of *F*_ST_ values based on SNPs (top) and log_2_(M:F) coverage ratios (bottom) on Chr 2 (left) and Chr 6 (right) from 5 females and 5 males (M2) in TP population (50 Kb windows). The dashed red line in *F*_ST_ panel indicates the threshold (the top 1% cutoff) for identifying regions with significant differentiation between males and females.

*B*. *sangzhiensis* exhibits geographic variation in sex‐linked differentiation. Genome‐wide STRUCTURE and PCA analyses separated the TP and DP populations (Figures 3a,b and S12a). In the TP population, and similarly in the DP population (Figure S12b), PCA based on SNPs separated phenotypic males into two genotypic classes (M1 and M2), with M1 clustering with females and M2 forming a distinct male‐like cluster (Figure 3c). Thus, in these populations, phenotypic males do not constitute a single homogeneous genetic group, but instead comprise one class that is largely female‐like (M1) and another that is more strongly male‐associated (M2). This pattern indicates incomplete concordance between phenotypic sex and sex‐linked genomic differentiation, an issue examined further below. Because male sampling in the DP was very limited, the detailed analyses below focus on the TP, whereas the DP is treated as supportive evidence.

To validate male‐specific signal independently of the PCR assay, we defined the more strongly male‐associated genotypic class (M2) using resequencing‐based analyses (PCA; Figure 3c), and then designed primers from the Chr 2 male‐specific coverage region as an independent validation and screening marker. PCR consistently amplified the expected male‐specific fragment only in M2 individuals (Figure 3d). Sanger sequencing further confirmed that the amplified fragment mapped to the Chr 2 male‐specific coverage region (Figures S13 and S14). These results support the presence of a male‐specific sequence within the Chr 2 SLR and identify M2 as the more strongly male‐associated, or Y‐like, genotypic class in our data. However, because our samples were collected from wild individuals and no pedigree‐ or trio‐based data are available, these results do not permit definitive inference about causality or physical linkage. The male‐specific sequence within the Chr 2 SLR therefore warrants further investigation to clarify its role in sex‐associated variation.

Within the TP population, M1 and M2 represent markedly different male‐associated states. Male‐specific SNPs were abundant in M2 but essentially absent in M1, while female‐specific SNPs were not detected (Table S14). Site‐based scans further showed that M2 showed extensive differentiation from females, whereas M1 retained only weak but detectable residual differentiation, predominantly on Chr 6 (Table S14). This interpretation was consistent with the weak *F*_ST_ between females and M1 (Figure S15), the very low overlap between M1‐ and M2‐associated loci (Table S14), genotype heatmaps showing distinct loci between females and M1 (Figure S16a) and the NJ tree based on informative Chr 6 loci (Figure S16b), which placed M1 between the female cluster and the strongly male‐associated M2 cluster. These results indicate that M1 and M2 may carry distinct Y‐chromosome haplotypes, with M1 being compatible with less divergent Y haplotypes, potentially associated with X‐Y recombination (Carpentier et al. 2026).

To investigate whether the sex‐linked signals on Chr 2 and Chr 6 co‐occur within individuals, we focused on the TP population, where the two male genotypic classes provide an informative contrast (M1, *n* = 12; M2, *n* = 5; Figure 3c). Each male class was contrasted against the same set of 5 females, generating FM1 and FM2 comparison groups. In FM2, analyses of *F*_ST_, sex‐related SNP, log_2_(M:F) coverage ratios, LD, and genotype patterns revealed significant signals on both Chr 2 and Chr 6, consistent with previously identified SLRs (Figures 3e and S17–S19). Whereas FM1 group showed only very weak differentiation at the candidate loci on both Chr 2 and Chr 6 (Figure S15). We further performed a genotype‐based scan for XY‐like or male‐specific loci and calculated per‐individual sex‐linkage scores for the Chr 6 and Chr 2 SLRs. This analysis identified abundant XY‐like or male‐specific loci within the Chr 6 SLR but comparatively few within the Chr 2 SLR. Despite this asymmetry, per‐individual sex‐linkage scores showed strong concordance between Chr 2 and Chr 6 (Table S14 and Figure S20). These analyses support non‐random, concordant occurrence of Chr 2‐ and Chr 6‐associated sex‐linked signatures within individuals in TP, particularly in M2. A qualitatively similar pattern was observed in DP (Figure S21 and Table S14), but inference for that population is limited by the very small male sample size.

The concordant occurrence of sex‐linked signatures on Chr 2 and Chr 6 in *B. sangzhiensis* is reminiscent of multi‐chromosome sex‐determination systems reported in *R. temporaria* (Rodrigues et al. 2016; Toups et al. 2019) and is compatible with, but does not by itself demonstrate, a potentially polygenic sex determination (PSD) architecture in which sexual phenotype is influenced by multiple loci (Roberts et al. 2016; Moore et al. 2022; Schartl et al. 2023). Such concordance could arise through chromosomal rearrangements that couple inheritance across chromosomes, such as reciprocal translocations (Toups et al. 2019), or through long‐range, potentially inter‐chromosomal LD generated or maintained by epistatic selection in a multi‐locus system (Slatkin 2008; Ross et al. 2011; Hench et al. 2019). However, our data do not distinguish PSD from other mechanisms that could generate concordant sex‐linked signatures across chromosomes. Moreover, documented cases of PSD remain rare and are often interpreted as transitional states during sex chromosome turnover or as aberrant situations in hybrids between taxa with different sex chromosomes (Schartl et al. 2023).

### 3.4 Candidate SDGs in the SLRs

#### 3.4.1 Candidate SDGs in the SLR on Chr 2

**FIGURE 4:**
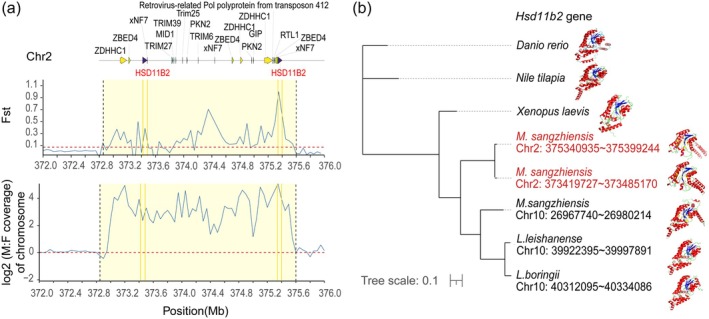
(a) A zoomed‐in region (372.8–375.6 Mb, window = 50 kb) between the two vertical dashed lines is highlighted and represents the SLR. The solid dark yellow line indicates the position of the *Hsd11b2* gene. In the *F*_ST_ track, the dashed red line indicates the threshold (the top 1% cutoff) for identifying regions with significant differentiation between males and females. In the log2(M:F) coverage track, the horizontal dashed red line marks log2(M:F) = 0, corresponding to equal male and female read depth. (b) Maximum likelihood phylogenetic tree and protein structure characterize of *Hsd11b2*. The tree shows the evolutionary relationships among *Danio rerio* , *Nile tilapia*, *Xenopus laevis* , and several *B*. *sangzhiensis* populations, as well as closely related species *L. leishanensis* and *L. boringii* . The highlighted *Hsd11b2* genes on Chr 2 in *B*. *sangzhiensis* represent candidate SDGs. Protein structures corresponding to the gene in different species are shown on the right, highlighting the conservation and divergence of sequences across species. Blue represents strands (beta sheets) regions, red represents alpha helices regions, yellow represents NADP binding sites, and purple represents steroid binding sites.

In the small SLR on Chr 2, we identified 21 annotated genes, with *Hsd11b2* located near the largest *F*_ST_ and log_2_(M:F) coverage ratio peak (Figure 4a). Notably, *Hsd11b2* belongs to the hydroxysteroid deoxygenase family (HSD), which is also closely linked to sex differentiation and gonadal development. Within the HSD family, *Hsd17b1* is considered a key sex determination/differentiation gene (Guo et al. 2024; Wei et al. 2023). In *Seriola* species and *Trachinotus anak* , *Hsd17b1* has been identified as a sex‐determining master control regulator (SD MKR) (Curzon et al. 2023). In zebrafish, knockout of *Hsd17b1* resulted in homozygous *Hsd17b1*‐deficient individuals that were exclusively male, supporting the role of *Hsd17b1* in phenotypic sex determination (Guo et al. 2024). Guo et al. suggested that, in *Seriola* species, the combination of *Hsd17b1* alleles determines sex by regulating endogenous oestrogen levels (Guo et al. 2024). Based on these observations, we hypothesize that *Hsd11b2*, which is involved in steroid hormones biosynthesis, may also play a crucial role in sex determination in *B*. *sangzhiensis*.

Interestingly, *Hsd11b2* gene has undergone multiple duplications in *B*. *sangzhiensis*, particularly on Chr 2, where two copies of the gene are located in the sex‐related region. In contrast, closely related species within the same family, such as *L. boringii* and *L. leishanensis* , possess only a single copy of *Hsd11b2* on Chr 10. Although the *Hsd11b2* gene in *B*. *sangzhiensis* can also be found on Chr 10, the additional copies on Chr 2 are unique to this species. Phylogenetic analysis and multiple sequence alignments reveal that the *Hsd11b2* gene on Chr 10 is relatively conserved, whereas the copies on Chr 2 are distinct and specific to *B. sangzhiensis* (Figure S22). This specific duplication (Matsuda et al. 2002; Hattori et al. 2012; Yano et al. 2012) and variation (Koyama et al. 2019) make *Hsd11b2* a putative MSD. Protein structure analysis using AlphaFold3 further supports the functional significance of these duplications. The NADP‐binding region of *Hsd11b2* is conserved across species; however, the steroid‐binding region in the sex‐linked copies on Chr 2 in *B. sangzhiensis* exhibits variations (Figure 4b). These variations are likely to influence the enzyme's activity, specifically its ability to catalyse the conversion of estrone to estradiol, a key step in sex differentiation. This may contribute to the development of binary sexual phenotypes (Koyama et al. 2019). Further investigations of the X‐derived and Y‐derived protein sequences of *Hsd11b2* are warranted.

In addition, *Hsd11b2* expression in male gonad tissues of *B*. *sangzhiensis* was significantly upregulated compared to females (Figure S23). In teleost fish, *Hsd11b2*, highlighted as a key mediator linking environmental cues (e.g., temperature stress) to gonadal sex outcomes, inactivates cortisol (the main stress hormone) and helps produce 11‐oxygenated androgens like 11‐ketotestosterone (Shen and Wang 2018). *Hsd11b2* is strongly upregulated under masculinizing conditions (temperatures or cortisol treatment) during the critical sex‐determining period (Fernandino et al. 2013; Valdivieso et al. 2023). In situ hybridization localizes *Hsd11b2* expression to Leydig cells of the gonad (Fernandino et al. 2012; Kusakabe et al. 2003). Elevated temperature or cortisol exposure increases *Hsd11b2* expression and 11‐ketotestosterone levels, skewing sex ratios towards males (Shen and Wang 2018). *Hsd11b2* thence may be a strong candidate gene involved in stress‐ or temperature‐induced masculinization and sex determination. However, whether the upregulation of *Hsd11b2* is directly triggered by temperature or indirectly mediated through cortisol remains to be determined. Notably, the localization of *Hsd11b2* within the SLR on Chr 2 may suggest a potential link between stress‐responsive endocrine regulation and sex determination in *B. sangzhiensis*. Further functional validation will be required to confirm its involvement in sex determination in *B. sangzhiensis*.

#### 3.4.2 Candidate SDGs in the SLR on Chr 6

Within the SLR on Chr 6, a total of 726 genes were annotated. Consistent with zebrafish, this region is predominantly composed of genes from the zinc finger protein (ZNP) family, NOD‐like receptor (NLR) family and tripartite motif‐containing (TRIM) family (Table S17) (Wilson and Postlethwait 2024; Fraser et al. 2020). Enrichment analysis of KEGG terms of these genes were enriched in NOD‐like receptor signalling pathway (*q*‐value = 5.66 × 10^−5^), RIG‐I‐like receptor signalling pathway (*q*‐value = 1.74 × 10^−4^), Necroptosis (*q*‐value = 2.10 × 10^−4^), and NF‐kappa B signalling pathway (*q*‐value = 2.65 × 10^−3^) (Table S18). By specifically searching for members of the gene families that have been implicated in sex determination (e.g., *DMRT1*, *DM‐W*, *SOX9*, *CYP17*, *FOXL2*, *AMH*, *TGF‐β*) (Kikuchi and Hamaguchi 2013; Jeffries et al. 2018; Han et al. 2022), we did not identify any members in the SLR. However, a *CYP17A1* ortholog, homologous to *CYP17*, was found in a non‐sex‐linked region of Chr 6 (28.31–28.33 Mb). These findings suggest that the candidate SLR of *B. sangzhiensis* may harbour novel SDGs distinct from those previously reported.

Interestingly, we discovered the *Nlrp14* gene in the SLR on Chr 6, a member of the NLRP family, which, unlike other NLRP genes, was specifically expressed in the gonads and involved in regulating germ cell differentiation (Westerveld et al. 2006; Abe et al. 2017; Davis et al. 2018). *Nlrp14* deficiency in mice resulted in reduced differentiation of primordial germ cell‐like cells (PGCLCs) in vitro and reproductive failure in both male and female in vivo. In male mice, *Nlrp14* knockout significantly compromised differentiation of spermatogonial stem cells and meiosis (Yin et al. 2020). In *B*. *sangzhiensis*, *Nlrp14* also exhibited three copies and displayed significantly male‐biased expression, consistent with findings in both mice and humans (Figure S24). In zebrafish, NLR genes in the SLR are believed to be downregulated and unnecessary or deleterious in ovaries (Wilson and Postlethwait 2024). Therefore, in *B*. *sangzhiensis*, *Nlrp14* may play a crucial role in sex determination or sex differentiation by triggering the male pathway and repressing the female pathway.

Genes located within the candidate SLR were predominantly zinc finger (ZNF) genes (approximately 80%), as shown in Table S17. This is consistent with observations in zebrafish (Wilson and Postlethwait 2024). We suggested, in agreement with previous studies, that members of ZNF family are likely to play an essential role in sex determination or regulation (Wilson and Postlethwait 2024; Han et al. 2022; Fraser et al. 2020; Yang et al. 2021; Nakasuji et al. 2017). For instance, *ZNF226L* has been proposed as the candidate SDGs (Han et al. 2022) and *ZNF706* was evidenced that regulated germ plasm assembly and primordial germ cell development (Zhang et al. 2024). Furthermore, Y‐linked zinc finger protein (ZFY) gene have been found to be strongly associated with sex determination in previous studies. In silkworms, *Znf‐2* is crucial for male sex differentiation, as it controls male‐specific splicing of the primary sex determiner doublesex (*dsx*) gene. Mutant males lacking *Znf‐2* exhibit feminized external genitalia (Yang et al. 2021). Double knockout (*Zfy1/2*‐DKO) mice are infertile and exhibit abnormal sperm morphology, fertilization failure, and early embryo development failure (Nakasuji et al. 2017). Therefore, multi‐copy *ZNF* genes (e.g., *XlCGF66.1*, *ZFP250*, *ZFP3*, *ZNF667*, *ZFP30*), exhibiting significant sex differentiation and biased male:female coverage (Figure S25), may play a potential role in sex determination in *B*. *sangzhiensis*. However, further studies are needed to provide evidence for a possible role of these genes in sex determination in *B*. *sangzhiensis*.

### 3.5 Driver of the Formation of Sex Chromosome in *B. sangzhiensis*

**FIGURE 5:**
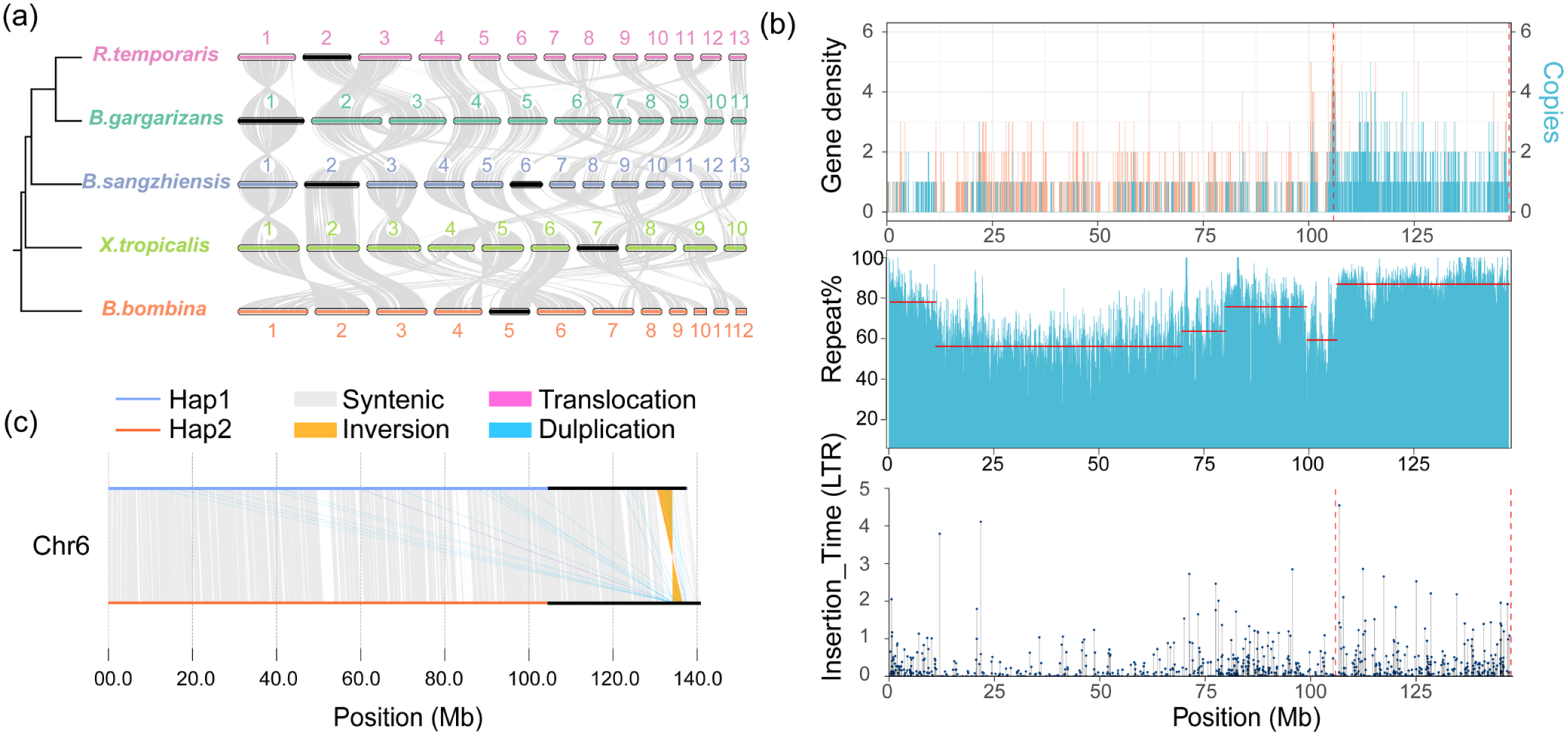
The syntenic relationships between *B. sangzhiensis*, *R. temporaria*, *X. tropicalis* and *B. gargarizans* and *B. bombina* are shown in (a). The chromosomes of each species are labelled, with sex chromosomes highlighted in black. Phylogenetic tree on the left was obtained from TIMETREE97 (http://timetree.org). Characterization of Chr 6 in *B*. *sangzhiensis* is shown in (b). Sex related region is indicated by red vertical dashed lines. The first track shows the gene density (yellow lines) and multi‐copy gene distribution (blue lines) across Chr 6, calculated in 50 kb sliding windows. Homologous genes were identified using OrthoFinder software, and multi‐copy genes were selected from these. The second track shows the change‐point analysis of repetitive sequences, with a 50 kb sliding window. Each horizontal line represents the mean repeat content% of a segment. The change‐point analysis was conducted using the command: cpt.mean (method = ‘BinSeg’) (Killick and Eckley 2014). The final track shows the insertion time of LTRs, which are the mostly abundant repeat contents. Chromosomal structural variants between Hap1 and Hap2 of Chr 6 in *B. sangzhiensis* are shown in (c). The black regions indicate candidate SLRs.

Whole‐genome macrosyntenic relationships showed that Chr 2 of *B*. *sangzhiensis* is orthologous to the sex chromosome 2 of *R. temporaria* in Ammarnäs (Toups et al. 2019), while Chr 6 of *B*. *sangzhiensis* is orthologous to the sex chromosome (Chr 7) and a small part of Chr 9 of *X. tropicalis* (Figure 5a) (Bewick et al. 2013; Furman, Cauret, et al. 2020). Interesting, a shared collinearity region spanning 0.4–105.7 Mb was identified between *B*. *sangzhiensis* and *X. tropicalis* , but this region did not overlap with SLR (Figure 5a). In order to better understand the formation of sex chromosome and SLR, we performed chromosomal rearrangement analysis between *B. sangzhiensis*, close relatives of *B. sangzhiensis* and species with collinear sex chromosomes. We identified multiple chromosomal rearrangements (inversions and duplications) both on Chr 2 and Chr 6 (Figures S26–S28). Notably, synteny with Chr 7 of *X. tropicalis* highlights repeated recruitment of sex chromosome and an expansion of the SLRs on Chr 6 (Figures 5a and S26). This finding suggests that homology alone does not restrict genomic regions from becoming sex‐linked (Vicoso and Bachtrog 2015; Gammerdinger and Kocher 2018; Jeffries et al. 2018; Cauret et al. 2020; Meisel et al. 2020). Similar differences in candidate SLRs have also been reported in fishes (Jasonowicz et al. 2022). These observations are consistent with the rapid turnover of sex chromosomes and may be driven by allelic diversification or gene duplication and translocation events associated with the emergence of novel SDGs (Herpin et al. 2010; Miura 2008; Furman, Metzger, et al. 2020; Bertho et al. 2021; Volff et al. 2007; Tanaka et al. 2007; Ross et al. 2009; Veltsos et al. 2019; Herpin et al. 2021; Wang et al. 2022).

The SLR on Chr 6 displays extensive intragenomic collinearity, with duplicated blocks within the same chromosome. Self‐alignment dotplots reveal clusters of muti copy genes with strong sequence similarity along Chr 6 (Figures 1a and 5b). This pattern reflects recent intragenomic duplications. Consistent with this interpretation, the SLR shows a high density of multi‐copy genes, abundant repetitive elements, and numerous young LTR insertions (Figure 5b), together with significant TE enrichment (Figure S29). These features indicate recent duplication and TE–mediated rearrangements. Local inversions and translocations detected in this region further support chromosomal rearrangements (Figures 5c and S26–S28). Retrotransposons can mobilize gene fragments via mRNA intermediates, leading to gene duplication and translocation, thereby generating raw material for new gene or regulatory element evolution (Kazazian 2004; Kaessmann 2010). The high copy numbers and sequence similarities of retrotransposons (e.g., LTR, LINE, SINE) can also promote non‐allelic homologous recombination and segmental duplication generating rearrangements that locally suppress recombination (Hua‐Van et al. 2005; Furman, Metzger, et al. 2020; Gray 2000). Such processes may drive the accumulation of sex‐specific elements and the expansion of non‐recombining regions on sex chromosomes (Lenormand and Roze 2022; Wright et al. 2016; Jasonowicz et al. 2022; Furman, Metzger, et al. 2020). In support of this hypothesis, our analysis of LD (Figure S18c) shows higher LD within SLR, suggesting reduced recombination in males. These results suggest that TE‐driven duplications and rearrangements have shaped the structure and differentiation of the Chr 6 sex‐linked region in *B. sangzhiensis*.

Overall, our findings suggest that TE‐mediated chromosomal rearrangements (e.g., segmental duplications, inversions) may have facilitated the formation and expansion of the SLR on Chr 6 in *B. sangzhiensis* (Zheng et al. 2025; Hua‐Van et al. 2005; Furman, Metzger, et al. 2020; Gray 2000).

## 4 Conclusion

In this study, we successfully assembled a high‐quality chromosome‐level genome of *B*. *sangzhiensis*, providing a foundational genomic resource for understanding the evolution of homomorphic sex chromosomes. Our analyses yielded several key findings: (1) identification of candidate sex‐linked regions (SLRs) on Chr 2 and Chr 6, characterized by male‐specific coverage and elevated *F*_ST_ signals; (2) identification of an XY system in *B. sangzhiensis*; (3) discovery of a Y‐specific sequence within the candidate SLR on Chr 2, validated through PCR and Sanger sequencing; (4) identification of sex‐determining candidate genes (e.g., *Hsd11b2, Nlrp14*, and zinc finger genes) with multiple copies showing elevated log_2_(M:F) coverage ratios and higher *F*_ST_ values; (5) observation of multiple Y haplotypes in the TP population; (6) evidence of TE accumulation, gene duplications, and chromosome inversion contributing to early sex chromosome differentiation. Overall, these findings suggest that *B*. *sangzhiensis* may be a good model for exploring the early stages of sex chromosome evolution. While our data support two candidate sex‐linked regions on Chr 2 and Chr 6 and their concordant association across individuals, the genetic mechanism and alternative explanations linking these signals remains unresolved and will require pedigree‐based and structural validation. Future studies could focus on functional validation of candidate sex‐determining loci and comparative epigenomic analyses to unravel environmental interactions with conserved regulatory networks. Our study establishes a critical framework for redefining the evolutionary trajectories of sex chromosomes in non‐model vertebrates.

## Author Contributions

Chun H. Huang: collecting sample, writing‐original draft, visualization, formal analysis, conceptualization. Chun H. Huang and Wan Y. Chen: species and gender identification. Fu Y. Qiu: transcriptome data provision. Si Y. Xie, J. Li, and H. Wu: writing‐review and editing. H. Wu: designing the original concept and scientific objective, funding acquisition and supervision.

## Funding

This work was supported by The National Natural Science Foundation of China (32071488).

## Disclosure

Benefit‐sharing statement: Benefits from this research accrue from the sharing of our data and results on public databases.

## Conflicts of Interest

The authors declare no conflicts of interest.

## Supporting information


**Figure S1:** Geographic location of the sampling sites of *B. sangzhiensis* in the Badagong Mountain National Nature Reserve, Hunan Province, China. The right panel shows two specific collection sites: Tiangping Mountain and Doupeng Mountain within the Sangzhi county These localities represent the distribution range of *B. sangzhiensis* used for genomic and transcriptomic sampling.
**Figure S2:** Gonadal tissues of sexually mature male (a) and subadult female individuals at different developmental stages (b, c). T indicates the testes, and O indicates the ovaries. All individuals were derived from the resequencing dataset used in this study.
**Figure S3:** The chromosome (a) and whole‐genome (b) Hi‐C interaction maps of *B. sangzhiensis* at 500 kb resolution. The colour ranging from light to dark indicate increasing contact frequency between genomic loci, with darker shades indicating stronger interactions. Hi‐C contact maps showed 13 strong interaction blocks corresponding to the 13 chromosomes of *B. sangzhiensis*.
**Figure S4:** Whole‐genome macrosyntenic relationships between *Boulenophrys sangzhiensis* and its closely related species ( *L. tengchongense* , *L. promustache* , *L. guangxiense* , *L. leishanense* , *L. liui* , and *L. boringii* ). Each line connects orthologous genomic regions between homologous chromosomes. Chr 6 of *B. sangzhiensis* shows a clear one‐to‐one correspondence with Chr 6 of its relatives, with no evidence of interchromosomal fusion or translocation, supporting the structural integrity of the *B. sangzhiensis* assembly.
**Figure S5:** Chromosome anchoring and assembly integrity of *B. sangzhiensis* haplotypes. (a) Hi‐C contact maps of haplotype 1 (Hap1) and haplotype 2 (Hap2) at 1‐Mb resolution. The dense and continuous diagonal signals indicate high assembly continuity and accurate chromosome anchoring. (b) Distribution of contigs across chromosomes for Hap1 and Hap2. Grey bars represent chromosome length, while coloured blocks represent contigs of different size ranges (5 Mb). Most chromosomes are anchored by a few large contigs, confirming high contiguity of the assembly.
**Figure S6:** Validation of the assembly integrity on chromosome 6 in *B. sangzhiensis*. (a) Hi‐C contact heatmaps of chromosome 6 for haplotype 1 (Hap1) and haplotype 2 (Hap2). The *x*‐ and *y*‐axes represent chromosomal coordinates. (b) Local magnification of the Hi‐C contact maps showing continuous contact patterns near the putative breakpoints. Juicebox visualization confirmed that chromosome 6 is represented by a single contig, with contact breakpoints located at ~103.2 Mb on Hap1 and ~104.7 Mb on Hap2. (c) Mapping depth and read alignment patterns near the corresponding breakpoint regions. PacBio HiFi reads were mapped to the merged haplotype assembly using Minimap2 (v2.24, ‐axe map‐hifi). The presence of numerous reads spanning the breakpoint regions indicates the absence of assembly gaps or structural misjoins.
**Figure S7:** Chromosomal collinearity and structural variation between the two pseudo haplotypes (Hap1 and Hap2) of *B. sangzhiensis*. Syntenic relationships and large‐scale structural variants were identified by whole‐genome alignment using Syri. Hap1 and Hap2 assemblies are shown in blue and orange, respectively. Grey lines indicate collinear (syntenic) regions, while structural rearrangements are highlighted as follows: orange lines represent inversions, green lines represent translocations, and cyan lines represent duplications. Extensive collinearity is observed across most chromosomes, with several localized rearrangements detected—particularly on Chr 6, suggesting potential intra‐chromosomal duplication and inversion events within the sex‐linked region.
**Figure S8:** Change‐point analysis of GC content of *B. sangzhiensis*. Each horizontal line represents the mean GC% (window = 50 kb) of a segment identified by change‐point analysis (Killick and Eckley 2014). Commands for change‐point analysis was cpt.mean(method = ‘BinSeg’). We can observed that GC content varied between 40% and 60% across chromosomes, with the sex‐associated region on Chr 2 and Chr 6 showing moderate GC content (40%–50%), rather than the highest GC levels genome‐wide.
**Figure S9:** Genome‐wide identification of sex‐linked regions in *B. sangzhiensis* (window = 50 kb). (Top) Genome‐wide distribution of Indel‐based *F*_ST_ values between 20 males and 20 females across all chromosomes. Orange and blue dots represent InDels with positive and negative *F*_ST_ values, respectively. (Bottom) Detailed analysis of Chr 2 and Chr 6. For each chromosome, plots show the log_2_(M:F) InDel density (top panels) and Indel‐based *F*_ST_ values (bottom panels) calculated in non‐overlapping windows.
**Figure S10:** Heat map of linkage disequilibrium (LD) in SLR on Chr 2 from 20 males (a) and 20 females (b), showing high *R* ^2^ values. (c) LD decay analysis showing the relationship between distance (kb) and LD (*R* ^2^) on Chr 6 in 20 males (a) and 20 females. Different colours represent male and female chromosomes, highlighting sex‐specific LD decay patterns. LD patterns in *B. sangzhiensis* based on variant data from 20 females and 20 males.
**Figure S11:** Integrative Genomics Viewer (IGV) screenshot showing the sex‐specific coverage bias in the putative sex‐linked region on Chr 6 of *B. sangzhiensis*. The coverage tracks display for both males and females the read depth (scale 0–50 reads). This region of chromosome 6 corresponds to 131,146,590‐131,172,415 bp. Red, green and blue track represents female, M1 and M2, respectively.
**Figure S12:** (a) PCA plot for SNPs in sex‐linked region on Chr 2 and Chr 6 in two populations: Tianping Mountain (TP) and Doupeng Mountain (DP). (b) PCA plot for SNPs in sex‐linked region on Chr 2 and Chr 6 in DP population. (c) PC1–PC2 plot for SNPs in sex‐linked region on Chr 2 in TP population.
**Figure S13:** Mapping of Sanger sequencing PCR product to the genome region using Geneious software. The image shows the alignment of Sanger sequencing data (bottom) to the reference genome (top) within the Geneious interface. The green bars represent the aligned regions, with coverage indicated by the blue shading. This visualization demonstrates the successful mapping of the PCR product to the target genomic region, providing confirmation of sequence identity and quality. The alignment also shows the detailed nucleotide sequence at the mapped location.
**Figure S14:** Visualization of the sex ‐specific coverage in *B. sangzhiensis* using IGV. 12 individuals were randomly selected. Blue indicating male phenotypic sex and red indicating female phenotypic sex. Dark blue represents normal males, whose genotype and phenotype are both male, showing coverage in these regions. Light blue‐green represents sex‐reversed individuals, which, similar to red‐coloured genotypic females, show no coverage.
**Figure S15:** Genomic differentiation between M1 (12 males) and 5 females (a) and M1 and M2 (b) in *B. sangzhiensis* from the Tianping Mountain population. Each panel shows the mean *F*_ST_ based on SNP, log2(M:F) coverage ratio, and SNP density calculated in 50‐kb sliding windows. The dashed red line marks the top 1% *F*_ST_ threshold used to define regions of significant genetic differentiation between groups. Peaks in *F*_ST_, male‐biased coverage, and male‐biased SNP density on Chr 2 and Chr 6 highlight putative sex‐linked regions.
**Figure S16:** (a) Genotype heatmap based on filtered informative loci from the top strict differentiated loci in the F vs. M1 comparison within the Chr 6 SLR of *B. sangzhiensis* from the TP population. Individuals are grouped as females (F), M1 males, and M2 males. Genotypes are coded as 0/0, 0/1, and 1/1, with missing data shown in grey. (b) Neighbour‐joining (NJ) tree was construct based on the F vs. M2 relaxed loci and F vs. M1 strict loci Chr 6 in SLR on Chr 6 using ape package. M2 forms the most strongly differentiated male‐like cluster, females form a separate cluster, and M1 occupies an intermediate position. These results are consistent with weak but detectable residual Chr 6‐linked differentiation in M1 males.
**Figure S17:** Sex‐linked signal detection on Chr 2 and Chr 6 in *B. sangzhiensis* (5 M2 males vs. 5 females) from the Tianping Mountain population (window size = 50 kb). Upper panels: log₂(M:F) ratio of SNP density across Chr 2 and Chr 6, showing sex‐biased SNP distribution. Lower panels: number of sex‐related SNPs along Chr 2 and Chr 6.
**Figure S18:** Heat map of linkage disequilibrium (LD) in SLR on Chr 2 in 5 males (a) and 5 females (b), showing high *R* ^2^ values. (c) LD decay analysis showing the relationship between distance (kb) and LD (*R* ^2^) on Chr 6. Different colours represent male and female chromosomes, highlighting sex‐specific LD decay patterns. LD patterns in *B. sangzhiensis* based on variant data from 5 females and 5 males (M2) in Tianping Mountain.
**Figure S19:** Genotype heatmap of the sex‐linked region on chromosome 6 in *B. sangzhiensis*, based on five female and five male individuals (M2). Each column represents an individual, and each row represents a SNP within the sex‐linked region. Colours indicate genotypes: homozygous reference (0/0, blue), heterozygous (0/1, yellow), and homozygous alternate (1/1, red). The consistent genotype segregation between sexes highlights a strongly sex‐associated haplotype block on chromosome 6.
**Figure S20:** Individual‐level concordance between sex‐linkage signals on Chr 6 and Chr 2 in the TP population. Each point represents one TP individual (5 females, 12 M1 males, 5 M2 males). XY‐like loci were defined a priori using the F vs. M2 contrast (5 females vs. 5 M2 males) as sites where all called females were fixed for the same homozygous genotype (all 0/0 or all 1/1) together with enrichment of male heterozygosity (0/1), a criterion invariant to REF/ALT assignment. Using this fixed marker panel (Chr 6: 70,924 loci; Chr 2: 26 loci), we computed per individual sex‐linkage scores as the proportion of heterozygous genotypes (0/1) across XY‐like loci separately for Chr 6 (*x*‐axis) and Chr 2 (*y*‐axis). Dashed lines indicate the predefined thresholds used to dichotomize scores for the co‐occurrence test (Chr6 ≥ 0.6; Chr2 ≥ 0.3), separating individuals into ‘Chr6‐high/Chr2‐high’ and ‘Chr6‐low/Chr2‐low’ quadrants. Scores clearly separated females (Chr6 mean = 0; Chr2 mean = 0), M1 males (Chr6 mean = 0.046; Chr2 mean = 0.039), and M2 males (Chr6 mean = 0.897; Chr2 mean = 0.785). M2 males cluster in the Chr6‐high/Chr2‐high quadrant, whereas females and M1 males cluster in the Chr6‐low/Chr2‐low quadrant, consistent with strong individual‐level concordance of male‐like signatures between Chr 6 and Chr 2 in TP (Spearman's *ρ* = 0.725; *p* = 1.37 × 10⁻⁴; Fisher's exact test *p* = 3.80 × 10⁻⁵; no discordant individuals). DP results are not shown here because male sampling was limited (3 males; M2 *n* = 1).
**Figure S21:** Genomic differentiation between all males and 5 females (a), M2 and 5 females (b), M1 and 5 females (c) and M1 and M2 (d) in the DP population. Each panel shows the mean *F*_ST_ based on SNP and log2(M:F) coverage ratio calculated in 50‐kb windows. The dashed red line marks the top 1% *F*_ST_ threshold used to define regions of significant genetic differentiation between groups.
**Figure S22:** Phylogenetic analysis and multiple sequence alignments of *Hsd11b2* gene in different species. The top panel displays the consensus sequence with corresponding hydrophobicity values represented by a colour gradient. The coloured bars beneath the consensus line indicate the hydrophobicity levels of individual amino acids across the sequence, with red representing hydrophobic residues, blue for hydrophilic residues, and green for neutral residues. This figure was used to compare *Hsd11b2* sequences within the sex‐linked region of *B. sangzhiensis*, showing that although the gene is highly conserved, sequence differences exist among species and between gene copies within the same genome.
**Figure S23:** The expression level of *Hsd11b2* genes on Chr 2 in *B. sangzhiensis*. (a) Overall expression levels of *Hsd11b2* in males and females, showing significantly higher expression in males (*p* < 0.001) compared to females. (b) Expression levels of two *Hsd11b2* paralogs, MPa0013900 and MPa0014090, both exhibiting male‐biased expression (*p* < 0.01, *p* < 0.05, respectively).
**Figure S24:** Characterize of *Nlrp14* gene in *B. sangzhiensis*. (a) sex‐linked region harboured three copies of *Nlrp14* gene. (b) The expression level of *Nlrp14* genes. We could find that all three copies showed male‐biased expression. (c) The gene structure of *Nlrp14*. SNP, single nucleotide polymorphism; CDS, coding sequences; UTR, untranslated region.
**Figure S25:** Characterize of candidate ZNF genes on Chr 6. The top track shows the mean *F*_ST_ values across Chr 6 for 5 females and 5 males from Tianping Mountain, the red and grey horizontal lines represent the top 1% cutoff for significant divergence at the entire chromosome level and at Chr 6 between females and males, respectively. The middle track shows the male‐to‐female coverage ratio, calculated as log2 (male coverage +0.01) ‐ log2 (female coverage +0.01). The pink vertical lines represent the distribution of the corresponding candidate *ZNF* gene. The bottom panel illustrates the distribution of differentially expressed genes (DEGs) along Chr 6 based on log₂ fold change values. Red points denote DEGs located within the sex‐linked region, while grey points represent DEGs in non–sex‐linked regions. Negative log₂ fold change values correspond to male‐biased genes, and positive values indicate female‐biased genes. The vertical pink lines mark the genomic positions of candidate *ZNF* genes (*ZNF667*, *ZFP30*, *ZFP3*, *ZFP250*, and *XlCGF66.1*), which are strongly associated with sex‐linked differentiation.
**Figure S26:** Chromosomal structural variants of *B. sangzhiensis*, *X. tropicalis* and *L. leishanense* . Black region on chromosome was the sex‐linked region. left is the phylogenetic tree of the three species, with red numbers representing divergence times.
**Figure S27:** Chromosomal structural variants of *B. sangzhiensis*, *R. temporaria* and *L. leishanense* . Black region on chromosome was the sex‐linked region.
**Figure S28:** Chromosomal structural variants among *R. temporaria* , *B. sangzhiensis*, and *X. tropicalis* . Blue, orange, and green bars represent chromosomes from the three species, respectively. Grey ribbons indicate syntenic regions, while orange ribbons denote inversion events. Multiple inversions are observed between *R. temporaria* and *B. sangzhiensis*, whereas *X. tropicalis* shows lower overall synteny with the other species.

**Table S1:** Sample information.
**Table S2:** Species identification result of resequencing sample.
**Table S3:** The alignment statistics of the resequencing sample sequences against the genome of *B. sangzhiensis*.
**Table S4:** Library data statistics of SMRT sequencing by using the PacBio sequencing platform.
**Table S5:** Assembly result of HiFiasm.
**Table S6:** Mapping statistics of short‐read and PacBio HiFi read alignments to the assembled genome.
**Table S7:** Statistics on homozygous rate and heterozygous rate.
**Table S8:** *B. sangzhiensis* assembly completeness of genome.
**Table S9:** *B. sangzhiensis* genome assembly statistics.
**Table S10:** The statistics on repeat annotation of *B. sangzhiensis* genome.
**Table S11:** The statistics on functional gene annotation of *B. sangzhiensis* genome.
**Table S12:** BUSCO assessment of the Hap1 and Hap2 genome of *B. sangzhiensis*.
**Table S13:** Information of sex‐related SNPs.
**Table S14:** Integrated summary of XY‐like loci, XY‐specific loci, and site‐based differentiated loci in Chr 6 and Chr 2 SLRs in TP and DP population.
**Table S15:** No breakpoint‐like signatures at the Chr 2 SLR boundaries: resequencing CRAM‐based diagnostics (TP population).
**Table S16:** DEGs of gonadal transcriptome data in *B. sangzhiensis*.
**Table S17:** Genes in sex‐linked region on Chr 6 in *B. sangzhiensis*.
**Table S18:** KEGG analysis of genes in sex‐linked region on Chr 6 *B. sangzhiensis*.

## Data Availability

The raw sequence data generated in this study, including whole genome sequencing data, resequencing data and the RNA‐seq data, have been deposited in the NCBI BioProject database under accession number PRJNA1229466 (https://dataview.ncbi.nlm.nih.gov/object/PRJNA1229466). The chromosome‐level genome assembly has been deposited in NCBI under accession number JBWCAK000000000.
