# Supplementary material for: Genome‐Wide Characterization of Sex‐Linked Regions in the Sangzhi Horned Toad (Boulenophrys sangzhiensis) Reveals Complex Sex Determination Mechanisms
Source: Mol Ecol Resour. 2026 Apr 8;26(3):e70136. doi: 10.1111/1755-0998.70136 (PMC13059060; doi:10.1111/1755-0998.70136)
Supplement: Supplementary file 1 — Figure S1: Geographic location of the sampling sites of B. sangzhiensis in the Badagong Mountain National Nature Reserve, Hunan Province, China. The right panel shows two specific collection sites: Tiangping Mountain and Doupeng Mountain within the Sangzhi county These localities represent the distribution range of B. sangzhiensis used for genomic and transcriptomic sampling. Figure S2: Gonadal tissues of sexually mature male (a) and subadult female individuals at different developmental stages (b, c). T indicates the testes, and O indicates the ovaries. All individuals were derived from the resequencing dataset used in this study. Figure S3: The chromosome (a) and whole‐genome (b) Hi‐C interaction maps of B. sangzhiensis at 500 kb resolution. The colour ranging from light to dark indicate increasing contact frequency between genomic loci, with darker shades indicating stronger interactions. Hi‐C contact maps showed 13 strong interaction blocks corresponding to the 13 chromosomes of B. sangzhiensis. Figure S4: Whole‐genome macrosyntenic relationships between Boulenophrys sangzhiensis and its closely related species ( L. tengchongense , L. promustache , L. guangxiense , L. leishanense , L. liui , and L. boringii ). Each line connects orthologous genomic regions between homologous chromosomes. Chr 6 of B. sangzhiensis shows a clear one‐to‐one correspondence with Chr 6 of its relatives, with no evidence of interchromosomal fusion or translocation, supporting the structural integrity of the B. sangzhiensis assembly. Figure S5: Chromosome anchoring and assembly integrity of B. sangzhiensis haplotypes. (a) Hi‐C contact maps of haplotype 1 (Hap1) and haplotype 2 (Hap2) at 1‐Mb resolution. The dense and continuous diagonal signals indicate high assembly continuity and accurate chromosome anchoring. (b) Distribution of contigs across chromosomes for Hap1 and Hap2. Grey bars represent chromosome length, while coloured blocks represent contigs of different size ranges (5 Mb). [file MEN-26-e70136-s002.pdf]

MOLECULAR ECOLOGY RESOURCES

**Supplemental Information for:**

# Genome-wide characterization of sex-linked regions in the Sangzhi Horned Toad (*Boulenophrys sangzhiensis*) reveals complex sex determination mechanisms

Chun H. Huang[1] Si Y. Xie[1] Jun Li[1] Wan Y. Chen[1] Fu Y. Qiu[1] Mian Zhao[1] Wei Liu[2] Chun L. Liao[2] Hua Wu[1*]

[1]Hubei Key Laboratory of Genetic Regulation and Integrative Biology, School of Life Sciences, Central China Normal University, Wuhan, 430079, China
[2]Badagong Mountain National Nature Reserve, Sangzhi, Hunan, China
*Corresponding author: wuhua@ccnu.edu.cn; telephone: 86-027-67867827.

## Table of Contents:


| **SUPPLEMENTARY METHODS:** | |
|---|---|
| **Collection and Identification** | Page 4 |
| **Sequencing, Assembly and Quality Assessment** | Page 4 |
| **Genome Annotation** | Page 6 |
| **Haploid Assembly** | Page 7 |
| **Cytogenetic karyotype analysis of *B. sangzhiensis*** | Page 8 |
| **Genomic Data and Chromosome Synteny Analysis** | Page 8 |
| **Resequencing, Variants Calling and Filtering** | Page 9 |
| **Identify Sex Chromosome and Sex-Linked Region** | Page 10 |
| **No breakpoint-like signatures at the Chr 2 SLR boundaries: resequencing CRAM-based diagnostics** | Page 11 |
| **Transcriptome Sequencing and Analysis** | Page 12 |
| **Comparative and Structural Analysis of the Candidate Gene** | Page 12 |
| **SUPPLEMENTARY FIGURES:** | |
| **Figure S1. Geographic location of the sampling sites of *B. sangzhiensis*** | Page 17 |
| **Figure S2. Gonadal tissues of mature male and subadult female individuals** | Page 18 |
| **Figure S3. Hi-C interaction maps of *B. sangzhiensis* at 500kb resolution.** | Page 19 |
| **Figure S4. Whole-genome macrosyntenic relationships between *B. sangzhiensis* and its closely related species** | Page 20 |
| **Figure S5. Chromosome anchoring and assembly integrity of *B. sangzhiensis* haplotypes** | Page 21 |
| **Figure S6. Validation of the assembly integrity on Chr 6 in *B. sangzhiensis*** | Page 22 |

| | |
|---|---|
| **Figure S7. Chromosomal collinearity and structural variation between the two pseudo-haplotypes** | Page 23 |
| **Figure S8. Change-point analysis of GC content of *B. sangzhiensis*** | Page 24 |
| **Figure S9. $F_{ST}$ values and in InDels density between 20 males and 20 females across all chromosomes** | Page 25 |
| **Figure S10. Heat map of linkage disequilibrium (LD) in sex linked region (SLR) on Chr 2 from 20 males and 20 females** | Page 26 |
| **Figure S11. Integrative Genomics Viewer (IGV) screenshot showing the sex-specific coverage bias in the putative sex-linked region on Chr 6 of *B. sangzhiensis*** | Page 27 |
| **Figure S12. PCA plot for SNPs in SLR on Chr 2 and Chr 6 in different populations** | Page 28 |
| **Figure S13. Mapping of Sanger sequencing PCR product to the genome region** | Page 29 |
| **Figure S14. Visualization of the sex -specific coverage in *B. sangzhiensis* using IGV** | Page 30 |
| **Figure S15. Genomic differentiation between M1 (12 males) and 5 females in *B. sangzhiensis* from the TP population.** | Page 31 |
| **Figure S16. Heatmap and NJ tree based on informative Chr 6 loci in the TP population** | Page 32 |
| **Figure S17. Sex-linked signal detection on Chr 2 and Chr 6 in *B. sangzhiensis* (5 M2 males vs. 5 females) from the TP population (window size = 50 kb)** | Page 33 |
| **Figure S18. Heat map of LD in SLR on Chr 2 and LD decay analysis on Chr 6** | Page 34 |
| **Figure S19. Genotype heatmap of the sex-linked region on Chr 6 in *B. sangzhiensis* (5 M2 males and 5 females)** | Page 35 |
| **Figure S20. Individual-level concordance between sex-linkage signals on Chr 6 and Chr 2 in the TP population** | Page 36 |
| **Figure S21. Genomic differentiation between all males and 5 females (a) and M2 and 5 females (b), M1 and 5 females (c) and M1 and M2 (d) in the DP population.** | Page 37 |
| **Figure S22. Phylogenetic analysis and multiple sequence alignments of *Hsd11b2*** | Page 38 |
| **Figure S23. The expression level of *Hsd11b2* gene on Chr 2 in *B. sangzhiensis*** | Page 39 |
| **Figure S24. Characterize of *Nlrp14* gene in *B. sangzhiensis*** | Page 40 |
| **Figure S25. Characterize of candidate ZNF genes on Chr 6** | Page 41 |
| **Figure S26. Chromosomal structural variants of *B. sangzhiensis*, *X. tropicalis* and *L. leishanense*** | Page 42 |
| **Figure S27. Chromosomal structural variants of *B. sangzhiensis*, *R. temporaria* and *L. leishanense*** | Page 42 |
| **Figure S28. Chromosomal structural variants among *R. temporaria*, *B. sangzhiensis*, and *X. tropicalis*** | Page 43 |
| **Figure S29. The distribution of repetitive sequences and Gene copies rates of *B. sangzhiensis*** | Page 44 |
| **Figure S30. Unmodified original gel image corresponding to Figure 3d in the main text** | Page 45 |
| **SUPPLEMENTARY TABLES (provided in separate Excel file):** | |
| **Table S1. Sample information** | |
| **Table S2. Species identification result of resequencing sample** | |
| **Table S3. The alignment statistics of the resequencing sample sequences against the genome of *B. sangzhiensis*** | |

| | |
|---|---|
| **Table S4. Library data statistics of SMRT sequencing by using the PacBio sequencing platform** | |
| **Table S5. Assembly result of HiFiasm** | |
| **Table S6. Mapping statistics of short-read and PacBio HiFi read alignments to the assembled genome** | |
| **Table S7. Statistics on homozygous rate and heterozygous rate** | |
| **Table S8. *B. sangzhiensis* assembly completeness of genome** | |
| **Table S9. *B. sangzhiensis* genome assembly statistics** | |
| **Table S10. The statistics on repeat annotation of *B. sangzhiensis* genome** | |
| **Table S11. The statistics on functional gene annotation of *B. sangzhiensis* genome** | |
| **Table S12. Hi-C assembly statistics and BUSCO assessment of two pseudo-haplotypes** | |
| **Table S13. Information of sex-related SNPs** | |
| **Table S14. Integrated summary of XY-like loci, XY-specific loci, and site-based differentiated loci in Chr 6 and Chr 2 SLRs in the TP and DP population** | |
| **Table S15. DNA-level mapping of the *B. sangzhiensis* Chr 2 SLR interval to the *R. temporaria* genome** | |
| **Table S16. DEGs of gonadal transcriptome data in *B. sangzhiensis*** | |
| **Table S17. Genes in SLR on Chr 6 in *B. sangzhiensis*** | |
| **Table S18. KEGG analysis of genes in SLR on Chr 6 *B. sangzhiensis*** | |

## SUPPLEMENTAL METHODS

### Sample Collection and Identification

All samples used in this study were collected from Tianping Mountain and Doupeng Mountain, two sites within the Badagong Mountain National Nature Reserve, Sangzhi county, Zhangjiajie, Hunan Province, China (Figure S1). All tissues used for genome and transcriptome sequencing were immediately flash-frozen in liquid nitrogen after dissection.

For whole-genome DNA sequencing, we collected muscle from one adult male. Genomic DNA was extracted using the DNeasy Blood & Tissue Kit (Qiagen, Valencia, CA, USA). For transcriptomic sequencing, seven tissues (brain, liver, heart, kidney, muscle, gonads and spleen) were collected from the same individual. RNA from these tissues was used to quantify gene expression and assist in genome annotation, ensuring comprehensive coverage of expressed genes across multiple tissues while maintaining consistency with the assembled genome. For the Hi-C and HiFi libraries preparation, liver tissue from the same individual was used.

For genome resequencing, 20 adult females and 20 males of *B. sangzhiensis* were captured during breeding season (July to August), anesthetized with 0.2% ethyl-3-aminobenzoate methanesulfonate (MS222), euthanized, and preserved in anhydrous ethanol (Table S1). DNA extraction was carried out using EasyPure® Genomic DNA Kit (TransGen Biotech, China).

Species identity was verified through molecular markers by amplifying the 16S (Sense: 5' CAGAAGCGAGGATATTCCCATAAGACGA3'; Anti-sense: AACAAACGAACCTTTAGTAGCGGTTGCA) and RAG1 (Sense: 5' TTTCCACCAGTACGGACGTTGGGATTAT3'; Anti-sense: TTCTTTCTGAGGTGTTTGTCCAGGGTTA) genes (Table S2). In addition, the resequencing reads were also aligned with the reference genome to assess the accuracy of species identification, based on properly rate (%), which represents the proportion of paired-end reads that are correctly aligned to the reference sequence, with the expected orientation and insert size (Table S3).

Phenotypic sex was determined based on gonadal morphology (Figure S2). In *B. sangzhiensis*, gonadal differentiation becomes apparent at approximately Gosner stage 28. By stage 46, testes and ovaries can be clearly distinguished, with ovaries being particularly easy to identify. Among the 40 individuals examined, 32 were adults and 8 were juveniles (Table S2). These juveniles were post-metamorphosis individuals. Their gonads exhibited well-developed ovaries, allowing reliable identification as females (Figure S2).

### Sequencing, Assembly and Quality Assessment

To obtain a high-quality chromosome-level genome for *B. sangzhiensis*, we employed a combination of Illumina paired-end sequencing (HiSeq), Single Molecule Real-Time sequencing (SMRT, PacBio), and Chromatin conformation capture (Hi-C) technologies.

Initially, paired-end libraries with insert sizes of 300~350 bp were constructed from a male individual and sequenced on the Illumina NovaSeq 6000 system (Illumina, San Diego, CA, USA). After the removal of sequencing adapters, contaminant reads (mitochondrial, bacterial, and viral sequences), and low-quality reads, we obtained 200.6 Gb clean reads. Based on the high-

quality reads, we estimated the genome size of the *B. sangzhiensis* to be 2,549 Mb using a K-mer–based analysis (K=17).

Genomic DNA extracted from the muscle and liver was used to construct sequencing libraries using the PacBio Sequel II Platform. Genomic DNA was sheared to approximately 20 kb fragments using a g-TUBE device (Covaris, Woburn, MA, USA), purified and concentrated with AmpureXP beads (Agencourt, Beverly, MA, USA). The sheared DNA was then used for Single Molecule Real-Time (SMRTbell) library preparation with the SMRTbell Express Template Prep Kit 2.0 (Pacific Biosciences, Menlo Park, CA, USA) following the PacBio 20 kb template preparation protocol (Ardui et al., 2018). The SMRTbell library, after quality control, was sequenced on the PacBio Sequel II platform to generate highly accurate long high-fidelity reads. We recovered 85,393,946,244 bp CCS (HiFi) reads (Table S4), with an average length of 16,890.83 bp. HiFi reads were *de novo* assembled using HiFiasm v0.19.6 (https://github.com/chhylp123/hifiasm) with parameters "--n-hap 2 --primary" to obtain the primary assembly. The preliminary assembly was further processed using Purge_haplotigs v1.0.4 (Roach, Schmidt, and Borneman 2018) to remove redundant heterozygous contigs based on read-depth distribution and sequence similarity, thereby eliminating heterozygous duplications. The initial assembly yielded a genome size of 3,084,590,420 bp, which was reduced to 2,873,347,963 bp after deduplication (Table S5).

To ensure that the final assembly represented the nuclear genome of the target species, the genome assembly was divided into 1,000 bp fragments and aligned to the NCBI nucleotide (NT) database using BLASTN 2.11.0 (parameters -evalue 0.00001 -max_hsps 1) (Altschul et al. 1990). Only the best hit for each fragment was retained. Fragments that matched prokaryotic or organellar (mitochondrial or chloroplast) sequences were identified as potential contaminants. Contigs were removed only when a substantial proportion of their sequence was covered by such contaminant hits, considering both alignment identity and coverage. This conservative filtering strategy avoided erroneous removal of genuine nuclear sequences containing integrated organellar fragments. To evaluate the quality of the assembly, the statistics of the short reads and Hifi reads mapping rate and coverage were summarized with BWA (v0.7.12-r1039) (Li and Durbin 2009) and minimap2 (v2.24), respectively. The mapping rate of long-read data to the assembly was 99.93% (~99.98× coverage), and that of short-read data was 99.29% (~99.74× coverage) (Table S6). The resulting short-read alignments were processed using SAMtools (Li et al. 2009; parameters sort -m 1G) and Picard (http://broadinstitute.github.io/picard/) to sort, mark duplicates, and index the BAM files. Variant calling was performed with GATK4 (https://gatk.broadinstitute.org/) following the best practices workflow. Based on the identified SNPs and InDels, homozygous and heterozygous variant rates were calculated to evaluate genome accuracy and heterozygosity (Table S7). Genome completeness was further assessed using Benchmarking Universal Single-Copy Orthologs (BUSCO) (v5.3.1) (Simão et al. 2015) method, based on the presence of evolutionarily conserved single-copy orthologs. The analysis was performed in genome mode using the metazoa_odb10 dataset, which is suitable for metazoan and amphibian genomes. The parameters used were "-i -o -l -m genome -f -t", following the standard BUSCO pipeline. The *B. sangzhiensis* genome exhibited high completeness, with 96.6% of BUSCO genes identified as complete (93.5% single-copy and 3.1% duplicated), while 1.8% were fragmented and 1.6% were

missing (Table S8). These metrics confirmed the high accuracy and completeness of the assembly.

To improve assembly contiguity and anchor the assemblies into chromosomes, a Hi-C library with insert sizes of 300~350 bp was constructed from the same male individual, and sequenced on the BGISEQ platform using MGI DNBSEQ T7 sequencing (BGI Tech, Shenzhen, China), yielding 1,637,568,172 raw reads (245.6 Gb). After quality filtering and adapter removal using Fastp (v0.23.2) and quality assessment with FastQC (v0.11.3), 245.4 Gb clean reads were retained for downstream analysis. The Hi-C reads were processed using HiCUP (v0.7.2) (Wingett et al. 2015), which performs adapter truncation, mapping to the reference genome, removal of invalid ligation products, and deduplication to generate high-quality valid pairs. Then, the contigs were anchored into chromosomes by Hi-C sequencing reads through the Juicer v1.6 (Durand et al. 2016) and 3D-DNA v180922 (Dudchenko et al. 2017; parameters -r 0). After correction of misoriented or misplaced contigs using Juicebox (v1.11.08) (Durand et al. 2016), the initial 477 contigs were re-oriented and joined according to Hi-C contact frequencies (reflecting the principle that spatially adjacent genomic regions show stronger interaction signals), resulting in 13 pseudo-chromosomes with a total assembly size of 2.8 Gb. The final assembly exhibited a contig N50 of 29.96 Mb, a scaffold N50 of 335.29 Mb, and a chromosome anchoring rate of 99.32% (Table S9). Hi-C contact maps were constructed and displayed clear diagonal interaction patterns across each chromosome, confirming high contiguity and accurate scaffolding (Figure S3).

**Genome Annotation**

Repetitive element annotation. Repetitive sequences were annotated using a combination of homology-based and de novo methods (Table S10). For homology-based identification, RepeatMasker (http://www.repeatmasker.org) and RepeatProteinMask (Tarailo-Gravoac and Chen 2009) were used to detect transposable elements (TEs), based on the RepBase library (http://www.girinst.org/repbase) (Jurka et al. 2005). For *de novo* prediction, RepeatModeler (Price et al. 2005), TRF v4.09 (Benson 1999; parameters 2 7 7 80 10 50 2000 -d -h) and LTR-FINDER (Xu and Wang 2007) were used to build a *de novo* repeat library based on our genome sequences. The library yielded consensus sequences and classification information for each repeat family. The RepeatMasker v4.0.9 (parameters -nolow -no_is -norna) was then applied to annotate these genome sequences. All repetitive elements, including interspersed repeats (transposable elements, DNA transposons, retrotransposons) and tandem repeats, were masked prior to gene prediction to reduce false annotations.

Gene annotation. Protein-coding genes were annotated by integrating *de novo*, homology-based, and transcriptome-based methods in a repeat-masked genome (Table S11). Consensus gene structures were generated by integrating the homolog protein prediction and de novo prediction. For *de novo* prediction, AUGUSTUS v3.4.0 (Stanke et al. 2006) and Genscan (http://genes.mit.edu/GENSCAN.html) were used to predict protein-coding genes. For homology-based prediction, closely related amphibians (*Nanorana parkeri*, *Xenopus tropicalis*, *Leptobrachium leishanense*, and *Quasipaa spinosa*) were primarily used to guide gene structure prediction and assess annotation consistency. Protein sequences from more distantly related vertebrates (*Mus musculus* and *Homo sapiens*) were included to provide further validation of conserved genes and functional annotation. All reference protein sequences were aligned to

repeat-masked genome using Miniprot v0.11-r234 (parameters --gff-only -O 11 -E 1 -F 23 -C 1 -B 5 -G 200000 -j 1) to identify homologous protein-coding regions and generate preliminary gene models. For transcriptome-based prediction, short-read RNA-Seq data from seven tissues (brain, liver, heart, kidney, muscle, gonads, and spleen) of the same individual were used to assist genome annotation. 61,701,874 reads were mapped to the genome using TopHat, and transcript assemblies were constructed with Cufflinks (Trapnell et al. 2010). The assembled transcripts were incorporated as transcriptomic evidence in the final gene annotation. All genes predicted from above three approaches were integrated into a non-redundant, high-confidence gene set using MAKER2 v2.31.10 (Carson and Mark 2011) with the parameters: max_dna_len = 3000000, min_contig = 10000, pred_flank = 500, and min_protein = 30. The completeness of the predicted gene models was evaluated using CEGMA (Parra, Bradnam, and Korf, 2007). The results from CEGMA were incorporated into the HiCESAP pipeline (Gooalgene Co., Ltd., Wuhan, China; https://www.gooalgene.com/) to refine and integrate gene models, resulting in a final high-confidence gene set containing 21,775 protein-coding genes for *B. sangzhiensis*. To assign gene functions, the predicted gene sequences were searched against the multiple public databases, including SwissProt, TrEMBL (Bairoch and Apweiler 2000), KEGG (Kanehisa and Goto 2000), InterPro (Zdobnov and Apweiler 2001), and Gene Ontology (GO) (Ashburner et al. 2000). Annotation completeness was assessed using BUSCO v5.3.1 (Simão et al. 2015) with the metazoa_odb10 database, yielding a completeness score of 95.8%, indicating a high-quality annotation.

Non-coding RNA annotation. tRNA identified using tRNAscan-SE software (Lowe and Eddy 1997). Ribosomal RNA (rRNA) was annotated by BLASTN searches using homologous rRNA sequences from related species. MicroRNAs (miRNA) and small nuclear RNAs (snRNA) were predicted using INFERNAL with the Rfam database (Griffiths-Jones et al. 2005).

**Haploid Assembly**

The high-quality HiFi data obtained above were subsequently assembled into two pseudo-haplotypes (Hap1 and Hap2) using Hifiasm (v0.19.6) (https://github.com/chhylp123/hifiasm) in combination with Hi-C data to improve contig phasing. Because parental genome information was unavailable, the resulting Hap1 and Hap2 assemblies represent partially phased pseudo-haplotypes rather than true parental haplotypes. Contigs may be misassigned between the two assemblies. To mitigate potential misassignments, contigs from Hap1 and Hap2 were merged and re-clustered using HapHiC, which leverages Hi-C interaction signals to reassign contigs and improve pseudo-haplotype consistency. This procedure maximizes local phasing accuracy, although long-range phasing across entire chromosomes could not be fully resolved.

We generated two high-quality haploid genomes of a male *B. sangzhiensis*. The Hap1 assembly totaled 2.71 Gb and was scaffolded to 2.68 Gb at the chromosome level, forming 13 pseudo-chromosomes with a 98.88% anchoring rate. The Hap2 assembly spanned 2.78 Gb, scaffolded to 2.74 Gb with a 99.28% anchoring rate. Completeness was assessed by BUSCO V4.0.1 (Simão et al. 2015) with the tetrapoda_odb10 database (Table S12). Hi-C contact maps were constructed and displayed clear diagonal interaction patterns across each chromosome, confirming high contiguity and accurate scaffolding (Figure S4). To further verify the structural integrity, PacBio HiFi reads were remapped to the final assembly using Minimap2 v2.24 (Li 2018), which showed continuous coverage across the contact breakpoint regions, indicating the

absence of assembly gaps or misjoins (Figure S5). Two pseudo-haplotypes were used for structural rearrangement investigations in *B. sangzhiensis*.

**Cytogenetic karyotype analysis of *B. sangzhiensis***

Three Adult female *B. sangzhiensis* individuals were collected from Doupeng Mountain. Metaphase chromosomes were prepared from the bones following the method described previously (Xie et al. 2025), with slight adaptations as follows. Colchicine was intraperitoneally injected into individuals' abdominal cavity at a dosage of 1–5 μg/g body weight, and individuals were sacrificed 3 hours after injection. The femur and tibia were firstly rinsed with Phosphate-buffer saline (PBS), and flushed with 0.34% KCl hypotonic solution to extract bone marrow cells. The cell suspension was filtered and centrifuged to collect the bone marrow cells, which were then resuspended in 0.34% KCl and dropped onto glass slides for hypotonic treatment for 30 min. The slides were subsequently fixed with a mixed fixative solution (ethanol:acetic acid:water = 1:2:3) for 2 h, followed by dehydration with absolute ethanol for 30 min. After washing the slides 3–4 times with a fixative solution (ethanol:acetic acid = 1:2) and air-drying, the preparations were stained with 10% Giemsa solution for 30 min.

**Genomic Data and Chromosome Synteny Analysis**

Genomic data for *B. sangzhiensis* (male) were generated from our own sequencing library. The genome assemblies of *Leptobrachium leishanensis* (male), *Bufo gargarizans* (male), and *L. boringii* (male) were obtained from (Li et al. 2019), (Lu et al. 2020), and (Xie et al. 2025), respectively. Genome data for *Rana temporaria* (female, GCF_905171775.1), *Xenopus tropicalis* (female, UCB_Xtro_10.0.108), and *Bombina bombina* (male, GCF_027579735.1) were retrieved from the NCBI database. Additionally, genome data of *L. liui* (male), *L. guangxiense* (male), *L. tengchongense* (male), and *L. promustache* (male) were obtained from our laboratory's unpublished genome datasets.

To examine the homology and evolutionary rearrangements of sex chromosomes, unphased genome assemblies of *B. sangzhiensis*, *R. temporaria*, *B. gargarizans*, *X. tropicalis*, and *B. bombina* were compared. Intergenomic collinearity was analyzed in *B. sangzhiensis*. Inter-species structural rearrangements (e.g., inversions, translocations, and duplications) were investigated among *B. sangzhiensis*, *R. temporaria*, *X. tropicalis*, and *L. leishanensis*. Intra-species chromosomal rearrangements were investigated between the phased haplotypes of *B. sangzhiensis*, pseudo-haplotypes one (Hap1) and two (Hap2).

To assess potential chromosomal fusion or fission events, synteny relationships among *B. sangzhiensis* and its close relatives (*L. leishanensis*, *L. boringii*, *L. liui*, *L. guangxiense*, *L. tengchongense*, and *L. promustache*) were analyzed using unphased male genome assemblies.

Synteny blocks among species were identified using the JCVI toolkit (Tang et al., 2008) to examine sex chromosome homology and to detect potential chromosomal fusion or fission events among related taxa. Orthologous gene pairs were detected with the ortholog module of jcvi.compara.catalog using default settings with the --no_strip_names flag retained. Syntenic blocks were filtered using jcvi.compara.synteny screen with a minimum span threshold of 30 genes. Resulting anchors were visualized using jcvi.graphics.karyotype for chromosomal-scale karyotype plots, guided by custom layout files.

Structural rearrangements, including inversions, translocations, and duplications, were detected using SyRI (Goel et al., 2019) in both inter-species and intra-species comparisons. The workflow was as follows: Pairwise genome alignment was performed using minimap2 (minimap2 -ax asm5 --eqx Bsan.chr.fa Xtrop.chr.fa > Bsan.Xtro.sam); SYRI then was run to detect structural rearrangements (syri -c Bsan.Xtro.sam -r Bsan.chr.fa -q Xtrop.chr.fa -k -F S --prefix Bsan.Xtro); Regions labeled SYN were interpreted as collinear blocks; INV, TRANS, DUP and DEL were cataloged as structural variants. The results were finally visualized using plotsr.

Furthermore, to identify and visualize genomic collinearity within the *B. sangzhiensis* genome, we first conducted an all-vs-all BLASTP alignment of protein-coding genes (E-value ≤ 1e-5) and detected syntenic blocks using MCScanX with parameters: at least 10 genes per block (–s 10) and a maximum of 25 intervening genes (–m 25). Chromosome identifiers were ordered by descending chromosome length. Circos plots were generated in TBtools (Chen et al., 2018a) to visualize intra- and inter-chromosomal collinearity.

**Resequencing, Variants Calling and Filtering**

Paired-end libraries with insert sizes of 300~350 bp was constructed from 20 females and 20 males, and sequenced on the BGISEQ platform using MGI DNBSEQ T7 sequencing (BGI Tech, Shenzhen, China), Sequencing was designed to achieve approximately 15~20× coverage per individual based on the estimated genome size. The actual average sequencing depth (MeanDepth) across all samples was 16.1× (Table S3).

To ensure high-quality data for analysis, all raw data were quality-checked, demultiplexed, and filtered by FastQC 0.11.3 (https://www.bioinformatics.babraham.ac.uk/projects/fastqc). Sequencing adapters and low-quality reads were removed from the raw data using the fastp with default parameters (Chen et al. 2018b). The quality-controlled sequencing data were then aligned to the reference genome using BWA (v0.7.12-r1039) (Li and Durbin 2009) with the parameters: mem -M -t 16. The resulting sam files were converted to bam files and sorted with SAMtools v1.9 (Li et al. 2009), followed by the removal of duplicate reads using Picard 1.124 (http://broadinstitute.github.io/picard/). Single nucleotide polymorphisms (SNPs) and insertion–deletion polymorphisms (InDels) were identified using GATK4 (https://gatk.broadinstitute.org). To improve the accuracy of variants calling, we further filtered the merged SNP results using GATK4 filters (QD < 2.0 || FS > 60.0 || MQ < 40.0 || MQRankSum < -12.5 || ReadPosRankSum < -8.0). InDels were applied indel-specific hard filters with GATK4 VariantFiltration (QD < 2.0, FS > 200.0, ReadPosRankSum < −20.0, SOR > 10.0), and only sites labeled as PASS were retained. The resulting SNPs and InDels were further filtered using vcftools (Danecek et al. 2011) to enforce biallelic status and population-level thresholds (--min-alleles 2 --max-alleles 2 --maf 0.05 --min-meanDP 5 --max-missing 0.75). After quality control, a total of 36,260,227 SNPs and 6,547,198 InDels were retained for subsequent analyses. Within SNPs, linked loci (r2 > 0.2) were further removed by considering a window of 50 SNPs and a step-size of 10 SNPs as conducted in Plink v1.90 (Purcell et al. 2007), which finally generated a LD-pruned SNP dataset (Purcell et al., 2007).

**Identify Sex Chromosome and Sex-Linked Region**

Candidate sex-linked regions were identified by integrating three independent genomic signals: (1) differences in mapped read coverage between sexes, (2) variation in SNP density or the presence of sex-linked SNPs, and (3) elevated genetic differentiation ($F_{ST}$) between males and females based on both SNP and InDel. Integrating multiple complementary signals helps minimize false positives caused by local copy-number variation or assembly artifacts.

Genomic coverage analysis: In a highly degenerated non-recombining region of a Y chromosome, males possess only one copy of the X chromosome. Therefore, the sequencing read coverage in males is expected to be approximately half that of females on the corresponding X chromosome or autosomes (Palmer et al. 2019). Reads from each sample were aligned to the reference genome, and only uniquely mapped reads with mapping quality ≥15 were retained. Coverage of each individual was calculated using PanDepth v2.19 (Yu et al. 2024) with a window size of 50 and 1kb. The 50 kb window was primarily used for log2(M:F) coverage ratios across the genome, while the 1 kb window was applied to Chr 2 to capture finer-scale structural variations and more detailed genomic differentiation in sex-linked regions. The command used was: pandepth -f Bsan.genome.fa -i input.sample.recaled.cram -o output.sample -q 15. The samples were divided into two groups: male and female. The average coverage for male and female groups was calculated, and the difference was computed using the formula: $\log_2$ (average male coverage + 0.01) - $\log_2$ (average female coverage + 0.01).

SNP density analysis: In young or recently differentiated sex chromosomes, the X and Y regions may still retain substantial sequence homology. As a result, Y-specific reads can map to the homologous X region, leading to an increased SNP density in males relative to females. In contrast, highly diverged X-linked regions (where the Y homolog is degenerated) are expected to show little or no SNP signal in males (Palmer et al. 2019). SNPs were filtered using PLINK v.1.90 (Purcell et al. 2007; parameters --maf 0.05, --geno 0.1) and were then used to calculate SNP density across the genome with VCFtools (Danecek et al. 2011) in 50 kb windows. Differences were expressed as $\log_2$ (average male SNP density + 0.01) - $\log_2$ (average female SNP density + 0.01).

Identification of sex-linked SNPs: Sex-linked SNPs were identified based on allele frequency differences between males and females. In an XY system, loci that are heterozygous in males but homozygous in females are expected to show male allele frequencies near 0.5 and female allele frequencies near 0 (Jeffries et al. 2018). SNPs were filtered (--maf 0.05, --geno 0.25), and allele frequencies were estimated for both sexes. SNPs were defined as XY-linked if the X allele frequency was ≥0.95 in females and between 0.4–0.6 in males, or as ZW-linked if the Z allele frequency was ≥0.95 in males and between 0.4–0.6 in females. Both XY-linked and ZW-linked sets were retained as candidate sex-linked SNPs (Table S13).

$F_{ST}$ analysis: To further identify potential sex-linked regions, genetic differentiation between male and female populations was quantified (Gammerdinger, Toups, and Vicoso 2020; Toups et al. 2018; Rodrigues et al. 2017; Liu et al. 2024; Kina et al. 2025). This approach is especially useful in species with homomorphic sex chromosomes, where sex-determining loci may still show limited differentiation. SNPs and InDels are recognised as characteristic features of sex-determining and sex-linked regions and were analyzed to capture structural differences between the sexes (Liu et al. 2024; Kina et al. 2025). Mean $F_{ST}$ values for both SNPs and InDels were calculated using VCFtools (Danecek et al. 2011) in 50 and/or 1 kb windows. Genomic

windows in the top 1% of mean $F_{ST}$ values were considered candidate sex-linked regions. Visualization was performed using the CMplot package in R.

**XY-like and XY-specific loci–based individual-level concordance test**

Data and regions. We analyzed resequencing SNP genotypes from TP (n = 22; 5F/12M1/5M2) and DP (n = 18; 15F/2M1/1M2). Two candidate SLRs were examined: Chr 2 (372.8–375.6 Mb) and Chr 6 (105.9–147.5 Mb). Genotypes were exported from filtered biallelic SNP VCFs using VCFtools v0.1.16 (--012) to generate .012, .012.indv, and .012.pos files, where 0/1/2/−1 denote homozygous reference, heterozygous, homozygous alternative, and missing genotypes, respectively.

**(1) Enrichment-based XY-like loci and reciprocal ZW-like loci**

Females were defined as fixed homozygous for the same allele only if all called female genotypes were homozygous and identical (all 0/0 or all 1/1; no female heterozygotes and no mixture of the two homozygous states). Male heterozygosity rate was calculated as the proportion of called male genotypes that were heterozygous (0/1). XY-like loci were defined as female fixed-homozygosity together with enrichment of male heterozygosity, evaluated under three contrasts in TP (F vs AllM, F vs M1, F vs M2). The reciprocal ZW-like screen required males to be fixed homozygous for the same allele together with enrichment of female heterozygosity; we report a relaxed criterion (female_het_rate ⩾ 0.30) and a strict criterion matching the ZW expectation (female_het_rate = 1.0).

Threshold strategy. In TP, all females were required to be called (nF_called = 5) and fixed-homozygous. Because pooled males (AllM) include two genotypic classes (M1 and M2) and heterozygosity enrichment can be diluted by mixture, we used a relaxed threshold for AllM (male_het_rate ⩾ 0.30; nM_called ⩾ 15) and a stricter threshold for M2 (M2_het_rate ⩾ 0.60; nM2_called ⩾ 4), with analogous criteria for M1 (M1_het_rate ⩾ 0.30; nM1_called ⩾ 10). In DP, male sample size was limited (3 males; M2 n = 1), so DP analyses were treated as supportive; thresholds were chosen to reflect the discrete heterozygosity proportions possible with small n (e.g., AllM_lo: male_het ⩾ 1/3; AllM_hi: male_het ⩾ 2/3), and strict M1 criteria required both M1 males to be heterozygous to minimize 1-of-2 noise.

Overlap, individual scores, and statistics. Overlap among XY-like locus sets was quantified using Jaccard similarity (intersection/union). For individual-level sex-linkage scores, we used the XY-like loci identified from the F vs M2 contrast as a fixed high-confidence marker panel and calculated, for each individual, the proportion of heterozygous genotypes across these loci separately for Chr 6 and Chr 2. Associations between Chr 6 and Chr 2 scores were tested using Spearman correlation. Scores were also dichotomized using predefined thresholds (Chr 6 ⩾ 0.6; Chr 2 ⩾ 0.3) and evaluated using Fisher's exact test.

**(2) Strict male-specific SNP screen and strict reciprocal female-specific SNP screen**

XY-specific (strict) loci required females fixed homozygous for the same allele (all 0/0 or all 1/1; all females called) and all males heterozygous (0/1; all males called), evaluated separately for F vs AllM, F vs M1, and F vs M2. The reciprocal ZW-specific (strict) loci required males fixed homozygous for the same allele (all 0/0 or all 1/1; all males called) and all females heterozygous (0/1; all females called). DP strict results are considered supportive only due to M2 n = 1.

**Transcriptome Sequencing and Analysis**

To investigate biased gene expression, we collected gonad tissues from 6 adult females and 6 adult males. Total RNA was isolated using the TRIzol reagent (Invitrogen, Carlsbad, CA, USA) followed by treatment with RNase-free DNase I (Promega, Madison, WI, USA) according to the manufacturers' protocols. RNA quality was checked using an Agilent 2100 Bioanalyzer. Illumina RNA-seq libraries were prepared for 12 samples and sequenced on a HiSeq 2500 system with a PE150 strategy following the manufacturer's instructions. RNA-seq reads were mapped to the reference genome using HISAT2 (Kim et al. 2015), and the reads mapped to each gene were counted using featureCounts v1.6.2 (Liao et al. 2014).

The expression level of predicted transcripts in each RNA-seq library was calculated as fragments per kilobase of transcript per million mapped reads (FPKM) and differential expression genes (DEGs) were identified using the DESeq2 R package (Love et al. 2014) with false discovery rate (FDR) corrected *p* value (*q*-value) <0.05 and $|\log_2$ (fold-change)$| > 1$.

Sex-biased genes (Table S16) were integrated with genomic differentiation metrics, including SNP density, $F_{ST}$, and coverage, to identify candidate genes involved in sex determination and differentiation. We examined the genome-wide distribution of sex-biased genes. Expression patterns of multicopy candidate genes located in regions with high $F_{ST}$ differentiation and elevated $\log_2$(M:F) coverage ratio were also analyzed. Notably, these analyses were based on adult gonadal RNA-seq data; therefore, the observed sex-biased expression likely reflects downstream gonadal physiology rather than the primary embryonic master sex-determining signal. Accordingly, we interpret the differential expression results as supportive, but not definitive, evidence for candidate genes within the SLRs.

**Comparative and Structural Analysis of the Candidate Gene**

**(1) Copy Number and Chromosomal Location Analysis**

To determine the copy number and chromosomal positions of *Hsd11b2* in *B. sangzhiensis* and its close relatives (*L. boringii* and *L. leishanensis*), gene annotation files were extracted to identify all *Hsd11b2* gene models. To verify sequence completeness and assess divergence, protein sequences of HSD11B2 from *B. sangzhiensis*, *L. boringii*, *L. leishanensis*, *Xenopus laevis* (NP_001086062.1), *Oreochromis niloticus* (Nile tilapia; NP_001266686.1), *Danio rerio* (NP_997885.2), and *Mus musculus* (NP_032315.2) were aligned using MUSCLE (v3.8.1551) and visualized in Geneious (v2023.1).

Orthology inference was performed using OrthoFinder (v2.5.4) to identify single-copy and multi-copy gene families. Orthogroups among *B. sangzhiensis*, *L. boringii*, and L. leishanensis were identified using the following parameters: orthofinder -S diamond -M msa -T fasttree -t 20. In *L. boringii* and *L. leishanensis*, only a single *Hsd11b2* ortholog was detected and located on chromosome 10. Whereas *B. sangzhiensis* contained four *Hsd11b2* copies: two annotated on Chr 2, one on Chr 10, and one unannotated on Chr 4. The one unannotated on Chr 4 was excluded from further analyses.

To validate the chromosomal positions, protein-to-genome mapping was conducted using Minimap2 (v2.20) with the parameter -ax asm5 –eqx. Protein sequences from *L. boringii*, *L. leishanensis*, *X._laevis*, *N._tilapia*, and *D. rerio* were aligned to the *B. sangzhiensis* genome. The mapping results confirmed the copy number and chromosomal locations consistent with the OrthoFinder analysis.

**(2) Multiple Sequence Alignment and Phylogenetic Analysis**

Protein sequences of *Hsd11b2* from *D. rerio*, *O. niloticus* and *X. laevis* were retrieved from NCBI. For *B. sangzhiensis*, *L. leishanensis*, and *L. boringii*, *Hsd11b2* gene IDs were obtained from the genome annotation files and the corresponding protein sequences were extracted from the genome.pep. All sequences were aligned with MUSCLE (v3.8.1551) (Edgar 2004). The alignments were visualized in Geneious (v2023.1). Maximum-likelihood phylogeny was inferred with IQ-TREE (v2.0.3) (Minh et al. 2020) using the ModelFinder-selected model (-m MFP) and 1,000 ultrafast bootstrap replicates (-bb 1000). The resulting tree was visualized and annotated in iTOL(https://itol.embl.de).

**(3) Protein Structure Prediction and Visualization**

Protein structure prediction was conducted using AlphaFold3 (Abramson et al., 2024) to explore potential structural variation in *Hsd11b2*. The full-length amino-acid sequence of *Hsd11b2* was submitted to the AlphaFold3 web server (https://alphafoldserver.com/) with default parameters. Among the generated models, the top-ranked model_0.cif (highest pLDDT confidence score) was downloaded. Secondly, the UniProt database (https://www.uniprot.org/) was used to gather detailed information on the gene's function, family, conserved domains, and known binding sites. Finally, protein structure was visualized in PyMOL (v3.1.3) (DeLano 2002) for structural inspection and figure preparation.

## REFERENCES


Abramson, J., J. Adler, J. Dunger, et al. 2024. "Accurate Structure Prediction of Biomolecular Interactions With Alphafold 3." *Nature* **630**: 493-500.

Altschul, S. F., W. Gish, W. Miller, E. W. Myers, and D. J. Lipman. 1990. "Basic Local Alignment Search Tool." *Journal of Molecular Biology* **215**, no. 3: 403–410.

Ardui, S., A. Ameur, J. R. Vermeesch, and M. S. Hestand. 2018. "Single Molecule Real-Time (SMRT) Sequencing Comes of Age: Applications and Utilities for Medical Diagnostics." *Nucleic Acids Research* **46**: 2159-2168.

Ashburner, M., C. A. Ball, J. A. Blake, D. Botstein, H. Butler, J. M. Cherry, et al. 2000. "Gene Ontology: Tool for the Unification of Biology." *Nature Genetics* **25**, no. 1: 25–29.

Bairoch, A., and R. Apweiler. 2000. "The SWISS-PROT Protein Sequence Database and Its Supplement TrEMBL in 2000." *Nucleic Acids Research* **28**, no. 1: 45–48.

Benson, G. 1999. Tandem Repeats Finder: a Program to Analyze DNA Sequences. *Nucleic Acids Research* **27**, no. 2: 573-580.

Carson, H., and Y. Mark. 2011. "MAKER2: an Annotation Pipeline and Genome-Database Management Tool for Second-Generation Genome Projects." *BMC Bioinformatics* **12**, 491.

Chen, C., H. Chen, Y. He, and R. Xia. 2018a. "TBtools, a Toolkit for biologists Integrating Various Biological Data Handling Tools With a user-Friendly Interface." *BioRxiv* **289660**, no. 10.1101: 289660.

Danecek, P., A. Auton, G. Abecasis, et al. 2011. "The Variant Call Format and VCFtools." *Bioinformatics* **27**, no. 15: 2156-2158.

DeLano, W. L. 2002. "Pymol: An Open-Source Molecular Graphics Tool". *CCP4 Newsletter on Protein Crystallography* **40**, no. 1: 82-92.

Dudchenko, O., S. S. Batra, A. D. Omer, S. K. Nyquist, M. Hoeger, N. C. Durand, M. S. Shamim, I. Machol, E. S. Lander, A. P. Aiden, et al. 2017. “De Novo Assembly of the Aedes aegypti Genome Using Hi-C Yields Chromosome-Length Scaffolds.” *Science* **356**, no. 6333: 92–95.

Durand, N. C., M. S. Shamim, I. Machol, S. S. P. Rao, M. H. Huntley, E. S. Lander, and E. L. Aiden. 2016. “Juicer Provides a One-Click System for Analyzing Loop-Resolution Hi-C Experiments.” *Cell Systems* **3**, no. 1: 95–98.

Edgar, R. C. 2004. “MUSCLE: Multiple Sequence Alignment with High Accuracy and High Throughput”. *Nucleic Acids Research* **32**, no. 5: 1792-1797.

Gammerdinger, W. J., M. A. Toups, and B. Vicoso. 2020. “Disagreement in $F_{ST}$ Estimators: A Case Study from Sex Chromosomes.” *Molecular Ecology Resources* **20**, no. 6: 1517–1525.

Goel, M., H. Sun, W. B. Jiao, and K. Schneeberger. 2019. “SyRI: Finding Genomic Rearrangements and Local Sequence Differences from Whole-Genome Assemblies.” *Genome Biology* **20**: 1-13.

Griffiths-Jones, S., S. Moxon, M. Marshall, A. Khanna, S. R. Eddy, and A. Bateman. 2005. “Rfam: Annotating Non-Coding RNAs in Complete Genomes.” *Nucleic Acids Research* **33**, no. suppl_1: D121-D124.

Jeffries, D. L., G. Lavanchy, R. Sermier, et al. 2018. “A Rapid Rate of Sex-Chromosome Turnover and Non-Random Transitions in True Frogs.” *Nature Communications* **9**, no. 1: 4088.

Jurka, J., V. V. Kapitonov, A. Pavlicek, P. Klonowski, O. Kohany, and J. Walichiewicz. 2005. “Repbase Update, a Database of Eukaryotic Repetitive Elements.” *Cytogenetic and Genome Research* **110**, no. 1–4: 462–467.

Kanehisa, M., and S. Goto. 2000. “KEGG: Kyoto Encyclopedia of Genes and Genomes.” *Nucleic Acids Research* **28**, no. 1: 27–30.

Killick, R., and I. A. Eckley. 2014. “Changepoint: An R Package for Changepoint Analysis.” *Journal of Statistical Software* **58**: 1-19.

Kim, D., B. Langmead, and S. L. Salzberg. 2015. “HISAT: A Fast Spliced Aligner with Low Memory Requirements.” *Nature Methods* 12: 357–360.

Kina, T., M. Hara, S. Hirase, and K. Kikuchi. 2025. “Identification of the Sex Determination Region and the Development of a Marker to Distinguish Males and Females in Megai Abalone (*Haliotis gigantea*).” *Aquaculture Reports* **40**: 102592.

Li, H. 2018. “Minimap2: Pairwise Alignment for Nucleotide Sequences.” *Bioinformatics* **34**, no. 18: 3094–3100.

Li, H., and R. Durbin. 2009. “Fast and Accurate Short Read Alignment with Burrows-Wheeler Transform.” *Bioinformatics* **25**, no. 14: 1754-1760.

Li, H., B. Handsaker, A. Wysoker, T. Fennell, J. Ruan, N. Homer, G. Marth, G. Abecasis, R. Durbin, and 1000 Genome Project Data Processing Subgroup. 2009. “The Sequence Alignment/Map Format and SAMtools.” *Bioinformatics* **25**, no. 16: 2078–2079.

Li, J., H. Yu, W. Wang, C. Fu, W. Zhang, F. Han, and H. Wu. 2019. “Genomic and Transcriptomic Insights into Molecular Basis of Sexually Dimorphic Nuptial Spines in *Leptobrachium leishanense*.” *Nature Communications* **10**, no. 1: 5551.

Liao, Y., G. K. Smyth, and W. Shi. 2014. “FeatureCounts: An Efficient General-Purpose Program for Assigning Sequence Reads to Genomic Features.” *Bioinformatics* **30**: 923–930.

Liu, H., J. Zhang, T. Cui, W. Xia, Q. Luo, S. Fei, and M. Ou. 2024. “Genome-Wide Association Studies (GWAS) and Transcriptome Analysis Reveal Male Heterogametic Sex-Determining

Regions and Candidate Genes in Northern Snakeheads (*Channa argus*).” *International Journal of Molecular Sciences* **25**, no. 20: 10889.

Liu, H., J. Zhang, T. Cui, W. Xia, Q. Luo, S. Fei, et al. 2024. “Genome-Wide Association Studies (GWAS) and Transcriptome Analysis Reveal Male Heterogametic Sex-Determining Regions and Candidate Genes in Northern Snakeheads (*Channa argus*).” *International Journal of Molecular Sciences* **25**, no. 20: 10889.

Love, M. I., W. Huber, and S. Anders. 2014. “Moderated Estimation of Fold Change and Dispersion for RNA-Seq Data with DESeq2.” *Genome Biology* 15: 1–21.

Lowe, T. M., and S. R. Eddy. 1997. “tRNAscan-SE: A Program for Improved Detection of Transfer RNA Genes in Genomic Sequence.” *Nucleic Acids Research* **25**, no. 5: 955-964.

Lu, B., J. Jiang, H. Wu, X. Chen, X. Song, W. Liao, and J. Fu. 2021. “A Large Genome With Chromosome-Scale Assembly Sheds Light on the Evolutionary Success of a True Toad (*Bufo gargarizans*).” *Molecular Ecology Resources* **21**, no. 4: 1256-1273.

Majoros, W. H., M. Pertea, and S. L. Salzberg. 2004. “TigrScan and GlimmerHMM: Two Open Source Ab Initio Eukaryotic Gene-Finders.” *Bioinformatics* **20**, no. 16: 2878–2879.

Minh, B. Q., H. A., Schmidt, O., Chernomor, D., Schrempf, M. D., Woodhams, A., Von Haeseler, and R. Lanfear. 2020. “IQ-TREE 2: New Models and Efficient Methods for Phylogenetic Inference in the Genomic era”. *Molecular Biology and Evolution* **37**, no.5: 1530-1534.

Palmer, D. H., T. F. Rogers, R. Dean, and A. E. Wright. 2019. “How to Identify Sex Chromosomes and Their Turnover.” *Molecular Ecology* **28**, no. 21: 4709-4724.

Parra, G., K. Bradnam, and I. Korf. 2007. “CEGMA: A Pipeline to Accurately Annotate Core Genes in Eukaryotic Genomes.” *Bioinformatics* **23**, no. 9: 1061-1067.

Price, A. L., N. C. Jones, and P. A. Pevzner. 2005. “De Novo Identification of Repeat Families in Large Genomes.” *Bioinformatics* **21**, no. Suppl 1: i351–i358.

Roach, M. J., S. A. Schmidt, and A. R. Borneman. 2018. “Purge Haplotigs: Allelic Contig Reassignment for Third-Gen Diploid Genome Assemblies.” *BMC Bioinformatics* **19**: 1-10.

Rodrigues, N., T. Studer, C. Dufresnes, W. J. Ma, P. Veltsos, and N. Perrin. 2017. “Dmrt1 Polymorphism and Sex-Chromosome Differentiation in Rana temporaria.” *Molecular Ecology* **26**, no. 19: 4897-4905.

Simão, F. A., R. M. Waterhouse, P. Ioannidis, E. V. Kriventseva, and E. M. Zdobnov. 2015. “BUSCO: Assessing Genome Assembly and Annotation Completeness With Single-Copy Orthologs.” *Bioinformatics* **31**, no. 19: 3210-3212.

Stanke, M., O. Keller, I. Gunduz, A. Hayes, S. Waack, and B. Morgenstern. 2006. “AUGUSTUS: Ab Initio Prediction of Alternative Transcripts.” *Nucleic Acids Research* **34**, no. Suppl 2: W435–W439.

Tang, H., J. E. Bowers, X. Wang, R. Ming, M. Alam, and A. H. Paterson. 2008. “Synteny and Collinearity in Plant Genomes.” *Science* **320**, no. 5875: 486-488.

Toups, M. A., N. Rodrigues, N. Perrin, and M. Kirkpatrick. 2018. “A reciprocal translocation radically reshapes sex-linked inheritance in the common frog”. *Molecular Ecology* **28**, no. 8: 1877-1889

Trapnell, C., B. A. Williams, G. Pertea, A. Mortazavi, G. Kwan, M. J. van Baren, S. L. Salzberg, B. J. Wold, and L. Pachter. 2010. “Transcript Assembly and Quantification by RNA-Seq Reveals Unannotated Transcripts and Isoform Switching during Cell Differentiation.” *Nature Biotechnology* **28**, no. 5: 511-515.

Wingett, S., P. Ewels, M. Furlan-Magaril, T. Nagano, S. Schoenfelder, P. Fraser, and S. Andrews. 2015. “HiCUP: Pipeline for Mapping and Processing Hi-C Data.” *F1000Research* **4**: 1310.

Xie, S., J., Li, W., Chen, L. J., Fong, C., Huang, Y., Feng, et al. 2025. “Extreme Heterochiasmy and High Rates of Sex-Reversed Recombination Result in Large Yet Homomorphic Sex Chromosomes in the Emei Moustache Toad”. *Genome Research* **35**, no. 6: 1325-1336.

Xu, Z., and H. Wang. 2007. “LTR_FINDER: An Efficient Tool for the Prediction of Full-Length LTR Retrotransposons.” *Nucleic Acids Research* **35**, no. Suppl 2: W265–W268.

Yu, H., C. Shi, W. He, F. Li, and B. Ouyang. 2024. “PanDepth, an Ultrafast and Efficient Genomic Tool for Coverage Calculation.” *Briefings in Bioinformatics* **25**, no. 3: bbae197.

Zdobnov, E. M., and R. Apweiler. 2001. “InterProScan -An Integration Platform for the Signature-Recognition Methods in InterPro.” *Bioinformatics* **17**, no. 9: 847-848.

## SUPPLEMENTARY FIGURES

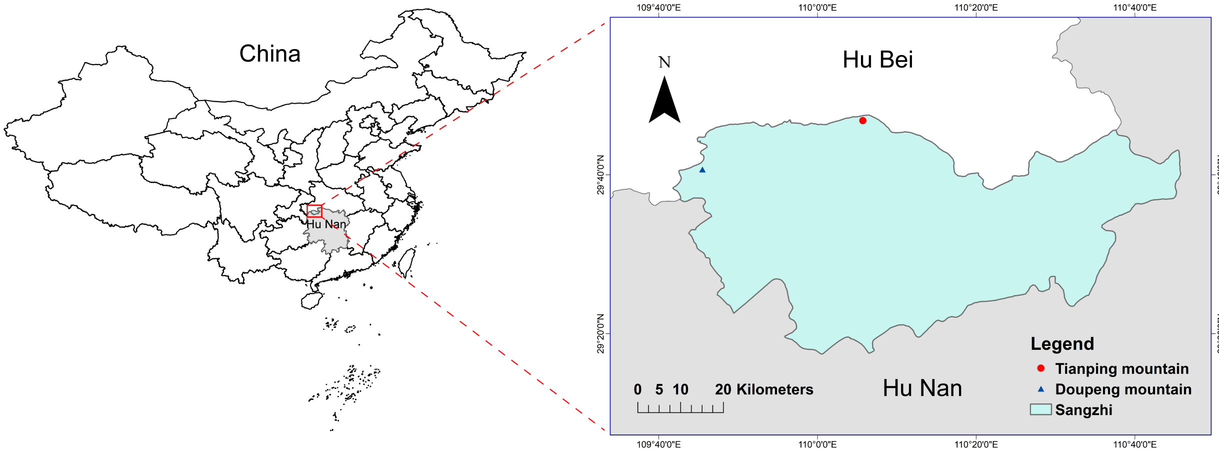


**Figure S1**. Geographic location of the sampling sites of *B. sangzhiensis* in the Badagong Mountain National Nature Reserve, Hunan Province, China. The right panel shows two specific collection sites: Tiangping Mountain and Doupeng Mountain within the Sangzhi county. These localities represent the distribution range of *B. sangzhiensis* used for genomic and transcriptomic sampling.

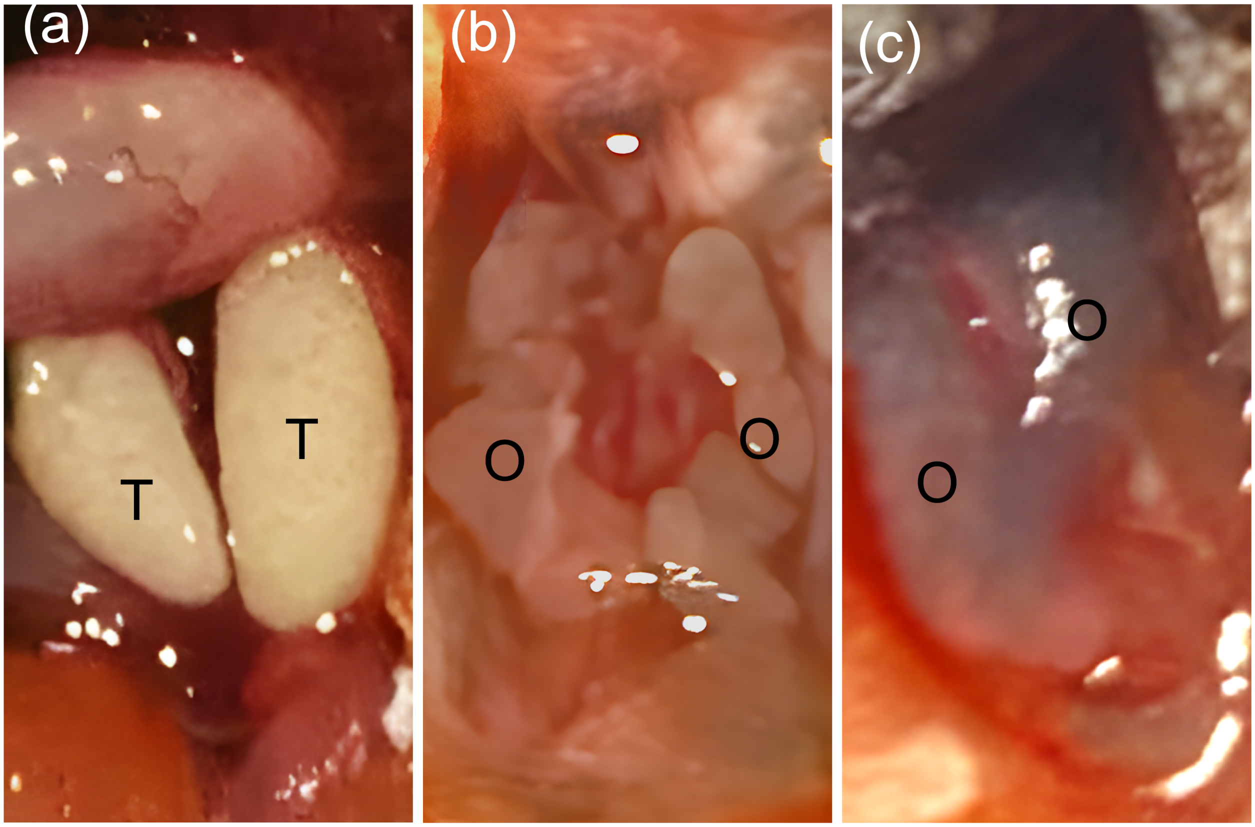


**Figure S2**. Gonadal tissues of sexually mature male (a) and subadult female individuals at different developmental stages (b, c). T indicates the testes, and O indicates the ovaries. All individuals were derived from the resequencing dataset used in this study.

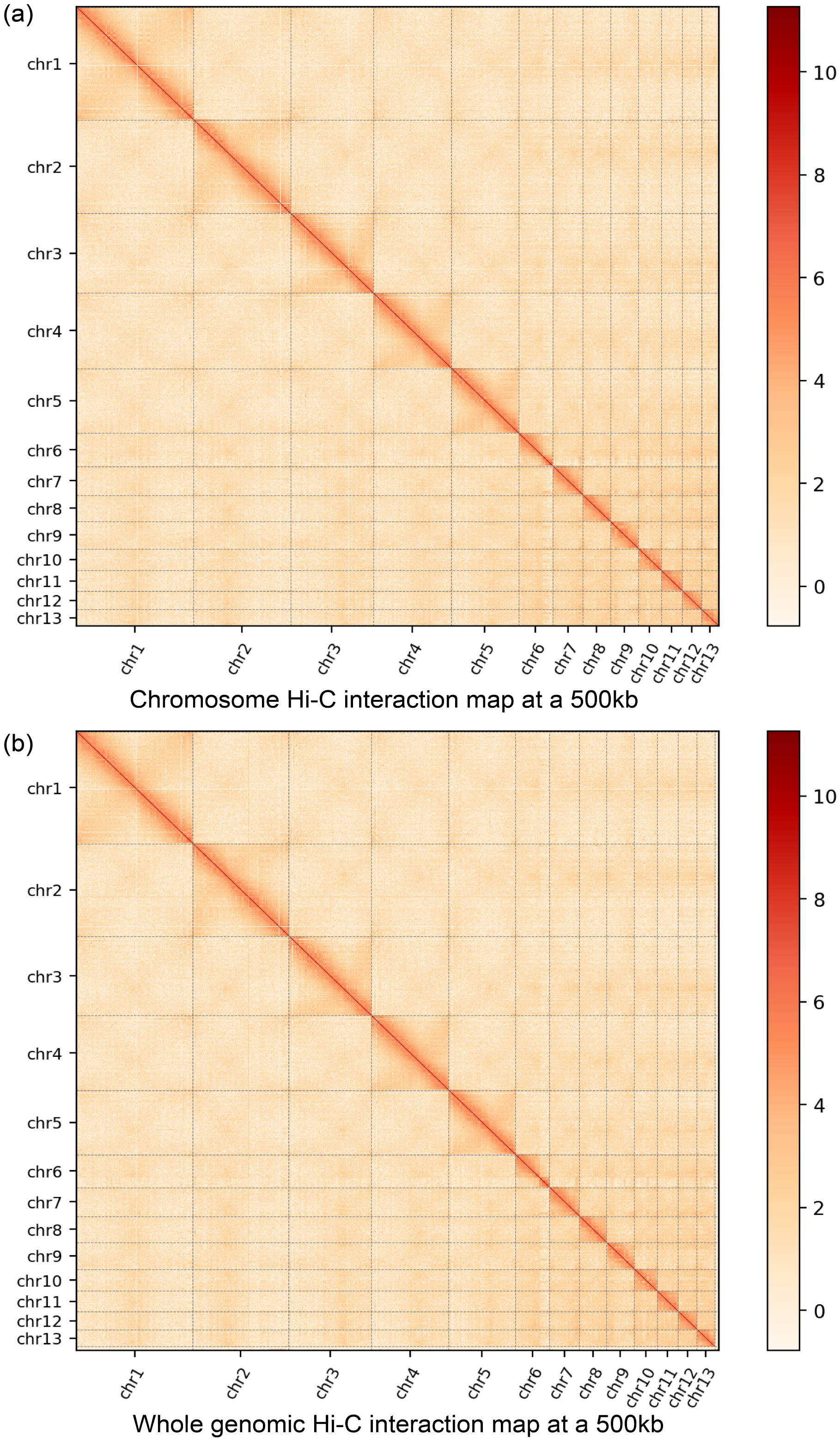


**Figure S3**. The chromosome (a) and whole-genome (b) Hi-C interaction maps of *B. sangzhiensis* at 500kb resolution. The color ranging from light to dark indicate increasing contact frequency between genomic loci, with darker shades indicating stronger interactions. Hi-C contact maps showed 13 strong interaction blocks corresponding to the 13 chromosomes of *B. sangzhiensis*.

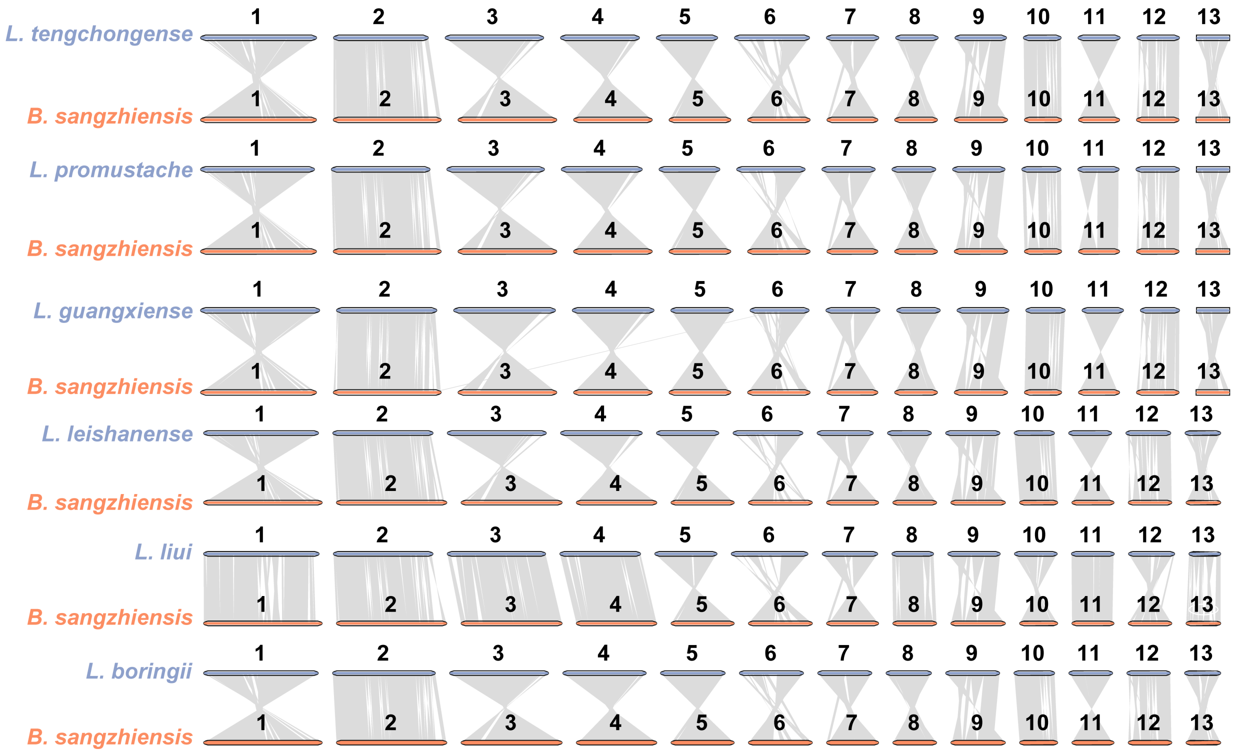


**Figure S4.** Whole-genome macrosyntenic relationships between *B. sangzhiensis* and its closely related species (*L. tengchongense*, *L. promustache*, *L. guangxiense*, *L. leishanense*, *L. liui*, and *L. boringii*). Each line connects orthologous genomic regions between homologous chromosomes. Chr 6 of *B. sangzhiensis* shows a clear one-to-one correspondence with Chr 6 of its relatives, with no evidence of interchromosomal fusion or translocation, supporting the structural integrity of the *B. sangzhiensis* assembly.

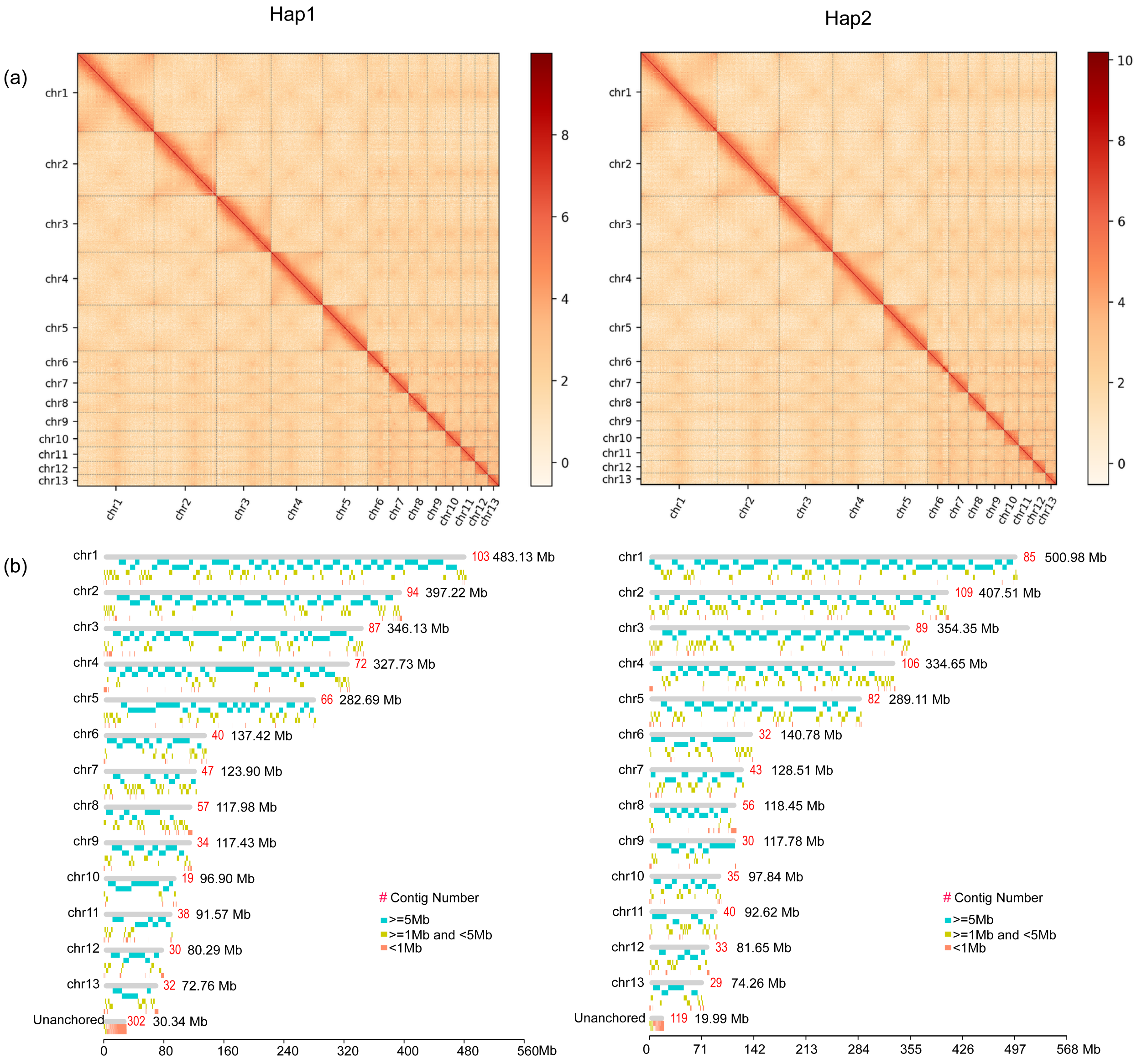


**Figure S5.** Chromosome anchoring and assembly integrity of *B. sangzhiensis* haplotypes. (a) Hi-C contact maps of haplotype 1 (Hap1) and haplotype 2 (Hap2) at 1-Mb resolution. The dense and continuous diagonal signals indicate high assembly continuity and accurate chromosome anchoring. (b) Distribution of contigs across chromosomes for Hap1 and Hap2. Grey bars represent chromosome length, while colored blocks represent contigs of different size ranges (<1 Mb, 1–5 Mb, >5 Mb). Most chromosomes are anchored by a few large contigs, confirming high contiguity of the assembly.

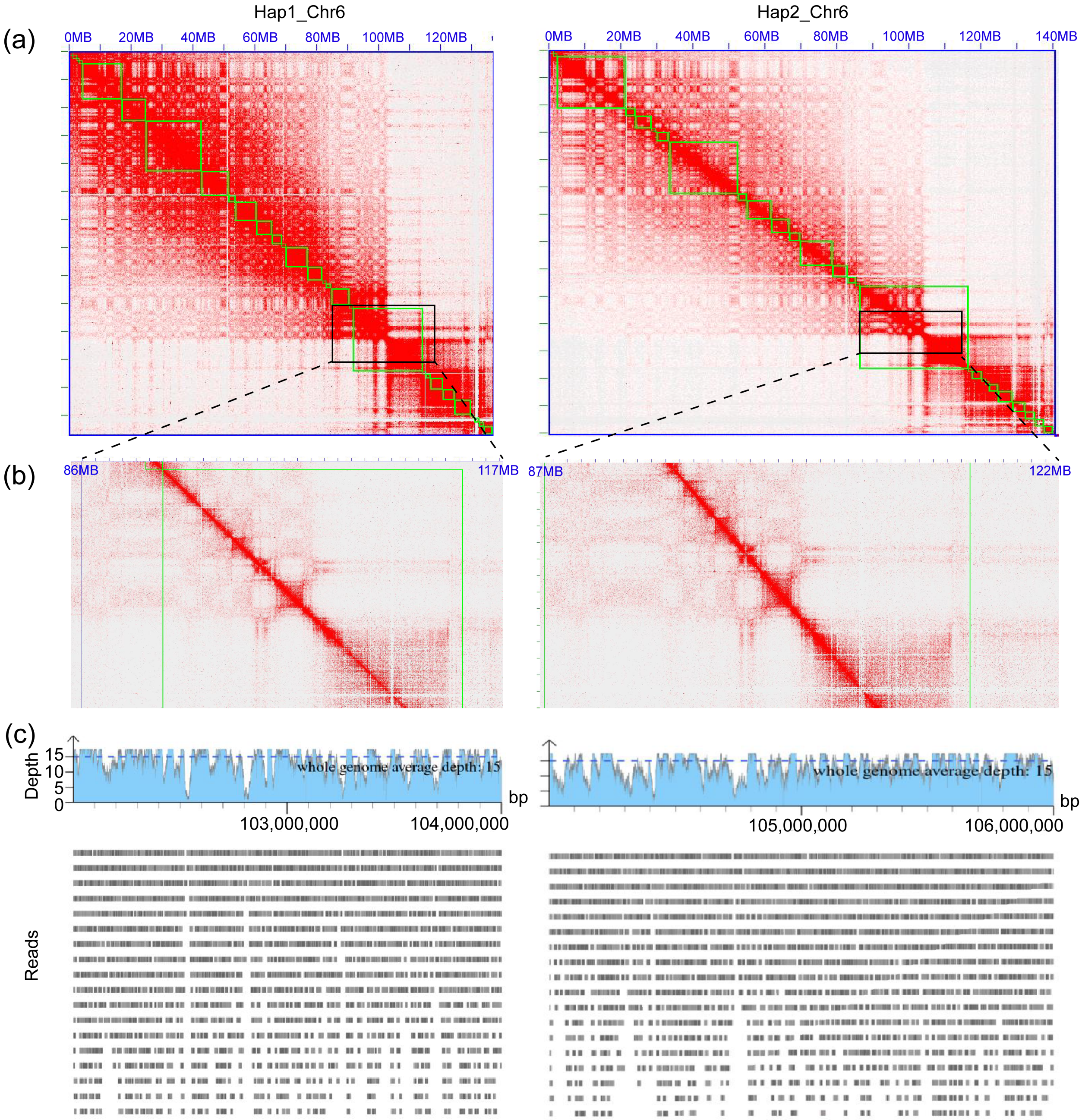


**Figure S6.** Validation of the assembly integrity on Chr 6 in *B. sangzhiensis*. (a) Hi-C contact heatmaps of Chr 6 for haplotype 1 (Hap1) and haplotype 2 (Hap2). The x- and y-axes represent chromosomal coordinates. (b) Local magnification of the Hi-C contact maps showing continuous contact patterns near the putative breakpoints. Juicebox visualization confirmed that Chr 6 is represented by a single contig, with contact breakpoints located at ~103.2 Mb on Hap1 and ~104.7 Mb on Hap2. (c) Mapping depth and read alignment patterns near the corresponding breakpoint regions. PacBio HiFi reads were mapped to the merged haplotype assembly using Minimap2 (v2.24, -ax map-hifi). The presence of numerous reads spanning the breakpoint regions indicates the absence of assembly gaps or structural misjoins.

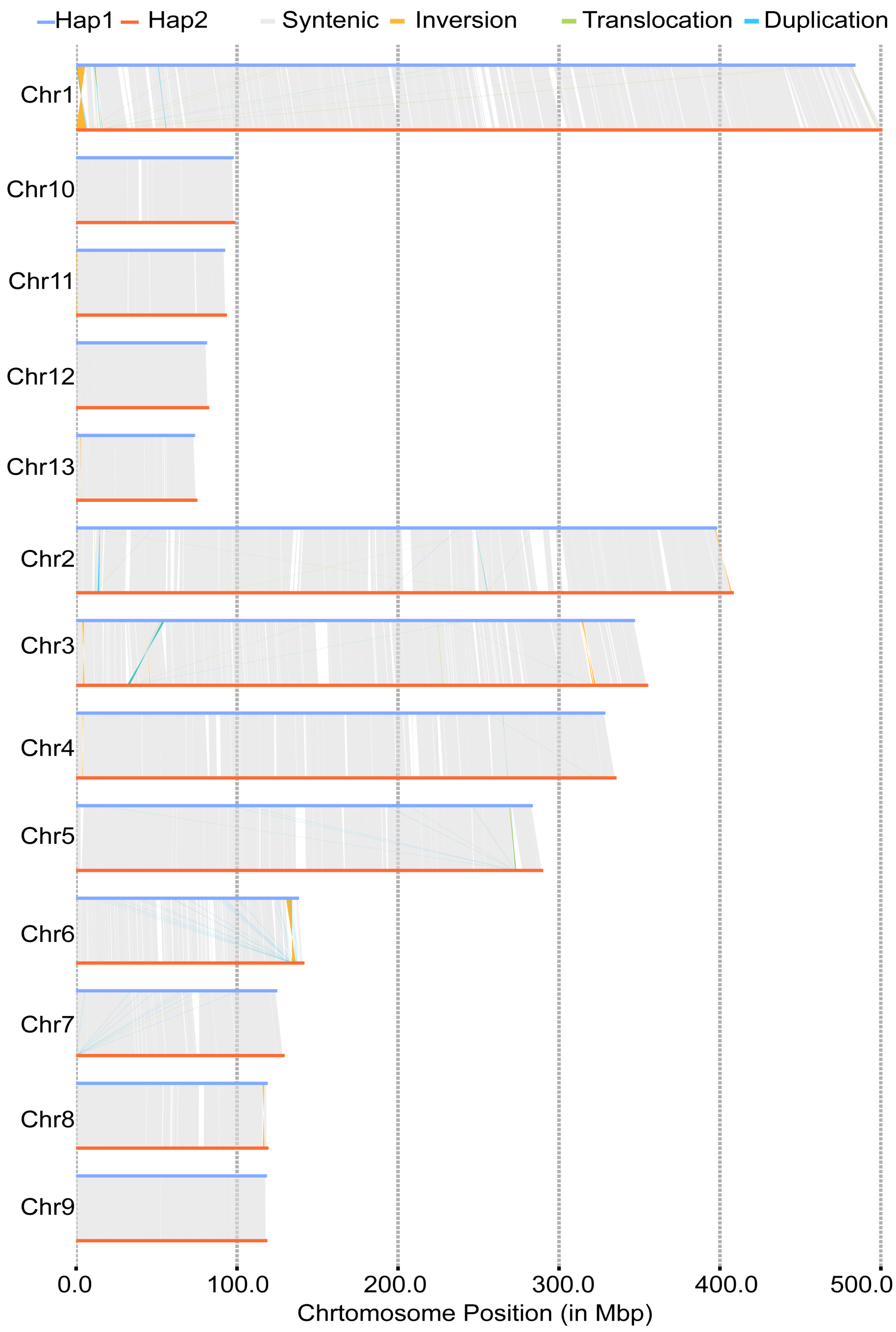


**Figure S7**. Chromosomal collinearity and structural variation between the two pseudo-haplotypes (Hap1 and Hap2) of *B. sangzhiensis*. Syntenic relationships and large-scale structural variants were identified by whole-genome alignment using Syri. Hap1 and Hap2 assemblies are shown in blue and orange, respectively. Gray lines indicate collinear (syntenic) regions, while structural rearrangements are highlighted as follows: orange lines represent inversions, green lines represent translocations, and cyan lines represent duplications. Extensive collinearity is observed across most chromosomes, with several localized rearrangements detected—particularly on Chr 6, suggesting potential intra-chromosomal duplication and inversion events within the sex-linked region.

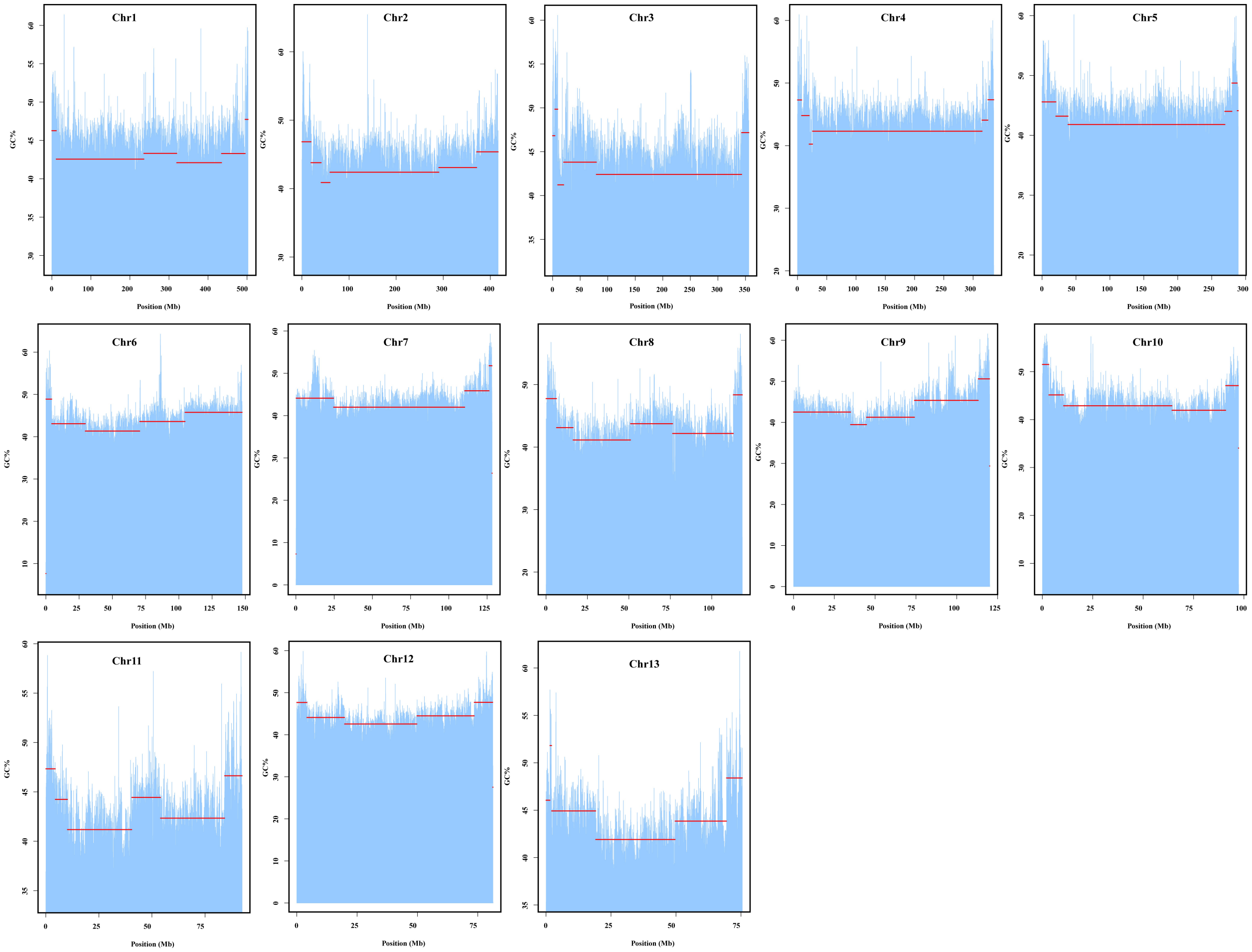


**Figure S8.** Change-point analysis of GC content of *B. sangzhiensis*. Each horizontal line represents the mean GC% (window=50kb) of a segment identified by change-point analysis (Killick and Eckley 2014). Commands for change-point analysis was cpt.mean(method = "BinSeg"). We can observed that GC content varied between 40–60% across chromosomes, with the sex-associated region on Chr 2 and Chr 6 showing moderate GC content (40–50%), rather than the highest GC levels genome-wide.

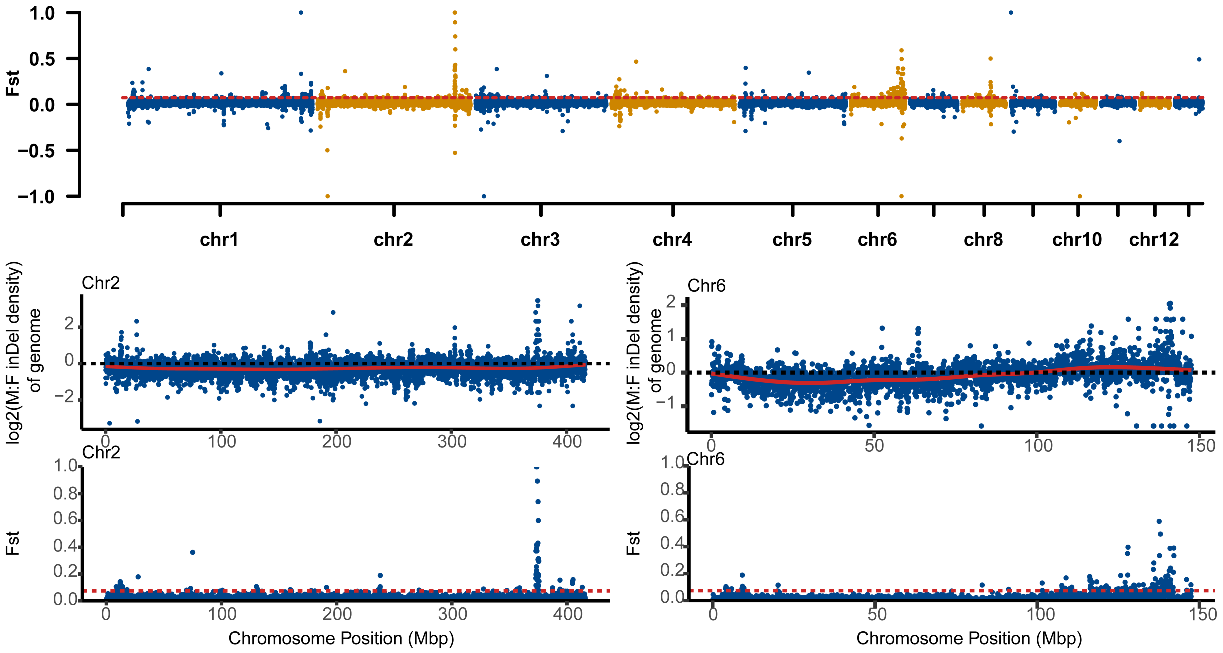


**Figure S9.** Genome-wide identification of sex-linked regions in *B. sangzhiensis* (window=50kb). (Top) Genome-wide distribution of Indel-based $F_{ST}$ values between 20 males and 20 females across all chromosomes. Orange and blue dots represent InDels with positive and negative $F_{ST}$ values, respectively. (Bottom) Detailed analysis of Chr 2 and Chr 6. For each chromosome, plots show the $log_2$(M:F) InDel density (top panels) and Indel-based $F_{ST}$ values (bottom panels) calculated in non-overlapping windows.

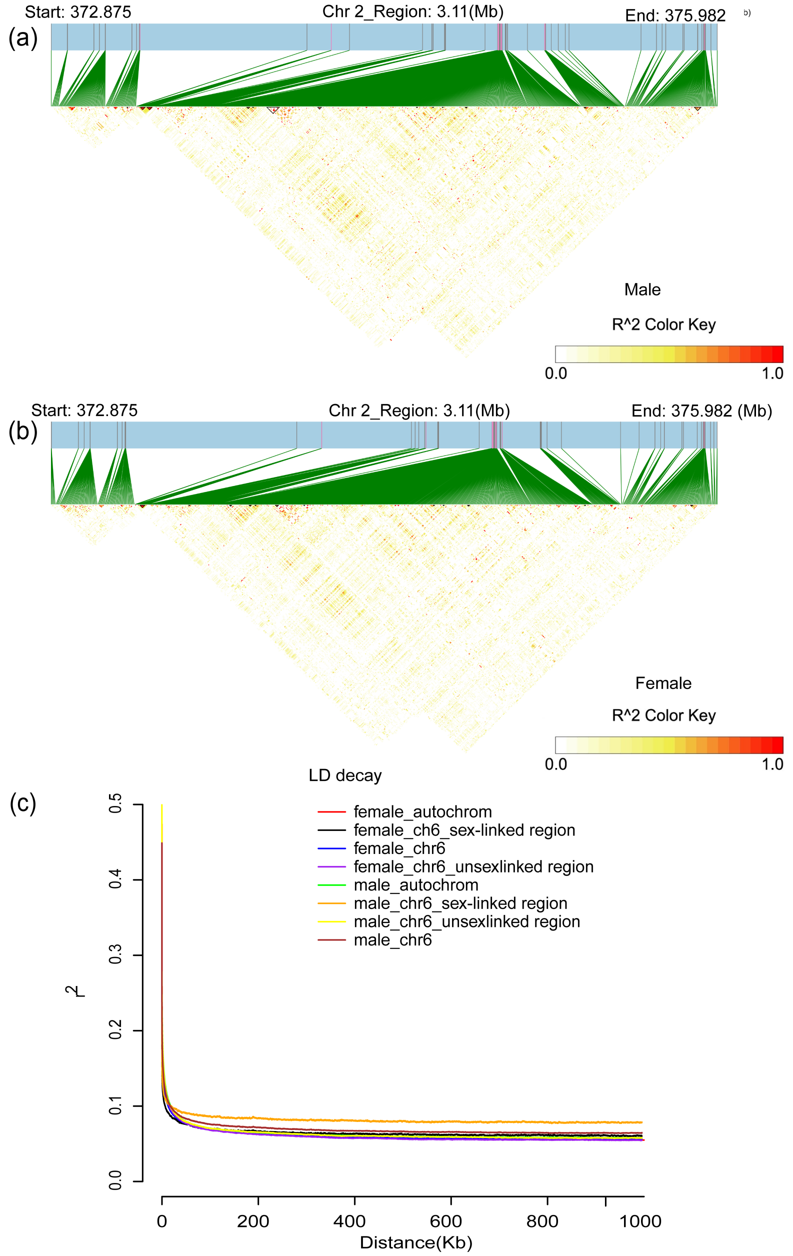


**Figure S10.** Heat map of linkage disequilibrium (LD) in SLR on Chr 2 from 20 males (a) and 20 females (b), showing high $R^2$ values. (c) LD decay analysis showing the relationship between distance (kb) and LD ($R^2$) on Chr 6 in 20 males (a) and 20 females. Different colors represent male and female chromosomes, highlighting sex-specific LD decay patterns. LD patterns in *B. sangzhiensis* based on variant data from 20 females and 20 males.

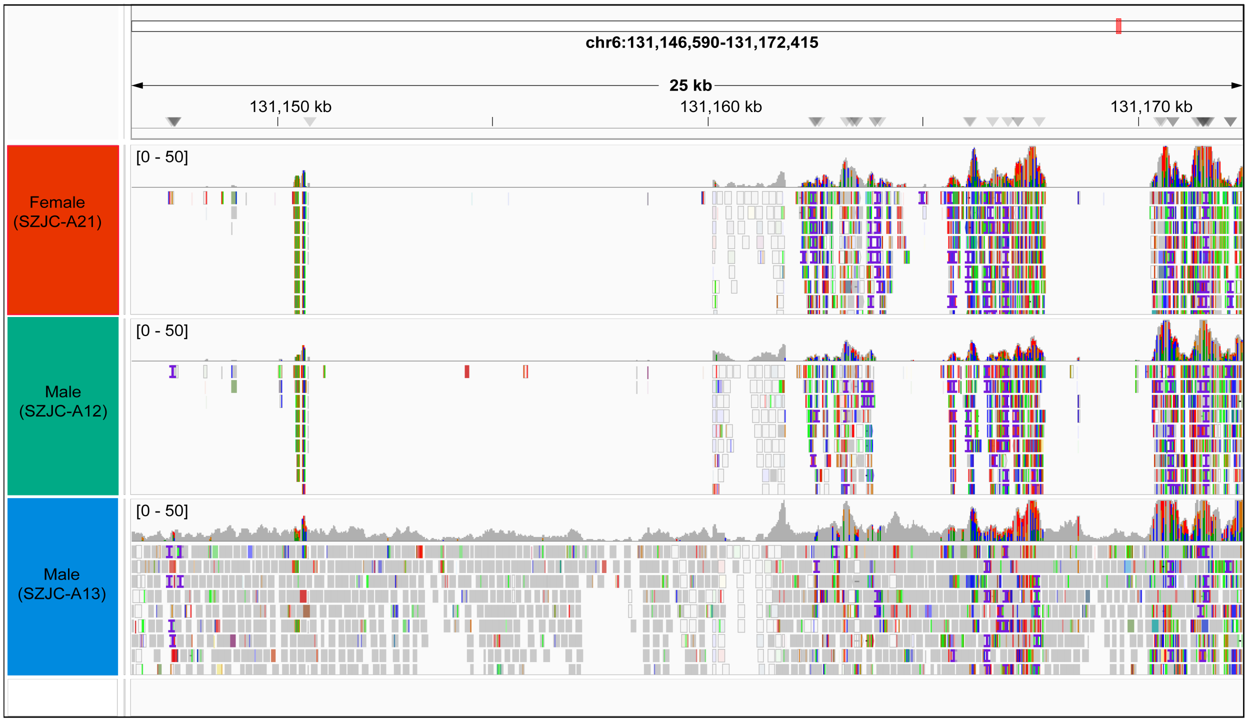


**Figure S11.** Integrative Genomics Viewer (IGV) screenshot showing the sex-specific coverage bias in the putative sex-linked region on Chr 6 of *B. sangzhiensis*. The coverage tracks display for both males and females the read depth (scale 0-50 reads). This region of Chr 6 corresponds to 131,146,590-131,172,415bp. Red, green and blue track represents female, M1 and M2, respectively.

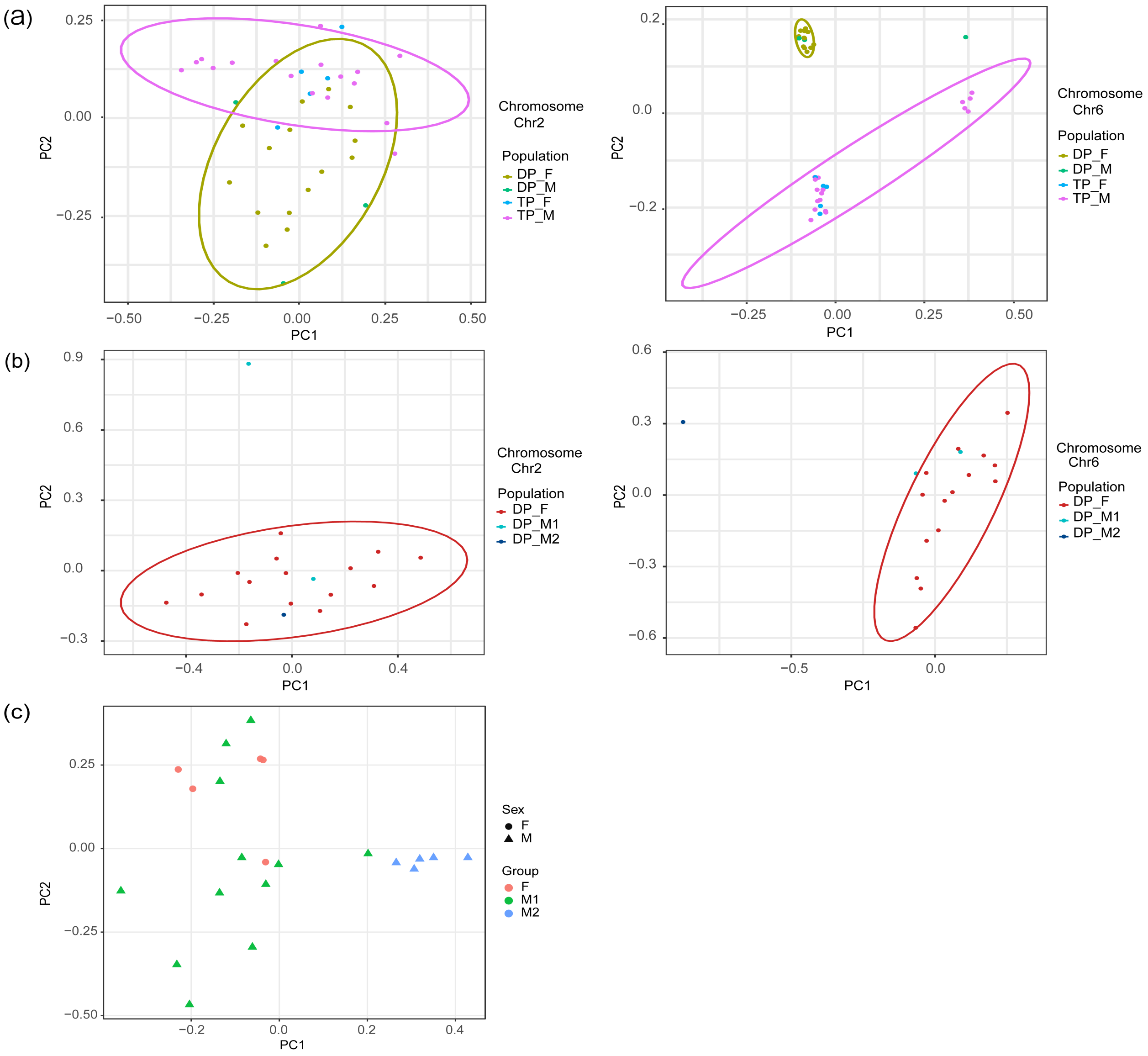


**Figure S12.** (a) PCA plot for SNPs in sex-linked region on Chr 2 and Chr 6 in two populations: Tianping Mountain (TP) and Doupeng Mountain (DP). (b) PCA plot for SNPs in sex-linked region on Chr 2 and Chr 6 in DP population. (c) PC1–PC2 plot for SNPs in sex-linked region on Chr 2 in TP population.

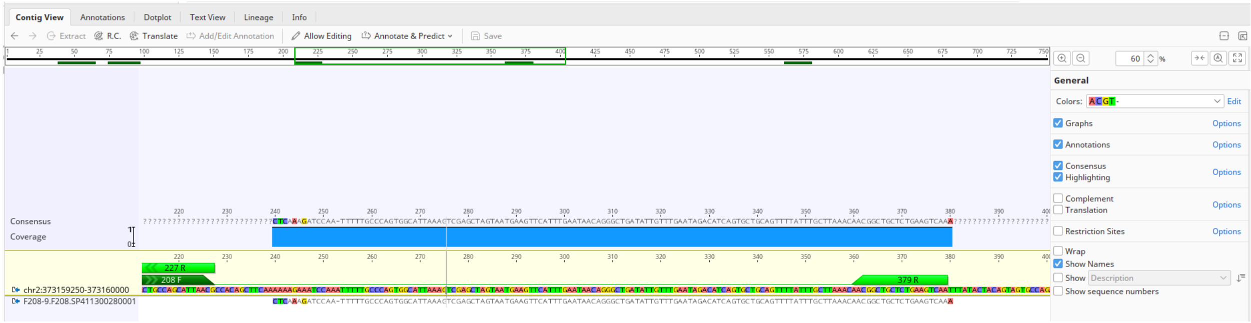


**Figure S13.** Mapping of Sanger sequencing PCR product to the genome region using Geneious software. The image shows the alignment of Sanger sequencing data (bottom) to the reference genome (top) within the Geneious interface. The green bars represent the aligned regions, with coverage indicated by the blue shading. This visualization demonstrates the successful mapping of the PCR product to the target genomic region, providing confirmation of sequence identity and quality. The alignment also shows the detailed nucleotide sequence at the mapped location.

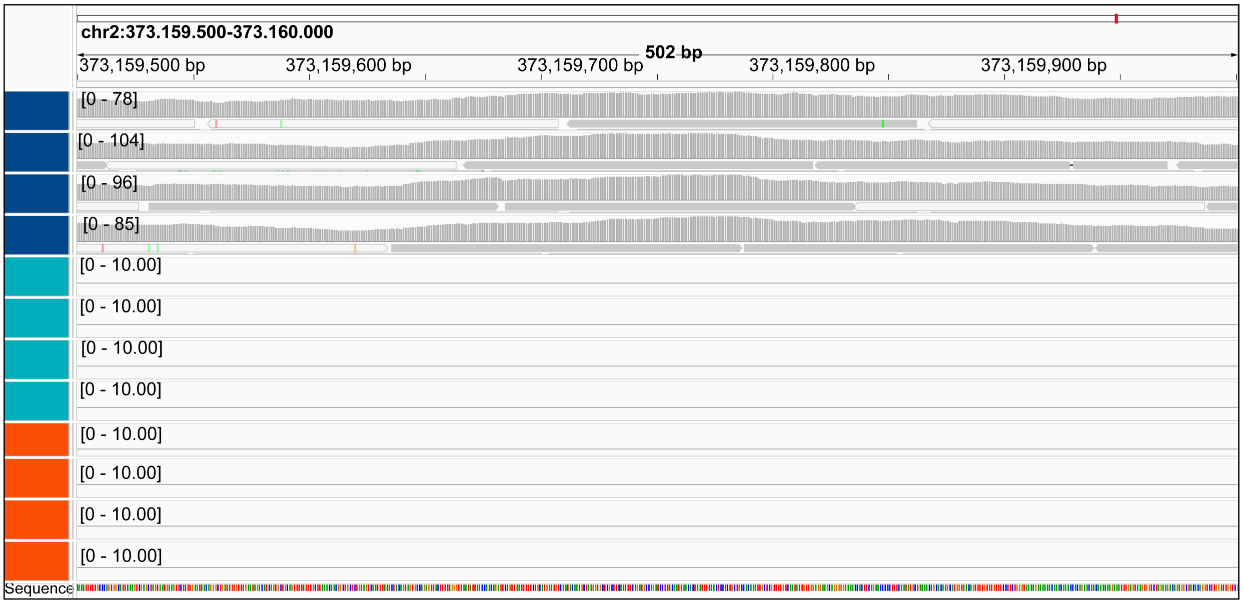


**Figure S14.** Visualization of the sex -specific coverage in *B. sangzhiensis* using IGV. 12 individuals were randomly selected. Blue indicating male phenotypic sex and red indicating female phenotypic sex. Dark blue represents normal males, whose genotype and phenotype are both male, showing coverage in these regions. Light blue-green represents sex-reversed individuals, which, similar to red-colored genotypic females, show no coverage.

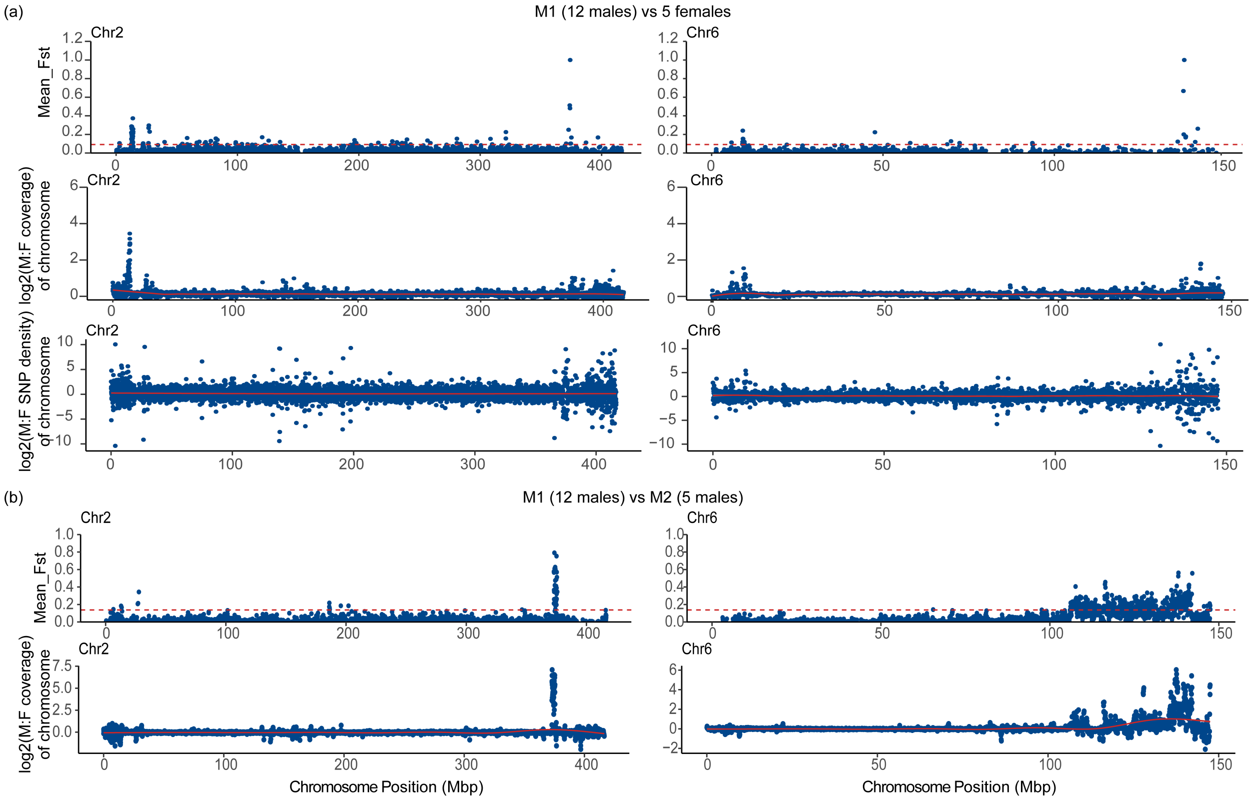


**Figure S15.** Genomic differentiation between M1 (12 males) and 5 females (a) and M1 and M2 (b) in *B. sangzhiensis* from the Tianping Mountain population. Each panel shows the mean $F_{ST}$ based on SNP, $\log_2$(M:F) coverage ratio, and SNP density calculated in 50-kb sliding windows. The dashed red line marks the top 1% $F_{ST}$ threshold used to define regions of significant genetic differentiation between groups. Peaks in $F_{ST}$, male-biased coverage, and male-biased SNP density on Chr 2 and Chr 6 highlight putative sex-linked regions.

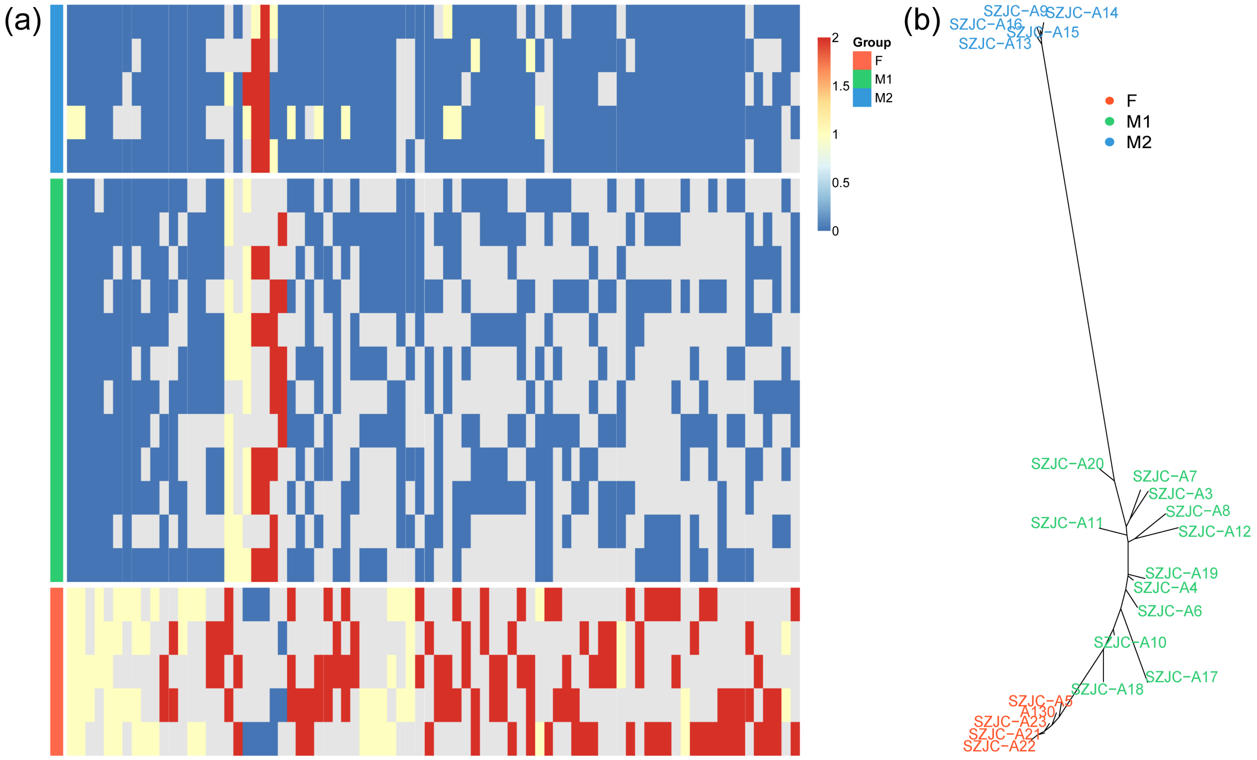


**Figure S16**. (a) Genotype heatmap based on filtered informative loci from the top strict differentiated loci in the F vs M1 comparison within the Chr 6 SLR of *B. sangzhiensis* from the TP population. Individuals are grouped as females (F), M1 males, and M2 males. Genotypes are coded as 0/0, 0/1, and 1/1, with missing data shown in grey. (b) Neighbor-joining (NJ) tree was construct based on the F vs M2 relaxed loci and F vs M1 strict loci Chr 6 in SLR on Chr 6 using ape package. M2 forms the most strongly differentiated male-like cluster, females form a separate cluster, and M1 occupies an intermediate position. These results are consistent with weak but detectable residual Chr 6-linked differentiation in M1 males.

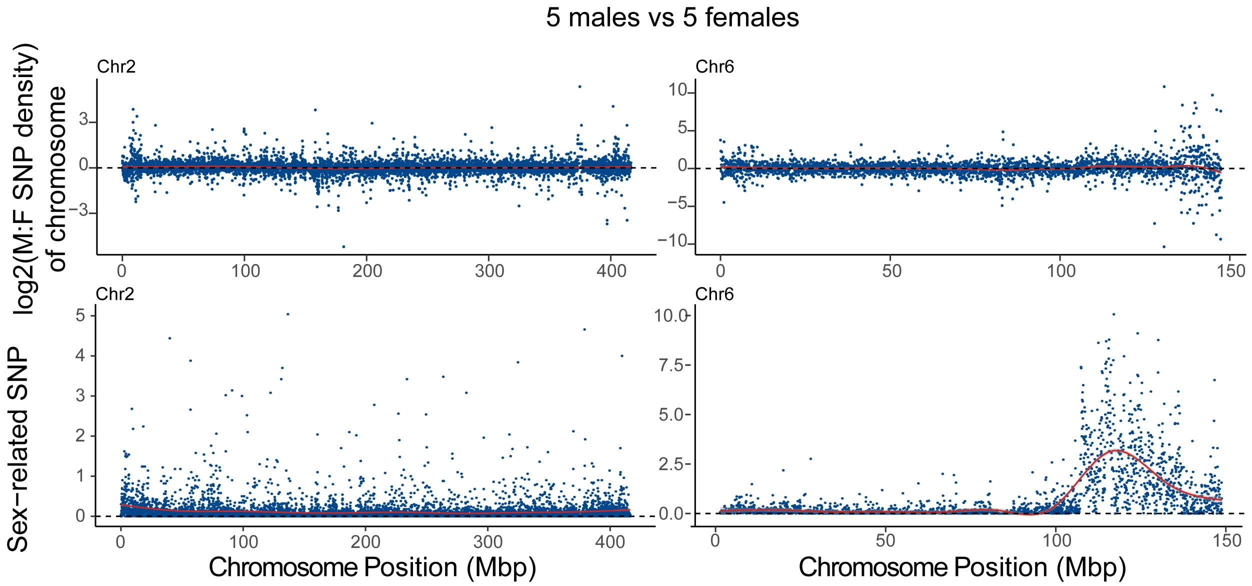


**Figure S17.** Sex-linked signal detection on Chr 2 and Chr 6 in *B. sangzhiensis* (5 M2 males vs. 5 females) from the Tianping Mountain population (window size = 50 kb). Upper panels: $\log_2$(M:F) ratio of SNP density across Chr 2 and Chr 6, showing sex-biased SNP distribution. Lower panels: number of sex-related SNPs along Chr 2 and Chr 6.

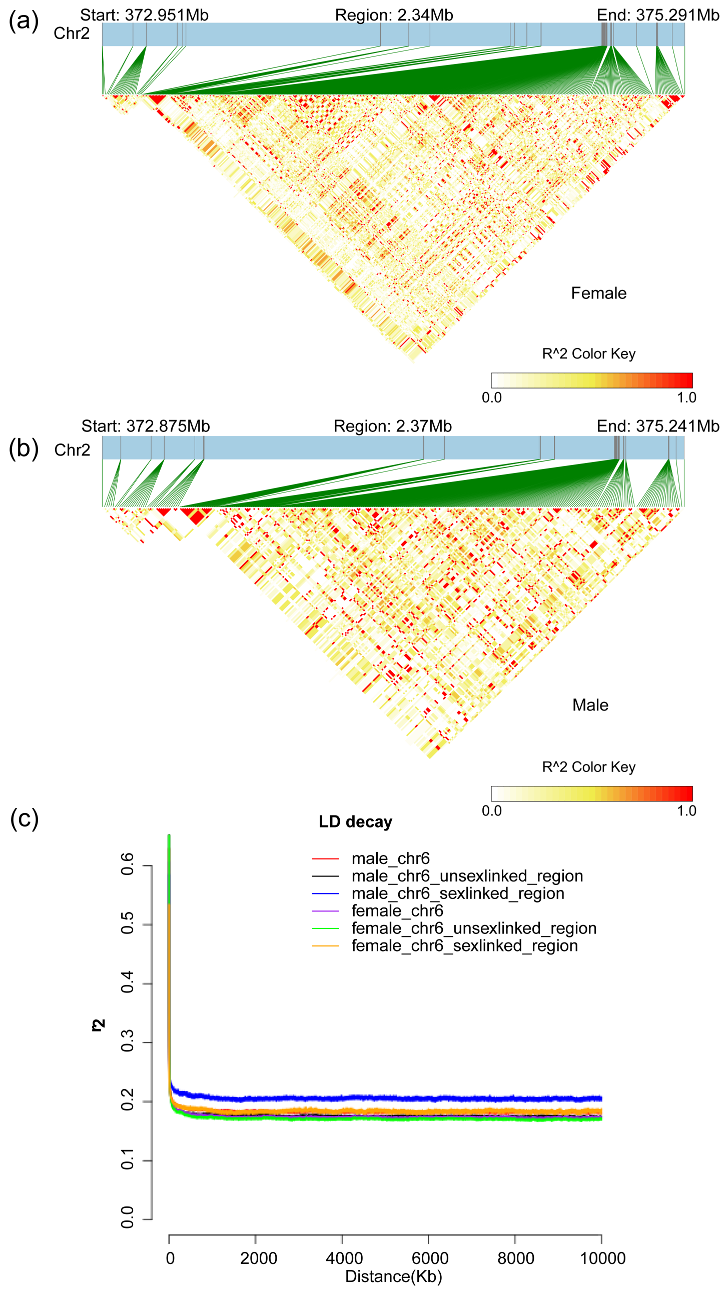


**Figure S18.** Heat map of linkage disequilibrium (LD) in SLR on Chr 2 in 5 males (a) and 5 females (b), showing high $R^2$ values. (c) LD decay analysis showing the relationship between distance (kb) and LD ($R^2$) on Chr 6. Different colors represent male and female chromosomes, highlighting sex-specific LD decay patterns. LD patterns in *B. sangzhiensis* based on variant data from 5 females and 5 males (M2) in Tianping Mountain.

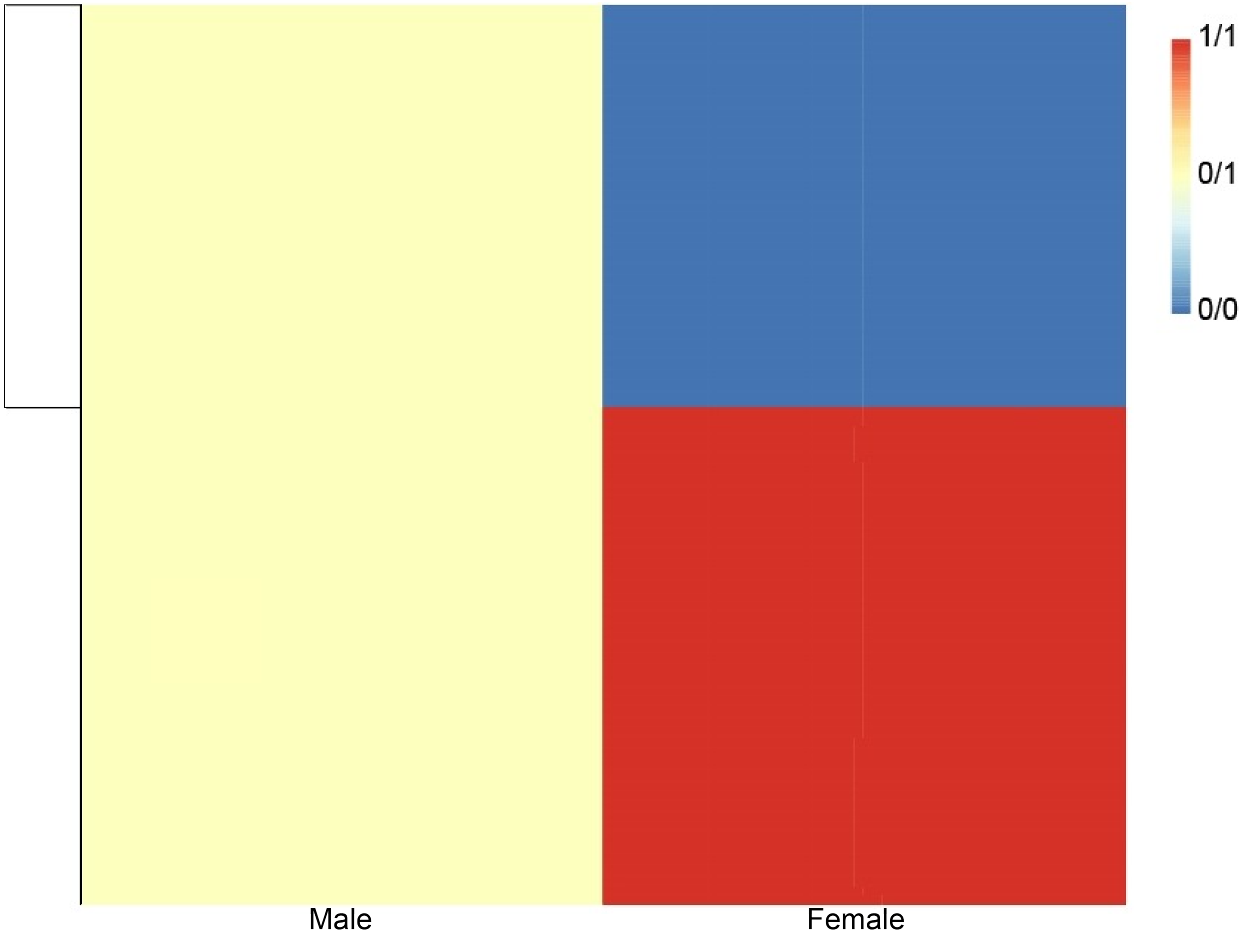


**Figure S19.** Genotype heatmap of the sex-linked region on Chr 6 in *B. sangzhiensis*, based on five female and five male individuals (M2). Each column represents an individual, and each row represents a SNP within the sex-linked region. Colors indicate genotypes: homozygous reference (0/0, blue), heterozygous (0/1, yellow), and homozygous alternate (1/1, red). The consistent genotype segregation between sexes highlights a strongly sex-associated haplotype block on Chr 6.

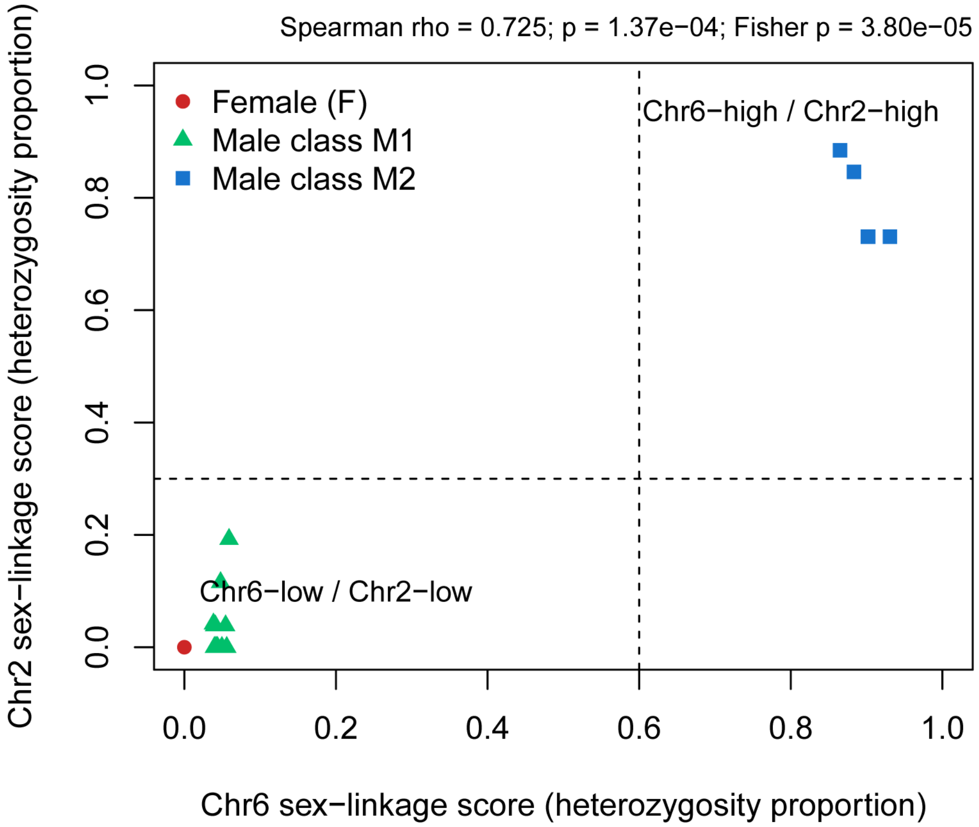


**Figure S20**. Individual-level concordance between sex-linkage signals on Chr 6 and Chr 2 in the TP population. Each point represents one TP individual (5 females, 12 M1 males, 5 M2 males). XY-like loci were defined a priori using the F vs M2 contrast (5 females vs 5 M2 males) as sites where all called females were fixed for the same homozygous genotype (all 0/0 or all 1/1) together with enrichment of male heterozygosity (0/1), a criterion invariant to REF/ALT assignment. Using this fixed marker panel (Chr 6: 70,924 loci; Chr 2: 26 loci), we computed per-individual sex-linkage scores as the proportion of heterozygous genotypes (0/1) across XY-like loci separately for Chr 6 (x-axis) and Chr 2 (y-axis). Dashed lines indicate the predefined thresholds used to dichotomize scores for the co-occurrence test (Chr 6 $\geqslant$ 0.6; Chr 2 $\geqslant$ 0.3), separating individuals into “Chr6-high/Chr2-high” and “Chr6-low/Chr2-low” quadrants. Scores clearly separated females (Chr 6 mean = 0; Chr 2 mean = 0), M1 males (Chr 6 mean = 0.046; Chr 2 mean = 0.039), and M2 males (Chr 6 mean = 0.897; Chr 2 mean = 0.785). M2 males cluster in the Chr6-high/Chr2-high quadrant, whereas females and M1 males cluster in the Chr6-low/Chr2-low quadrant, consistent with strong individual-level concordance of male-like signatures between Chr 6 and Chr 2 in TP (Spearman's ρ = 0.725; $p = 1.37 \times 10^{-4}$; Fisher's exact test $p = 3.80 \times 10^{-5}$; no discordant individuals). DP results are not shown here because male sampling was limited (3 males; M2 n = 1).

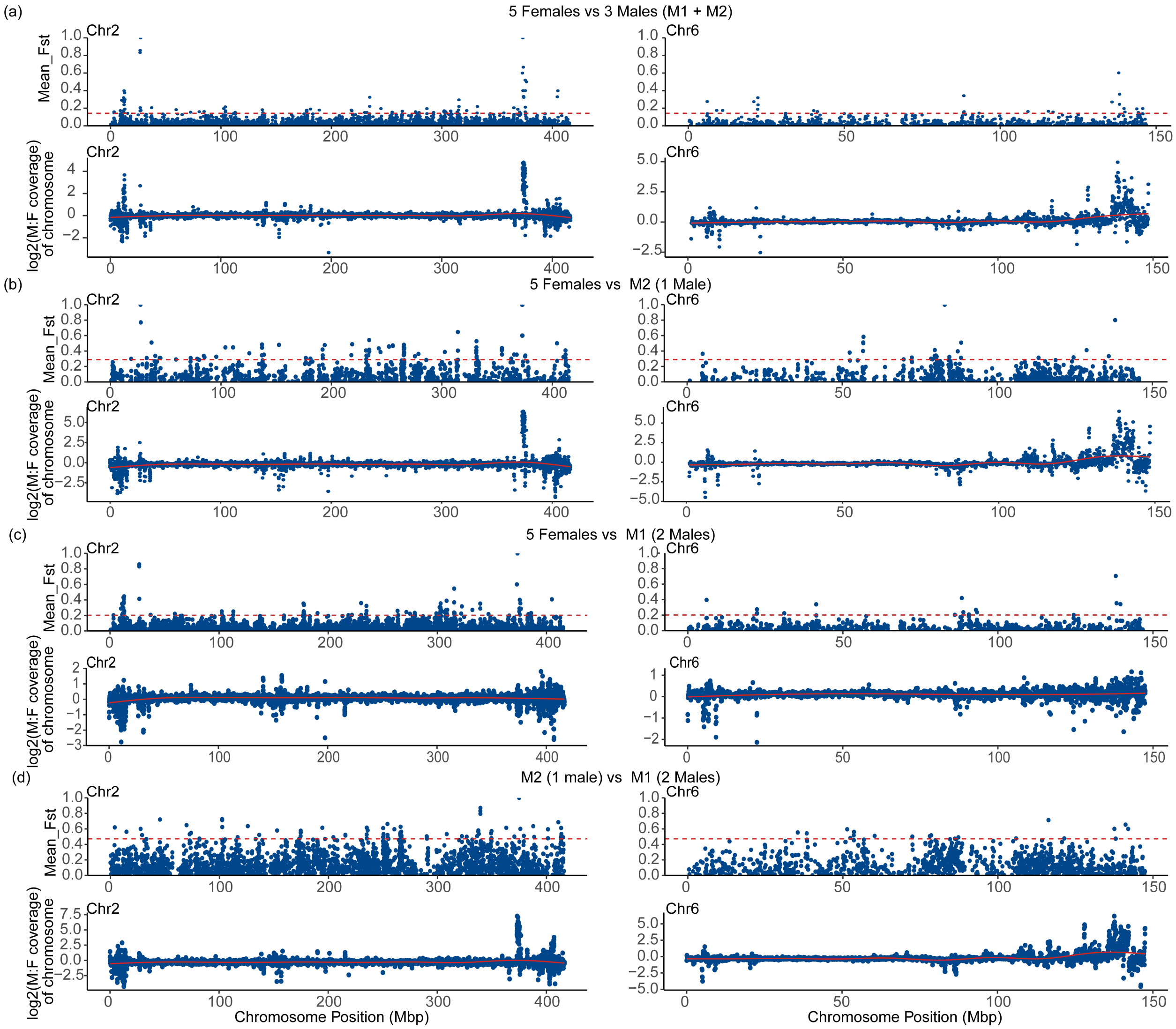


**Figure S21.** Genomic differentiation between all males and 5 females (a), M2 and 5 females (b), M1 and 5 females (c) and M1 and M2 (d) in the DP population. Each panel shows the mean $F_{ST}$ based on SNP and $\log_2$(M:F) coverage ratio calculated in 50-kb windows. The dashed red line marks the top 1% $F_{ST}$ threshold used to define regions of significant genetic differentiation between groups.

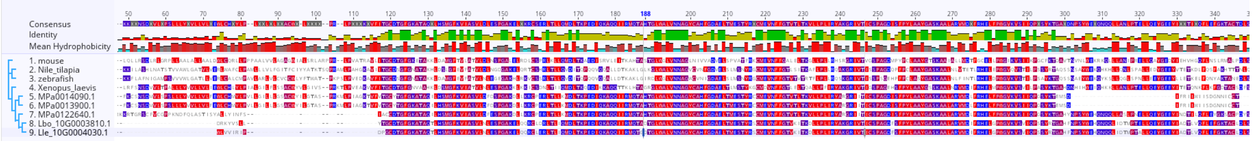


**Figure S22.** Phylogenetic analysis and multiple sequence alignments of *Hsd11b2* gene in different species. The top panel displays the consensus sequence with corresponding hydrophobicity values represented by a color gradient. The colored bars beneath the consensus line indicate the hydrophobicity levels of individual amino acids across the sequence, with red representing hydrophobic residues, blue for hydrophilic residues, and green for neutral residues. This figure was used to compare *Hsd11b2* sequences within the sex-linked region of *B. sangzhiensis*, showing that although the gene is highly conserved, sequence differences exist among species and between gene copies within the same genome.

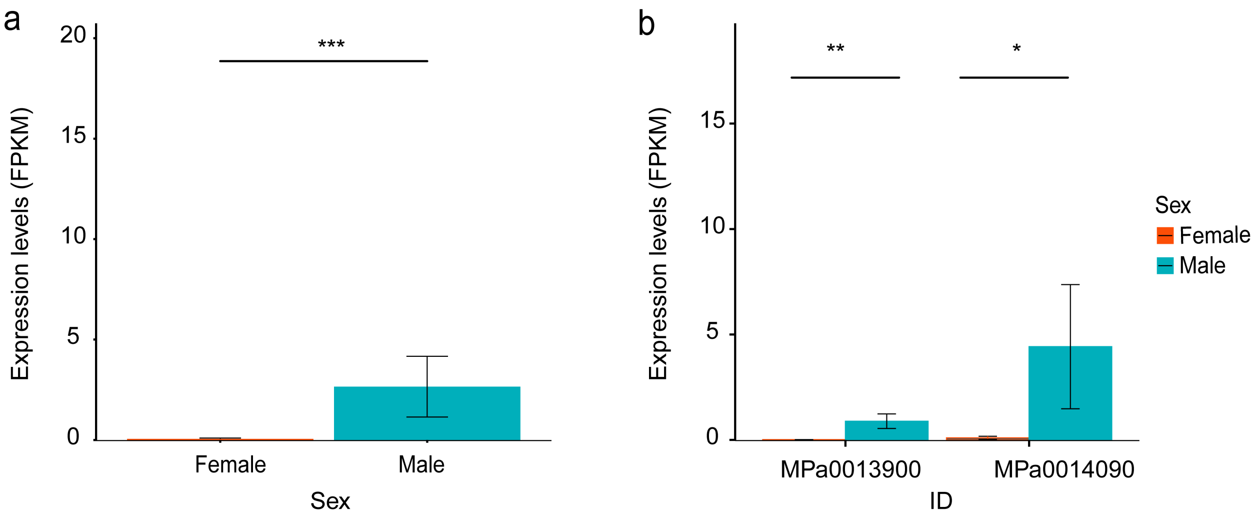


**Figure S23.** The expression level of *Hsd11b2* genes on Chr 2 in *B. sangzhiensis*. (a) Overall expression levels of *Hsd11b2* in males and females, showing significantly higher expression in males ($p < 0.001$) compared to females. (b) Expression levels of two *Hsd11b2* paralogs, MPa0013900 and MPa0014090, both exhibiting male-biased expression ($p < 0.01$, $p < 0.05$, respectively).

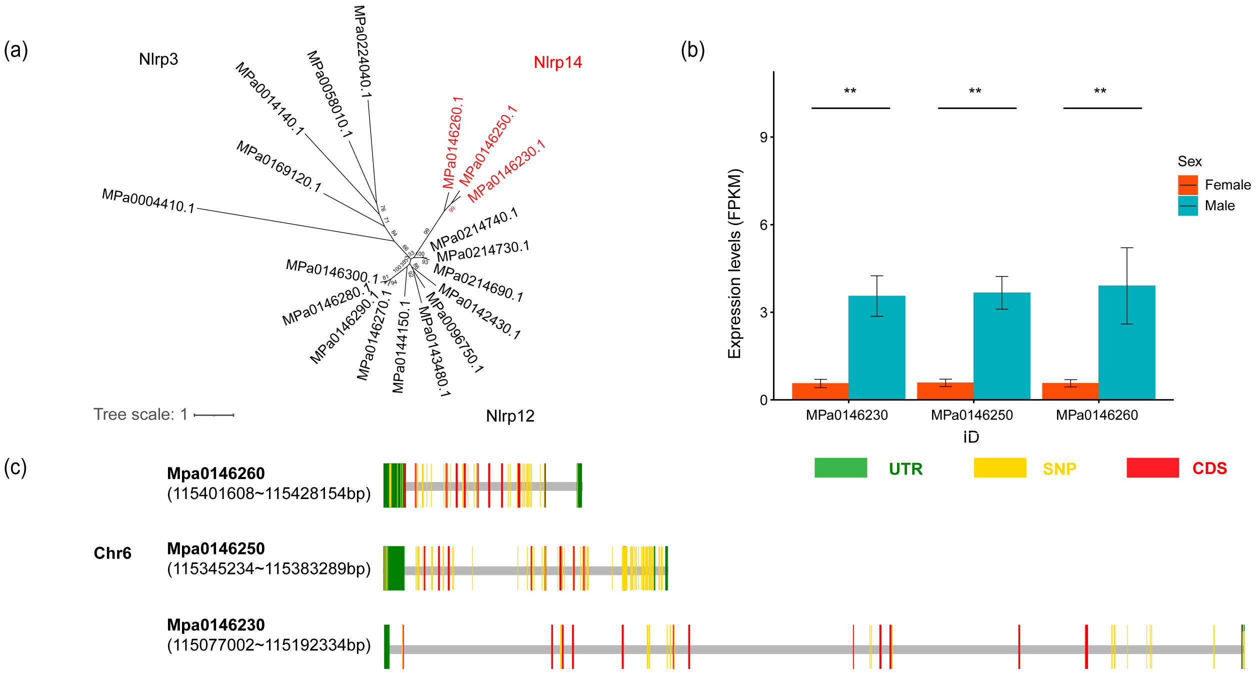


**Figure S24.** Characterize of *Nlrp14* gene in *B. sangzhiensis.* (a) sex-linked region harbored three copies of *Nlrp14* gene. (b) The expression level of *Nlrp14* genes. We could find that all three copies showed male-biased expression. (c) The gene structure of *Nlrp14*. SNP, single nucleotide polymorphism; CDS, coding sequences; UTR, untranslated region.

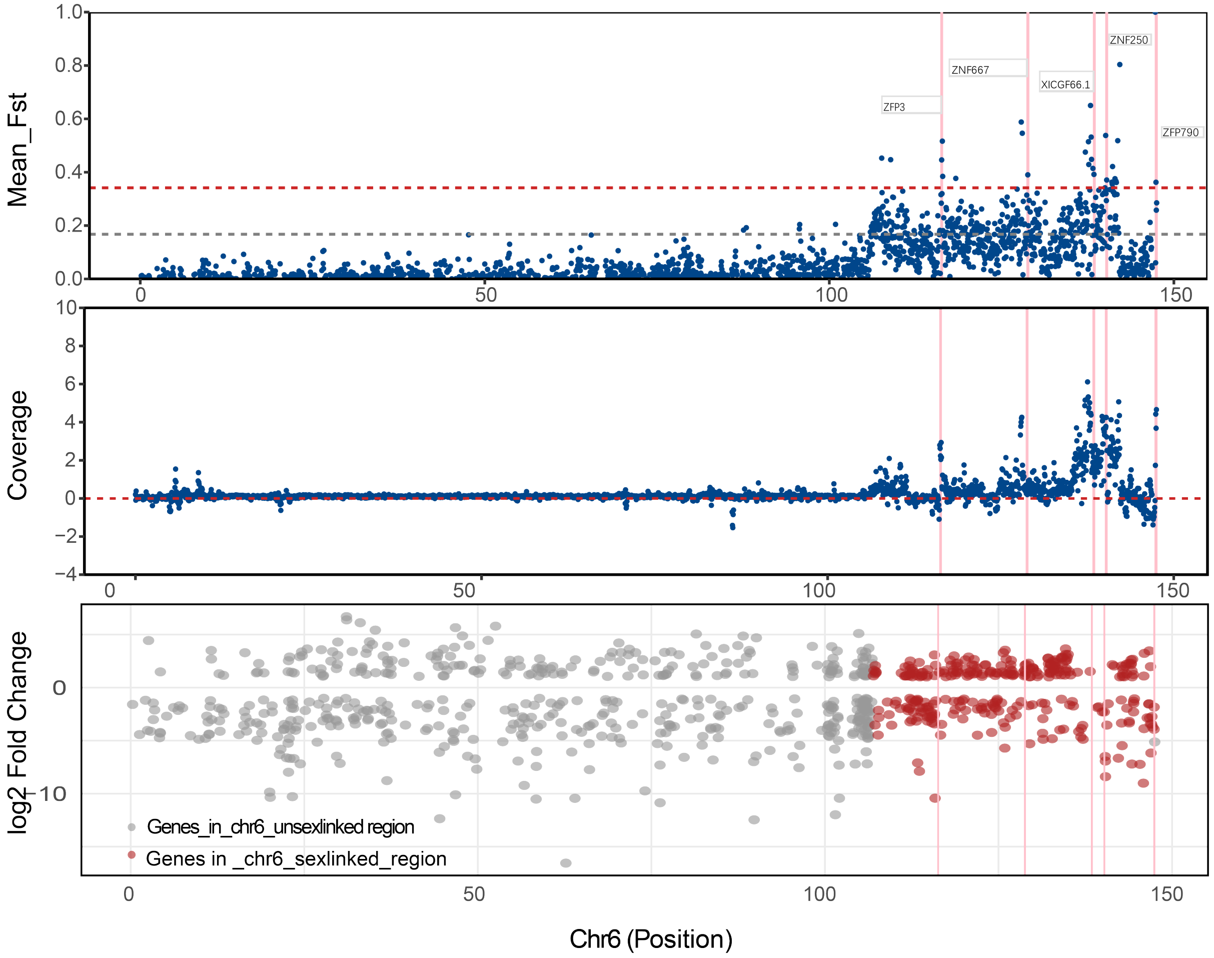


**Figure S25.** Characterize of candidate ZNF genes on Chr 6. The top track shows the mean $F_{ST}$ values across Chr 6 for 5 females and 5 males from Tianping Mountain, the red and gray horizontal lines represent the top 1% cutoff for significant divergence at the entire chromosome level and at Chr 6 between females and males, respectively. The middle track shows the male-to-female coverage ratio, calculated as log2 (male coverage + 0.01) - log2 (female coverage + 0.01). The pink vertical lines represent the distribution of the corresponding candidate ZNF gene. The bottom panel illustrates the distribution of differentially expressed genes (DEGs) along Chr 6 based on $\log_2$ fold change values. Red points denote DEGs located within the sex-linked region, while grey points represent DEGs in non–sex-linked regions. Negative $\log_2$ fold change values correspond to male-biased genes, and positive values indicate female-biased genes. The vertical pink lines mark the genomic positions of candidate *ZNF* genes (*ZNF667*, *ZFP30*, *ZFP3*, *ZFP250*, and *XlCGF66.1*), which are strongly associated with sex-linked differentiation.

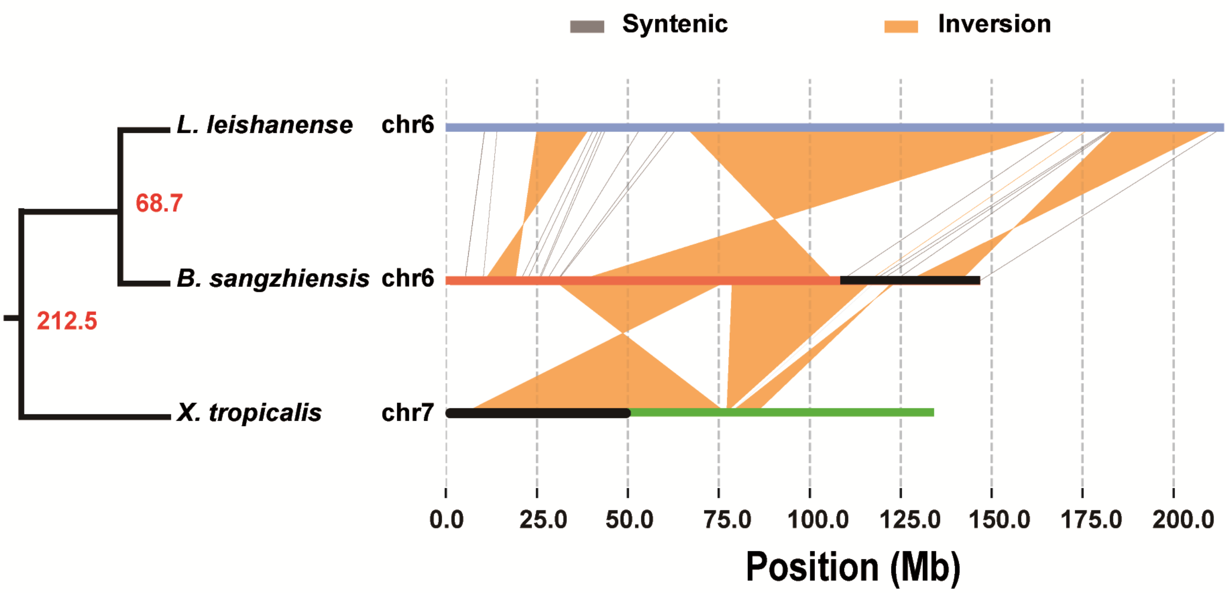


**Figure S26.** Chromosomal structural variants of *B. sangzhiensis*, *X. tropicalis* and *L. leishanense*. Black region on chromosome was the sex-linked region. left is the phylogenetic tree of the three species, with red numbers representing divergence times.

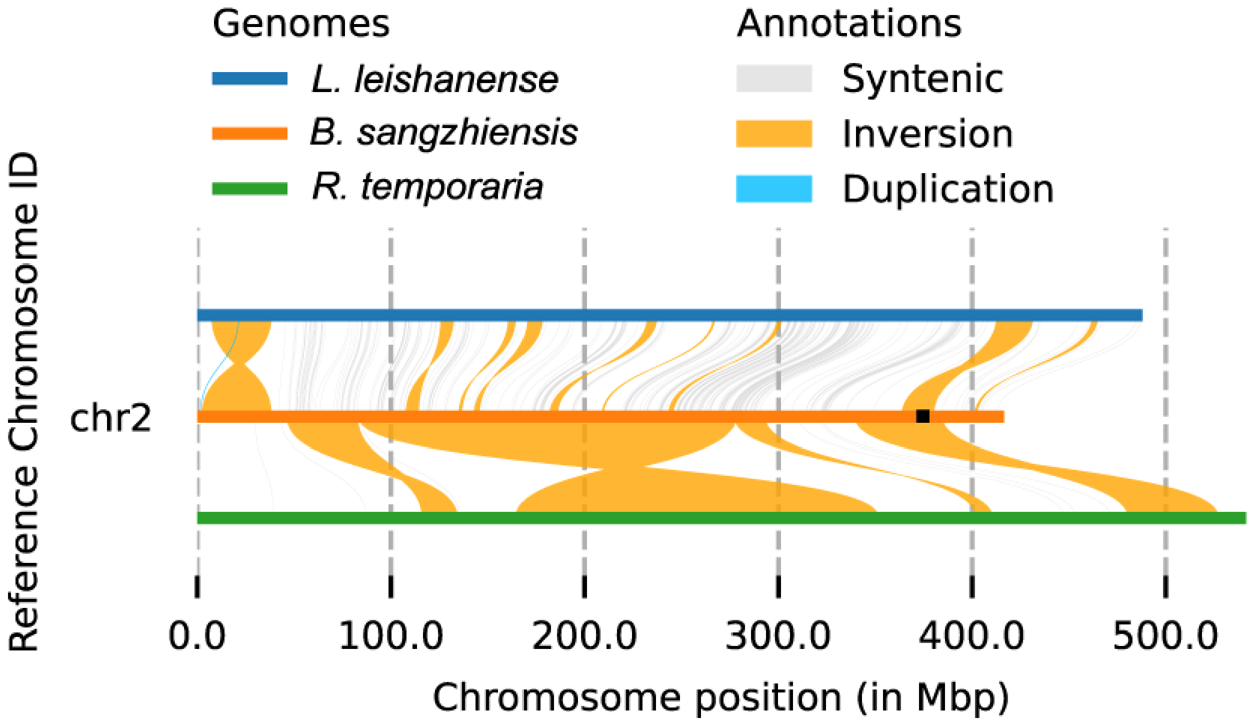


**Figure S27.** Chromosomal structural variants of *B. sangzhiensis*, *R. temporaria* and *L. leishanense*. Black region on chromosome was the sex-linked region.

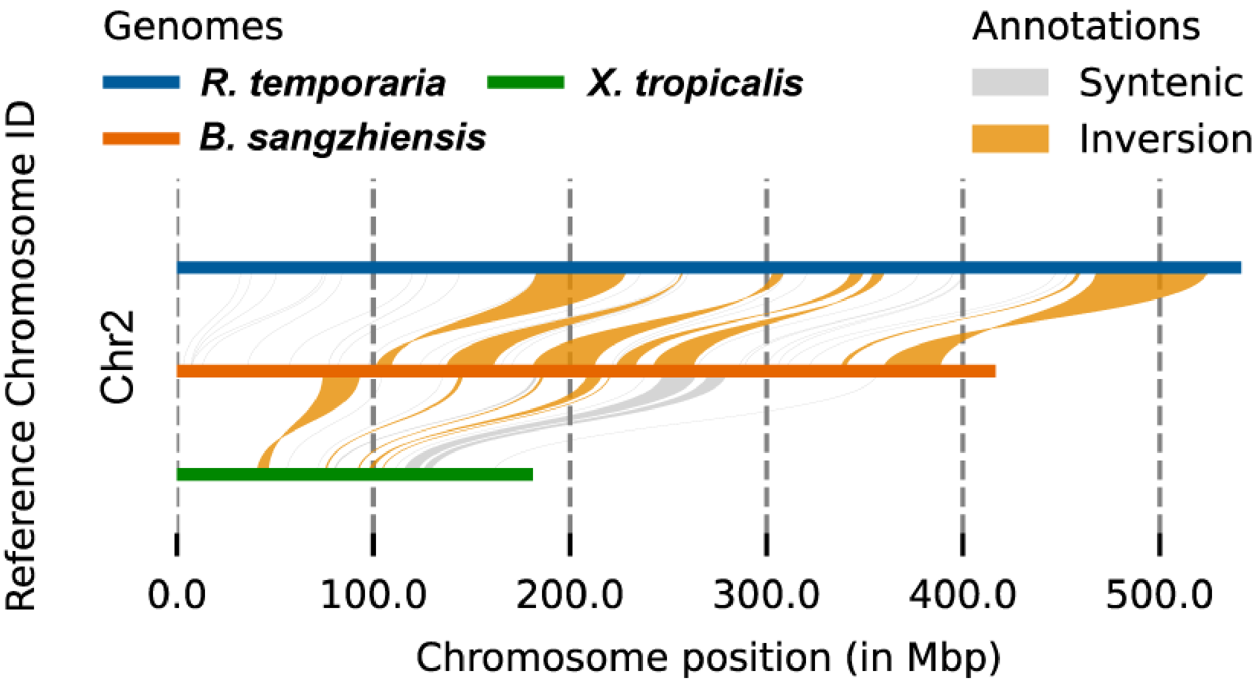


**Figure S28.** Chromosomal structural variants among *R. temporaria*, *B. sangzhiensis*, and *X. tropicalis.* Blue, orange, and green bars represent chromosomes from the three species, respectively. Grey ribbons indicate syntenic regions, while orange ribbons denote inversion events. Multiple inversions are observed between *R. temporaria* and *B. sangzhiensis*, whereas *X. tropicalis* shows lower overall synteny with the other species.

(a)

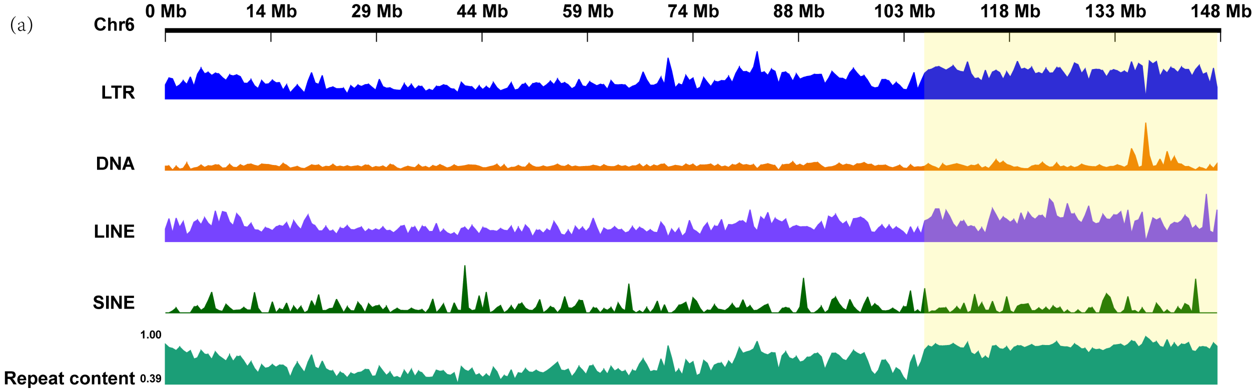


(b)

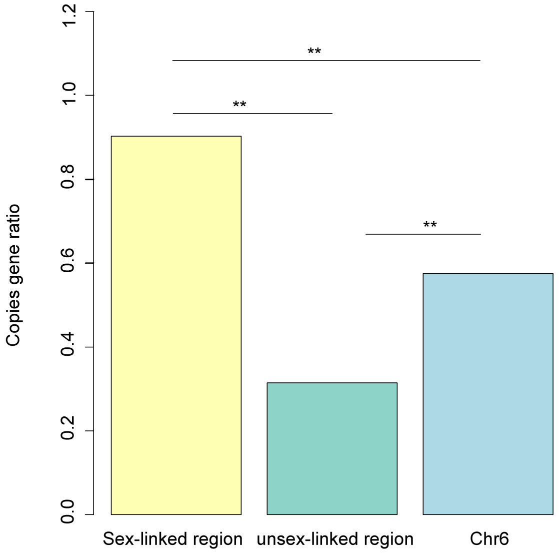


**Figure S29.** (a) The distribution of repetitive sequences. From top to bottom: LTR, DNA, LINE, SINE, and Repetitive elements contents. LTR is the mostly abundant repeat contents. (b). Gene copies rates of *B. sangzhiensis*, showing the genes from sex-linked region have significantly higher copies rates than those from other regions based on the two-sided Fisher's exact test. Copies gene ratio is calculated by dividing copies genes by total genes in specified chromosomes or sex-related regions, that is 0.576053757 (943/1637) for Chr 6 and 0.31469298245614 (287/912) for unsex-linked region on Chr 6, 0.903581267(656/726) for sex-linked region in *B. sangzhiensis.*

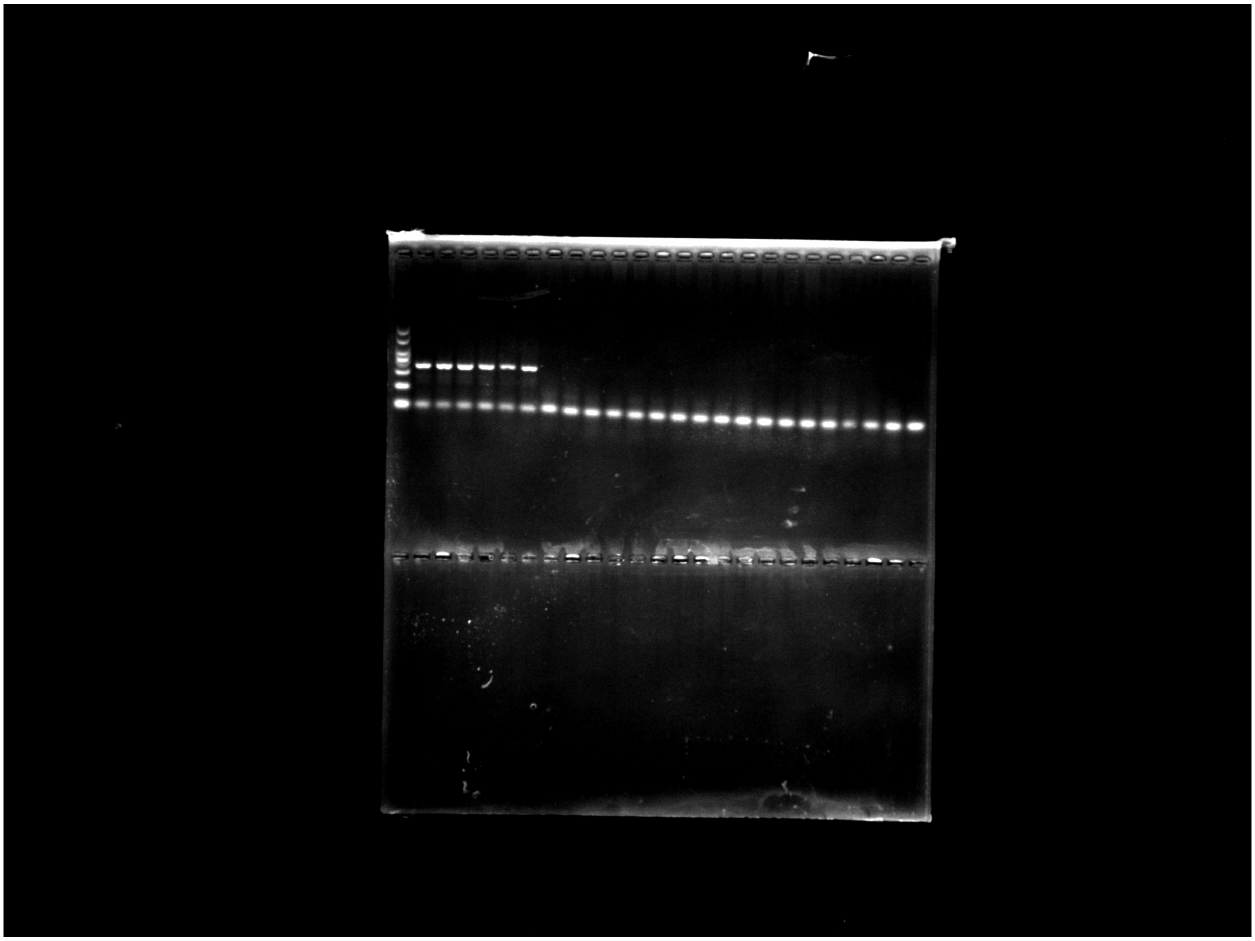

**Figure S30.** Unmodified original gel image corresponding to Figure 3d in the main text.
